# Targeting the KAT8/YEATS4 Axis Represses Tumor Growth and Increases Cisplatin Sensitivity in Bladder Cancer

**DOI:** 10.1002/advs.202310146

**Published:** 2024-03-25

**Authors:** Miner Xie, Liwen Zhou, Ting Li, Yujie Lin, Ruhua Zhang, Xianchong Zheng, Cuiling Zeng, Lisi Zheng, Li Zhong, Xiaodan Huang, Yezi Zou, Tiebang Kang, Yuanzhong Wu

**Affiliations:** ^1^ State Key Laboratory of Oncology in South China Collaborative Innovation Center for Cancer Medicine Guangdong Provincial Clinical Research Center for Cancer Sun Yat‐sen University Cancer Center Guangzhou 510060 P. R. China; ^2^ Department of Hematology Guangzhou First People's Hospital South China University of Technology Guangzhou 510060 P. R. China; ^3^ Center of Digestive Disease Scientific Research Center The Seventh Affiliated Hospital Sun Yat‐sen University Shenzhen 518107 P. R. China; ^4^ School of Medicine Shenzhen Campus of Sun Yat‐Sen University Shenzhen 518107 P. R. China

**Keywords:** KAT8, YEATS4, acetylation, bladder cancer, cisplatin sensitivity, DNA repair, tumor growth

## Abstract

Bladder cancer (BC) is one of the most common tumors characterized by a high rate of relapse and a lack of targeted therapy. Here, YEATS domain‐containing protein 4 (YEATS4) is an essential gene for BC cell viability using CRISPR‐Cas9 library screening is reported, and that HUWE1 is an E3 ligase responsible for YEATS4 ubiquitination and proteasomal degradation by the Protein Stability Regulators Screening Assay. KAT8‐mediated acetylation of YEATS4 impaired its interaction with HUWE1 and consequently prevented its ubiquitination and degradation. The protein levels of YEATS4 and KAT8 are positively correlated and high levels of these two proteins are associated with poor overall survival in BC patients. Importantly, suppression of YEATS4 acetylation with the KAT8 inhibitor MG149 decreased YEATS4 acetylation, reduced cell viability, and sensitized BC cells to cisplatin treatment. The findings reveal a critical role of the KAT8/YEATS4 axis in both tumor growth and cisplatin sensitivity in BC cells, potentially generating a novel therapeutic strategy for BC patients.

## Graphical abstract

KAT8‐mediated acetylation of YEATS4 prevents HUWE1‐mediated ubiquitination and degradation. Suppression of YEATS4 acetylation with the KAT8 inhibitor MG149 reduces cell viability and sensitizes BC cells to cisplatin treatment. This study reveals a critical role of the KAT8/YEATS4 axis in BC cells and potentially generates a novel therapeutic strategy for BC patients.

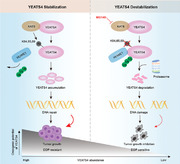

## 1 Introduction

Bladder cancer (BC) is one of the most common malignancies worldwide. The incidence and mortality of bladder cancer have been increasing annually.^[^ ^1^ ^]^ Although the identification of gene mutations, such as FGFR3^[^ ^2^ ^]^ and p53,^[^ ^3^ ^]^ and chromosome alterations^[^ ^4^ ^]^ in BC specimens has shed light on BC development, a specific targeted therapy is still lacking. Cisplatin (DDP)–based combination chemotherapies have become important adjuvant therapies for patients with invasive or metastatic BC. However, most patients with BC progressively develop DDP resistance.^[^ ^5^ ^]^ Therefore, the identification of new therapeutic targets for BC is urgently needed.

With a well‐conserved YEATS domain, YEATS domain‐containing proteins are of substantial biological interest, as they have been recently identified as novel readers of histone acetylation.^[^ ^6^ ^]^ In humans, there are 4 YEATS domain‐containing proteins, including AF9, ENL, YEATS4, and YEATS2.^[^ ^7^ ^]^ Particularly, ENL has been implicated in the transactivation of oncogenes that are crucial for acute leukemia development.^[^ ^8^ ^]^ Induction of ENL protein degradation or targeting the ENL YEATS domain leads to the suppression of oncogenic genes and reduction of cancer cell viability,^[^ ^8^, ^9^ ^]^ suggesting that the YEATS proteins might be the potential therapeutic targets for cancer therapies.

YEATS4, also known as GAS41, was originally found to be amplified in glioblastomas.^[^ ^10^ ^]^ Emerging studies have shown that YEATS4 is essential for cell survival, cell growth, and p53 pathway regulation.^[^ ^11^ ^]^ Despite realizing the critical role of YEATS4 in cancers, a targeting strategy for this protein remains unavailable due to the poor understanding of how YEATS4 is regulated in cancers. Here, we demonstrate that acetylation of YEATS4 by the histone acetyltransferase KAT8 stabilizes YEATS4 via impairing its binding to E3 ligase HUWE1 and promotes BC tumor growth and that targeting the KAT8/YEATS4 axis suppresses tumor growth and sensitizes BC cells to cisplatin.

## 2 Results

### 2.1 YEATS4 is Essential for the Tumor Growth of Bladder Cancer Cells

**Figure 1:**
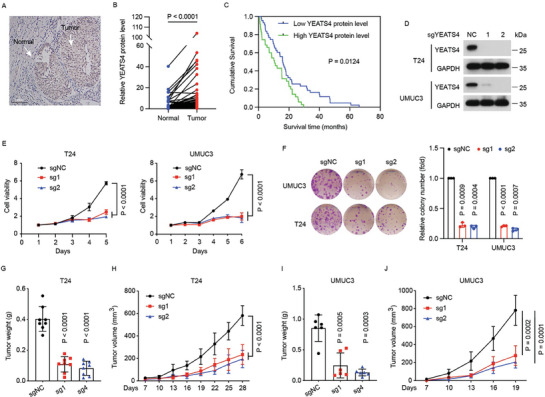
YEATS4 is essential for the tumor growth of bladder cancer cells. A) Representative immunohistochemical images of YEATS4 in 78 BC tissues. Scale bar, 100 µm. B) The relative YEATS4 protein level in paired tumor tissues (Tumor) and adjacent normal tissues (Normal) used in (A). C) Overall survival curves were analyzed based on YEATS4 protein levels as measured in (A and B) in BC patients. D) Western blotting of the indicated proteins in the indicated stable cells of 3 independent experiments. E) Cell viability of T24 and UMUC3 cells expressing YEATS4‐targeting sgRNAs was evaluated by MTT assay at the indicated time points. n = 3 independent experiments. F) Colony formation assays were performed for the indicated stable cells. Colony numbers were quantified using ImageJ software. *n* = 3 independent experiments. G–J) The indicated stable cells as shown in (D) were subcutaneously injected into nude mice. Tumor weights (G and I) and tumor volumes (H and J) were measured. *n* = 8 nude mice per group were used for T24 experiments, and *n* = 6 nude mice per group were used for UMUC3 experiments. Data in E‐J are presented as the mean ± SD. *P* values in C and E–J were calculated using the two‐tailed Student's *t*‐test. *p* values in C were analyzed using the Kaplan‒Meier plots.

To identify new therapeutic targets for BC, we recently performed CRISPR‒Cas9 screening using a lentivirus library with sgRNAs that target human transcription factors and epigenetic molecules in 3 bladder cancer cell lines (SW780, 5637 and T24), as aberrant gene transcription is the most important characteristic of BC progression.^[^ ^12^ ^]^ Among the candidate genes required for the survival and proliferation of BC cells, YEATS4 caught our attention (Figure S1A, Supporting Information), since YEATS4 was originally found to be amplified in glioblastomas^[^ ^13^ ^]^ and plays an important role in repressing the p53 tumor suppressor pathway during normal cellular proliferation.^[^ ^11a^ ^]^ In addition, data from The Cancer Genome Atlas (TCGA) and Cancer Dependency Map (DepMap) revealed that YEATS4 is amplified in a subset of BC patients (Figure S1B,C, Supporting Information) and is essential for the growth of various BC cells (Figure S1D, Supporting Information). Furthermore, immunohistochemical (IHC) staining of YEATS4 in 78 BC tumor tissues showed that YEATS4 was higher in tumor tissues than in adjacent normal tissues, and high YEATS4 protein levels were associated with shorter overall survival in BC patients (**Figure** **1**A–C).

To determine the roles of YEATS4 in BC, we knocked out YEATS4 by single guide RNAs (sgRNAs) in T24 and UMUC3 cells harboring relatively high levels of YEATS4 (Figure S1E, Supporting Information). Depletion of YEATS4 significantly reduced cell viability, colony formation, and tumor growth (Figure 1D–J). However, ectopic YEATS4 did not affect cell viability or colony formation in BIU87 and 5637 cells with relatively low levels of YEATS4 (Figure S1E–H, Supporting Information), indicating that endogenous YEATS4 is high enough to execute its functions in BC cells. These results revealed that YEATS4 plays an essential role in the tumor growth of BC cells.

### 2.2 YEATS4 is Required for DNA Repair in BC Cells

**Figure 2:**
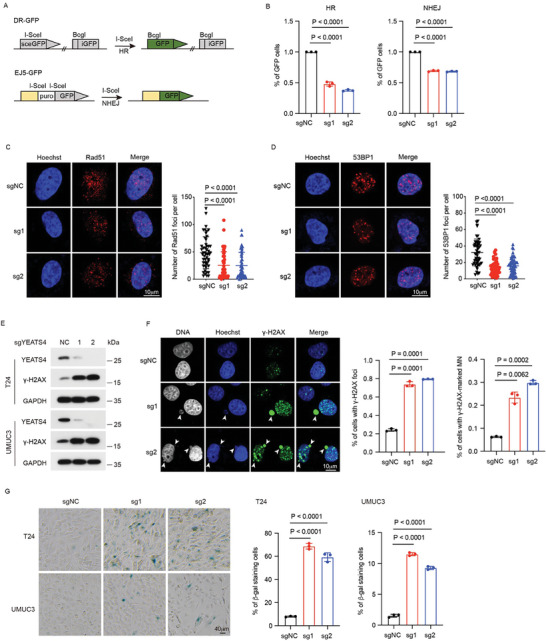
Depletion of YEATS4 inhibits DNA repair and induces cellular senescence. A) Schematic illustration of the GFP‐based HR reporter assay (DR‐GFP) and NHEJ reporter assay (EJ5‐GFP). B) Relative DNA repair efficiency in UMUC3 cells stably expressing the indicated sgRNAs. *n* = 3 independent experiments. C and D) Representative immunofluorescence images and quantification data of Rad51 foci (C) and 53BP1 foci (D) formation in T24 stable cells at 4 h after exposure to 10 Gy irradiation. *n* = 50 cells were analyzed for each group. Scale bar, 10 µm. E) γ‐H2AX levels in the indicated stable cells were analyzed by Western blotting. *n* = 3 independent experiments. F) Representative immunofluorescence images and quantification data of γ‐H2AX foci and micronuclei (MN) formation (pointed by arrows) in UMUC3 stable cells. *n* = 50 cells were analyzed for each group. Scale bar, 10 µm. G) Representative images and quantification data of senescence‐associated β‐galactosidase (SA‐β‐gal) staining of the indicated stable cells. *n* = 3 independent experiments. Data in B, C, D, F, and G are presented as the mean ± SD. *P* values were calculated using the two‐tailed Student's *t*‐test.

To explore how YEATS4 regulates the viability of BC cells, we performed RNA sequencing (RNA‐seq) analysis in YEATS4 knockout stable cells (Figure S2A,B and Table S1, Supporting Information). Reactome analysis showed that upregulated genes were enriched in several pathways, including cellular senescence, whereas downregulated genes were enriched in pathways regulating DNA repair and the cell cycle (Figure S2B, Supporting Information). The downregulation of a series of DNA repair‐related genes was validated by qRT‒PCR in cells depleted of YEATS4 (Figure S2C, Supporting Information), and by utilizing the DR‐GFP/EJ5‐GFP reporter system, knockout of YEATS4 significantly decreased both HR and NHEJ efficiency (**Figure** **2**A,B; Figure S2D, Supporting Information). Consistently, YEATS4 depletion significantly reduced the numbers of both Rad51 foci and 53BP1 foci (Figure 2C,D), as the formation of Rad51 foci^[^ ^14^ ^]^ and 53BP1 foci^[^ ^15^ ^]^ is an indicator of HR‐ and NHEJ‐mediated DSB repair, respectively. Consequently, the level of γ‐H2AX and the formation of micronuclei were increased, and senescence was induced in YEATS4‐depleted cells (Figure 2E–G). These results demonstrated that YEAST4 is critical for DNA repair in BC cells.

### 2.3 HUWE1 is Identified as an E3 Ubiquitin Ligase of YEATS4 via CRISPR Screening

**Figure 3:**
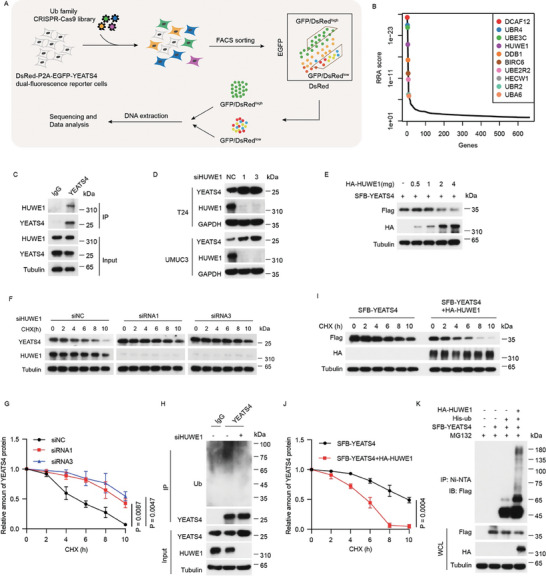
HUWE1 is an E3 ligase for the ubiquitination and degradation of YEATS4. A) Schematic of the YEATS4 Protein Stability Regulators Screening Assay (ProSRSA). B) Top 10 candidate genes of MAGeCK analysis. The x‐axis represents the ranking of genes, and the y‐axis indicates the robust rank aggregation (RRA) score of each gene. C) T24 cells were subjected to co‐IP by using anti‐YEATS4 or anti‐IgG to detect endogenous HUWE1. D) The indicated cells were transfected with HUWE1‐targeted siRNAs for 48 h and then were lysed and analyzed by Western blotting. E) HEK293T cells were transfected with indicated plasmids at the indicated concentration for 24 h and then were lysed and analyzed by Western blotting. F and G) T24 cells transfected with HUWE1‐targeted siRNAs for 48 h were treated with 40 µg mL^−1^ cycloheximide (CHX) at the indicated time points and then lysed and analyzed by Western blotting (F). Quantitation of YEATS4 protein levels was based on the Western blotting results (G). H) T24 cells transfected with HUWE1‐targeted siRNAs for 48 h were subjected to co‐IP with the indicated antibodies followed by Western blotting. I and J) HEK293T cells cotransfected with the indicated plasmids for 12 h were treated with 40 µg/ml CHX at the indicated time points, and then were lysed and analyzed by Western blotting (I). Quantification of YEATS4 protein levels was based on the Western blotting results (J). K) HEK293T cells cotransfected with the indicated plasmids for 48 h were incubated with 10 µM MG132 for 6 h, and then subjected to IP using Ni‐NTA beads followed by Western blotting. Ni‐NTA, nitrilotriacetic acid. Data are representative of *n* = 3 independent experiments. Data in G and J are presented as the mean ± SD. *P* values were analyzed using the two‐tailed Student's *t*‐test.

Since YEATS4 is elevated in BC tissues IHC (Figure 1B), we wondered whether its protein stability is tightly regulated. Indeed, we found that YEATS4 half‐life is very short in BC cell lines (Figure S3A, Supporting Information), indicating that it is an unstable protein and degradation dysregulation might contribute to its protein elevation in BC tissues. Therefore, we sought to identify the E3 ligase for YEATS4 protein degradation using the Protein Stability Regulators Screening Assay (ProSRSA) (**Figure** **3**A; Figure S3B, Supporting Information).^[^ ^16^ ^]^ Indeed, we observed that the levels of both exogenous EGFP‐YEATS4 and endogenous YEATS4 proteins were decreased and increased upon CHX and bortezomib (an inhibitor of proteasome) treatment, respectively (Figure S3C–F, Supporting Information). Then, combined with a CRISPR‒Cas9 library targeting the ubiquitin family genes, the top 10 candidates were identified via MAGeCK analysis^[^ ^17^ ^]^ (Figure 3A,B). Among these top genes, UBE3C, HUWE1, DDB1 and HECW1 were confirmed to be the potential E3 ligases for the YEATS4 protein, as knocking down each of them increased the YEATS4 protein level in both T24 and UMUC3 cells, and HUWE1 was the most significant (Figure S3G,H, Supporting Information). Therefore, HUWE1 was chosen for further investigation. The interaction between YEATS4 and HUWE1 was detected at both endogenous and exogenous levels (Figure 3C; Figure S3I, Supporting Information). Knockdown and overexpression of HUWE1 markedly increased and decreased YEATS4 protein levels, respectively (Figure 3D,E), but had no effect on YEATS4 mRNA levels in cells (Figure S3J–L, Supporting Information). Concordantly, the knockdown of HUWE1 by siRNAs prolonged the half‐life and reduced the ubiquitination of endogenous YEATS4 protein in cells, while ectopic HUWE1 shortened the half‐life and increased the ubiquitination of ectopic YEATS4 (Figure 3F–K). These results illustrated that HUWE1 acts as an E3 ubiquitin ligase for the ubiquitination and degradation of YEATS4.

### 2.4 KAT8 Inhibits Ubiquitination‐Mediated Proteasomal Degradation of YEATS4

**Figure 4:**
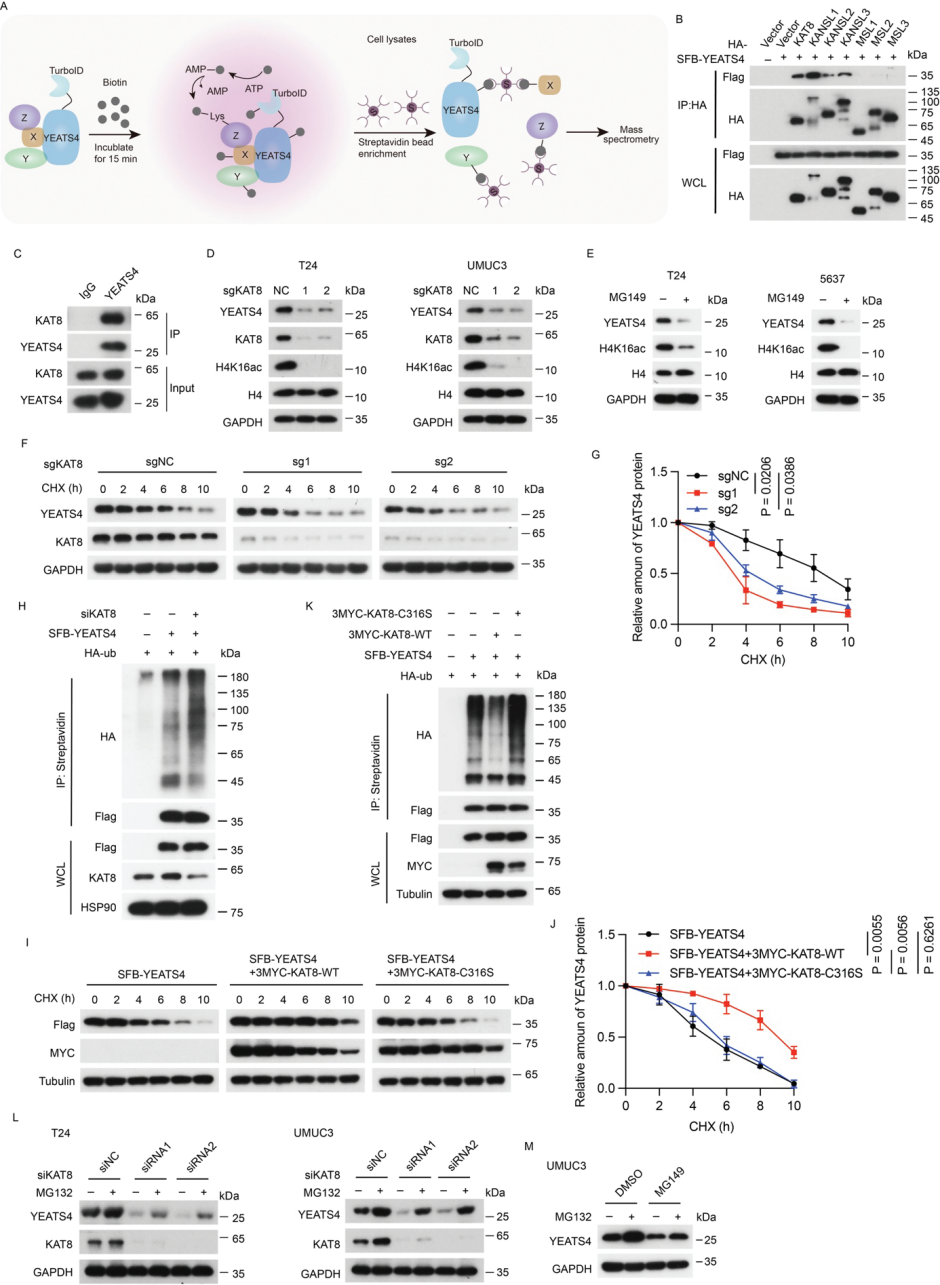
KAT8 inhibits ubiquitination‐mediated proteasomal degradation of YEATS4. A) Schematics of the proximity labeling assay using YEATS4‐V5‐TurboID‐construct. B) HEK293T cells were cotransfected with the indicated constructs for 48 h, and then subjected to IP using HA‐agarose beads followed by Western blotting. C) T24 cells were subjected to co‐IP by using anti‐YEATS4 or anti‐IgG to detect endogenous KAT8. D and E) Western blotting of the indicated proteins in the indicated stable cells infected with KAT8‐targeted sgRNAs (D) or treated with or without 50 µM MG149 for 48 h (E). Data are representative of 3 independent experiments. F and G) The indicated stable cells expressing KAT8‐targeted sgRNAs were treated with 40 µg/ml CHX at the indicated time points and then were lysed and analyzed by Western blotting (F). Quantitation of YEATS4 protein levels was based on the Western blotting results (G). H and K) HEK293T cells were cotransfected with the indicated plasmids for 48 h and then subjected to immunoprecipitation using streptavidin beads followed by Western blotting. WCL, whole cell lysate. I and J) HEK293T cells cotransfected with the indicated plasmids for 12 h were treated with 40 µg/ml CHX at the indicated time points and then were lysed and analyzed by Western blotting (I). Quantification of YEATS4 protein levels was based on the Western blotting results (J). L and M) The indicated cells transfected with KAT8‐targeted siRNAs for 48 h (L) or incubated with 50 µM MG149 for 48 h (M) were treated with or without 10 µM MG132 for 6 h, and then cells were lysed and analyzed by Western blotting. Data are representative of *n* = 3 independent experiments. Data in G and J are presented as the mean ± SD. *P* values were analyzed using the two‐tailed Student's *t*‐test.

We further investigated how YEATS4 is stabilized in bladder cancer at the posttranslational level, which may provide therapeutic opportunities. To this end, a proximity labeling assay coupled with mass spectrometry was performed (**Figure** **4**A). Among the mass spectrometry results (Table S2, Supporting Information), KANSL1 aroused our interest, since it is one of the core members of the nonspecific lethal (NSL) complex, which also contains KANSL2, KANSL3, and the catalytic subunit KAT8. In addition to the NSL complex,^[^ ^18^ ^]^ KAT8 alternatively interacts with MSL1, MSL2, and MSL3, forming the male‐specific lethal (MSL) complex.^[^ ^19^ ^]^ Our group recently reported that KAT8 acetylates IRF1 and promotes PD‐L1 expression, which enhances tumor immune evasion.^[^ ^20^ ^]^ In mammals, KAT8 plays critical roles in various cellular processes, including embryonic development,^[^ ^21^ ^]^ DNA repair,^[^ ^22^ ^]^ cell survival,^[^ ^23^ ^]^ chromatin architecture,^[^ ^24^ ^]^ and genome integrity.^[^ ^25^ ^]^

Based on the above background, we first validated the interaction between YEATS4 and the NSL and MSL complex subunits. Coimmunoprecipitation confirmed that YEATS4 interacted with the KAT8‐NSL complex (Figure 4B). In particular, the interaction between YEATS4 and KAT8 was detected at both endogenous and exogenous levels (Figure 4C; Figure S4A, Supporting Information). More importantly, KAT8 depletion or pharmacologic inhibition by MG149 significantly reduced the protein, but not mRNA, level of YEATS4 in cells (Figure 4D,E; Figure S4B, Supporting Information). Reduction of the YEATS4 protein upon KAT8 depletion might be a general mechanism as similar effects were observed in various cancer cell lines (Figure S4C, Supporting Information). These results suggested that KAT8 stabilizes YEATS4 in a posttranslational manner. Indeed, time‐course experiments showed that the knockout of KAT8 shortened the half‐life of endogenous YEATS4 (Figure 4F,G). In addition, knockdown of KAT8 increased the ubiquitination of ectopic YEATS4 (Figure 4H). In contrast, ectopic wild‐type (WT) KAT8 but not its C316S catalytically deficient mutant prolonged the half‐life and decreased the ubiquitination of ectopic YEATS4 protein (Figure 4I–K). In addition, treatment of the cells with the proteasome inhibitor MG132 but not lysosome inhibitor bafilomycin partially rescued the reduction in YEATS4 abundance caused by depletion of KAT8 or treatment with MG149 (Figure 4L,M; Figure S4D,E, Supporting Information). Taken together, these results revealed that KAT8 stabilizes the YEATS4 protein by preventing it from ubiquitination‐mediated proteasomal degradation.

### 2.5 KAT8‐Mediated Acetylation of YEATS4 Prevents its Proteasomal Degradation by Disrupting its Interaction with HUWE1

**Figure 5:**
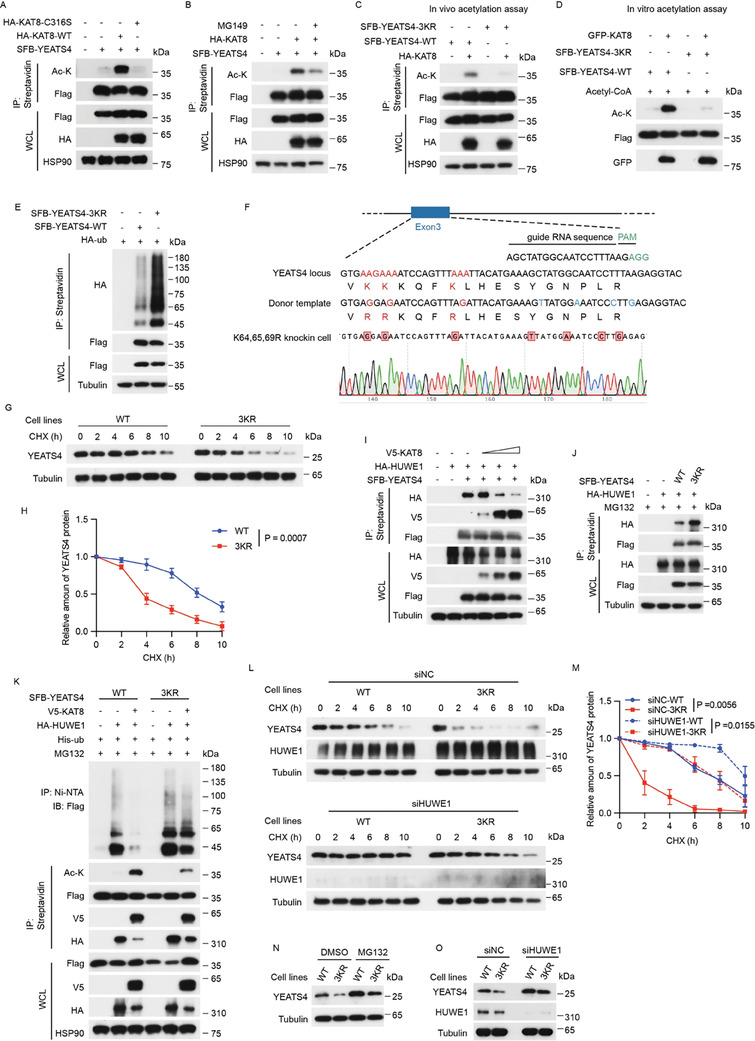
KAT8‐mediated acetylation of YEATS4 at K64, 65, and 69 prevents its proteasomal degradation by disrupting its interaction with HUWE1. A, C, and E) HEK293T cells were cotransfected with the indicated plasmids for 48 h and then were subjected to IP using streptavidin beads followed by Western blotting. B) HEK293T cells cotransfected with the indicated plasmids for 24 h were incubated with or without 50 µM MG149 for another 24 h, and then subjected to IP using streptavidin beads followed by Western blotting. D) Purified SFB‐YEATS4 WT or 3KR mutant proteins were incubated with or without the purified GFP‐KAT8 protein in vitro as described in the Methods section and then analyzed by Western blotting. F) Schematics of the locus‐specific YEATS4 K64,65,69R knock‐in UMUC3 cell lines constructed by CRISPR‒Cas9‐mediated genome editing. K64, K65, and K69 of YEATS4 were located within exon 3. An sgRNA efficiently targeting YEATS4 was used. The codons encoding K64, K65, and K69 are shown in red font in the WT locus (upper). A knock‐in DNA donor template containing the mutated codons of K64, K65, and K69 is shown in red font and the silent mutated codons of sgRNA are shown in blue font (middle). Site‐specific PCR amplification and Sanger sequencing of the K64, 65, and 69R knock‐in clones are shown (bottom). G and H) Parental YEATS4 WT cells and YEATS4 3KR cells were treated with 40 µg/ml CHX at the indicated time points and then were lysed and analyzed by Western blotting (G). Quantification of YEATS4 protein levels was based on the Western blotting results (H). I) HEK293T cells were cotransfected with the indicated plasmids for 48 h and then were subjected to IP using streptavidin beads followed by Western blotting. J) HEK293T cells cotransfected with the indicated plasmids for 48 h were treated with 10 µM MG132 for 8 h and then were subjected to IP using streptavidin beads followed by Western blotting. K) HEK293T cells cotransfected with the indicated plasmids for 48 h were treated with 10 µM MG132 for 8 h and then were subjected to IP using streptavidin beads or Ni‐NTA beads followed by Western blotting. L and M) YEATS4 WT and 3KR cells transfected with HUWE1‐targeted siRNAs for 48 h were treated with 40 µg/ml CHX at the indicated time points and then were lysed and analyzed by Western blotting (L). Quantification of YEATS4 protein levels was based on the Western blotting results (M). N and O) YEATS4 WT and 3KR cells were treated with or without 10 µM MG132 for 8 h (N) or transfected with HUWE1‐targeted siRNAs for 48 h (O), and then lysed and analyzed by Western blotting. Data in H and M are presented as the mean ± SD. of *n* = 3 independent experiments. *p* values were analyzed using the two‐tailed Student's *t*‐test.

As an acetyltransferase, KAT8 acetylates not only histone proteins but also non‐histone proteins and executes its function via acetylation of these substrates. Next, we wondered whether KAT8‐mediated YEATS4 stabilization relies on acetylation. Indeed, acetylation of YEATS4 was detected upon treatment with deacetyltransferase inhibitors trichostatin A (TSA) and nicotinamide (NAM) (Figure S5A, Supporting Information). The YEATS4 acetylation level was markedly increased in the presence of WT KAT8 but not its catalytically deficient C316S mutant (**Figure** **5**A). Likewise, pharmacologic inhibition of KAT8 by MG149 decreased the acetylation of YEATS4 (Figure 5B). These data suggested that YEATS4 is a substrate of KAT8. To identify the acetylation sites in YEATS4, we generated lysine‐to‐arginine (K‐to‐R) mutants in single or combinational lysine residues (Figure S5B, Supporting Information). Among the 12 mutants we generated, the acetylation of the K64, 65, 69R (3KR) mutant was much lower than that of either WT YEATS4 or other mutants in the presence of KAT8 (Figure S5C, Supporting Information). Further, mass spectrometry analysis validated the acetylation at the K64, K65, or K69 residue (Figure S5D, Supporting Information). Next, we generated single, double, or triple K‐to‐R mutants within these 3 lysine residues. The results showed that the combined mutation of K64, K65, and K69 resulted in the weakest acetylation level, indicating that all 3 lysine residues are the potential KAT8‐mediated acetylation sites (Figure 5C; Figure S5E, Supporting Information). In vitro, acetylation assay also confirmed that acetylation of the YEATS4 3KR mutant was much lower than that of WT YEATS4 (Figure 5D). In addition, we demonstrated that these 3 acetylation sites were specifically catalyzed by KAT8 but not other tested acetyltransferases (Figure S5F,G, Supporting Information).

Interestingly, in contrast to the decreased acetylation level, we found that the ubiquitination level of the YEATS4 3KR mutant was significantly increased compared with WT YEATS4 (Figure 5E), suggesting that KAT8‐mediated YEATS4 acetylation contributes to YEATS4 protein stability. To further confirm this assumption, we generated locus‐specific K64,65,69R knock‐in UMUC3 cell lines (hereafter termed 3KR cells) using CRISPR‒Cas9‐mediated homology‐directed repair (**Figure** **5**F). Time course experiments revealed that the half‐life of the endogenous YEATS4 protein in 3KR cells was much shorter than that in parental UMUC3 cells (hereafter termed WT cells) (**Figure** **5**G,H).

The above observations prompted us to determine whether KAT8‐mediated YEATS4 acetylation antagonizes HUWE1‐dependent YEATS4 ubiquitination and degradation. To test this notion, several coimmunoprecipitation (Co‐IP) assays were performed. Co‐IP with YEATS4, HUWE1, and KAT8 showed that KAT8 diminished the interaction between YEATS4 and HUWE1 in a dose‐dependent manner (Figure 5I). In addition, cotransfection of HUWE1 and YEATS4 showed that HUWE1 bound more strongly to the YEATS4 3KR mutant than to the WT YEATS4 (Figure 5J). These results indicated that KAT8‐catalyzed YEATS4 acetylation impaired YEATS4 binding to HUWE1. As expected, the ubiquitination of WT YEATS4, but not the YEATS4 3KR mutant, mediated by HUWE1 was dramatically decreased in the presence of KAT8 (Figure 5K). Concordantly, the knockdown of HUWE1 also prolonged the half‐life of endogenous YEATS4 in 3KR mutant cells (Figure 5L,M). Similar to the treatment of cells with the proteasome inhibitor MG132, the knockdown of HUWE1 in 3KR mutant cells partially rescued the decreased YEATS4 protein abundance (Figure 5N,O). Collectively, our findings determined that KAT8‐mediated acetylation of YEATS4 at K64, 65, and 69 prevents its proteasomal degradation by disrupting its interaction with HUWE1.

### 2.6 Acetylation of YEATS4 by KAT8 is Critical for its Oncogenic Function in Bladder Cancer

**Figure 6:**
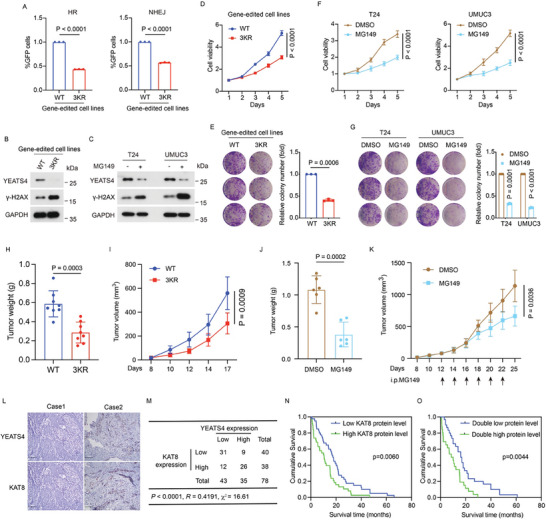
Acetylation of YEATS4 by KAT8 is critical for its oncogenic function in bladder cancer cell viability. A) Relative DNA repair efficiency in YEATS4 WT and 3KR cells. B) Western blotting of the indicated proteins in YEATS4 WT and 3KR cells. *n* = 3 biologically independent experiments. C) T24 and UMUC3 cells were treated with or without 50 µM MG149 for 48 h and then were lysed and analyzed by Western blotting. *n* = 3 biologically independent experiments. D and E) MTT assays (D) and colony formation assays (E) were performed in YEATS4 WT and 3KR cells. F and G) MTT assay (F) and colony formation assay (G) were performed in T24 and UMUC3 cells treated with DMSO or 50 µM MG149. H and I) Tumor growth of YEATS4 WT and 3KR cells was evaluated in vivo. *n* = 8 nude mice per group. Tumor weights (H) and tumor volumes (I) were measured. J and K) Mice bearing UMUC3 tumors were randomly divided into the indicated groups (*n* = 6 mice per group). DMSO or MG149 (5 mg kg^−1^) was injected intraperitoneally at the indicated time points. Tumor weights (J) and tumor volumes (K) were measured. L) Representative immunohistochemical staining images of both YEATS4 and KAT8 in 78 paraffin‐embedded BC tissues. Scale bar, 100 µm. M) Crosstab shows the distribution of cancer tissues in the 78 bladder cancer tissues used in (L) according to the H‐Score of YEATS4 and KAT8. *p *< 0.0001, χ^2^ tests. *R*, Spearman correlation coefficient. *P* values were analyzed using Pearson's chi‐squared test, and the *R*‐value was analyzed using Spearman's correlation test. N and O) Overall survival curves were generated based on the protein levels of YEATS4 or KAT8 in the BC tissues used in (L). *p* value was calculated using the Kaplan‒Meier plots. Data in A and D‐K are presented as the mean ± SD. *p* values were calculated by two‐tailed Student's *t*‐test.

We further investigated the biological function of YEATS4 acetylation in BC cells. By using the DR‐GFP/EJ5‐GFP reporter assay (Figure 2C), we found that both HR and NHEJ DNA repair efficiency were significantly reduced in acetylation‐deficient 3KR cells compared with WT cells (**Figure** **6**A). Consistent with this, the levels of γ‐H2AX were increased in 3KR cells (Figure 6B). Similarly, treatment of cells with MG149 also increased the level of γ‐H2AX (Figure 6C). These data suggested that YEATS4 acetylation is required for DNA repair. It has been well‐established that moderate cellular stress, such as genomic instability, is beneficial for cancer fitness whereas excessive genotoxic stress is deleterious for tumor cells.^[^ ^25^, ^26^ ^]^ Supporting this notion, we found that 3KR cells showed poorer cell viability and colony formation potential than WT cells (Figure 6D,E). Treatment with MG149 also significantly reduced the viability and colony formation of BC cells (Figure 6F,G). Furthermore, mice bearing 3KR cells exhibited a marked reduction in tumor size and tumor weight (Figure 6H,I). Consistently, administration of MG149 also suppressed tumor growth in mice (Figure 6J,K). Using the clinical BC tissues, a positive correlation between YEATS4 and KAT8 was detected (Figure 6L,M). And high expression of either YEATS4 or KAT8 was associated with poor overall survival in BC patients, while combinational analysis was more significant (Figure 1C and Figure 6N,O). Altogether, these results demonstrated that the acetylation of YEATS4 by KAT8 is critical for its oncogenic function in BC.

### 2.7 Suppression of YEATS4 Acetylation Sensitizes BC Cells to Cisplatin

**Figure 7:**
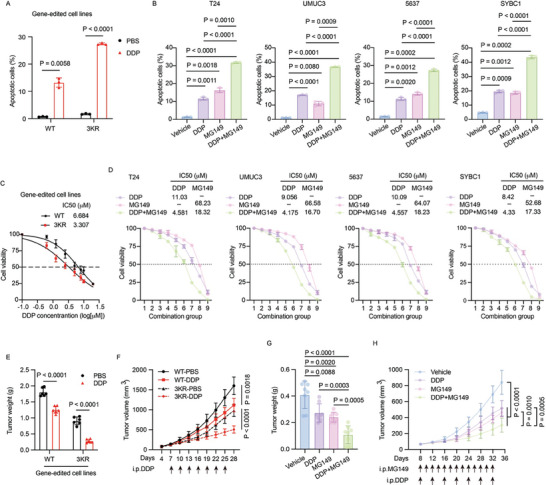
Disruption of KAT8‐mediated YEATS4 acetylation increases the DDP sensitivity of BC cells. A) Apoptosis in parental WT or 3KR cells treated with 5 µM DDP for 48 h. B) Apoptosis in the indicated cells treated with 2.5 µM DDP, 50 µM MG149, and their combination for 48 h. C) YEATS4 WT cells or 3KR cells were treated with the indicated concentrations of DDP for 48 h, and then cell viability was measured by MTT assay. *n* = 3 biologically independent experiments. D) The indicated cell lines were treated with the indicated concentrations of DDP, MG149, and their combination for 48 h, and then cell viability was measured by MTT assay. *n* = 3 biologically independent experiments. E and F) Mice bearing parental WT or 3KR tumors were randomly divided into 2 indicated groups (n = 6 mice per group). PBS or DDP (2 mg kg^−1^) was injected intraperitoneally at the indicated time points. Tumor weights (E) and tumor volumes (F) were measured. G and H) Mice bearing T24 tumors were randomly divided into 4 indicated groups (n = 8 mice per group). DDP (2 mg kg^−1^), MG149 (5 mg kg^−1^), and their combination were injected intraperitoneally at the indicated time points. Tumor weights (G) and tumor volumes (H) were measured. Data in A‐H are presented as the mean ± SD. *p* values were calculated by two‐tailed Student's *t*‐test. The IC50 values in C and D were calculated using GraphPad software with Nonlinear regression, Dose‐response‐Inhibiton, log (inhibitor) versus normalized response Variable slope methods.

Given the critical role of the KAT8/YEATS4 axis in BC, we wondered whether targeting YEATS4 acetylation contributes to BC therapy. DDP‐based chemotherapy is the first‐line treatment for patients with muscle‐invasive and metastatic bladder cancers, yet the clinical efficacy is unsatisfying due to acquired DDP resistance.^[^ ^5^, ^27^ ^]^ Interestingly, the DDP‐induced cell apoptosis was significantly increased in YEATS4 3KR cells compared to YEATS4 WT cells (**Figure** **7**A; Figure S6A, Supporting Information). Likewise, DDP+MG149 could enhance the DDP‐induced cell apoptosis in cells, including SYBC1, a patient‐derived cell line (PDC),^[^ ^28^ ^]^ compared to those treated with DDP or MG149 alone (Figure 7B; Figure S6B, Supporting Information). These results indicated that cells with YEATS4 acetylation deficiency were more sensitive to DDP. Concordantly, the half maximal inhibitory concentration (IC_50_) of DDP was much lower in YEATS4 3KR cells than in YEATS4 WT cells (Figure 7C). Similarly, the combination of DDP with MG149 significantly suppressed cell viability compared with each single treatment. More importantly, either the IC_50_ of DDP or MG149 was reduced in cells when they were used simultaneously (Figure 7D; Figure S6C, Supporting Information), indicating a strong combination effect between DDP and MG149. Next, we tested these effects in vivo. As shown in Figure 7E,F, tumors in mice bearing 3KR cells responded more effectively to DDP administration. Likewise, combinational treatment with DDP and MG149 showed the strongest effect on the suppression of tumor growth in mice (Figure 7G,H). In conclusion, these results determined that inhibition of YEATS4 acetylation by the KAT8 inhibitor sensitized BC cells to DDP treatment.

## 3 Discussion

**Figure 8:**
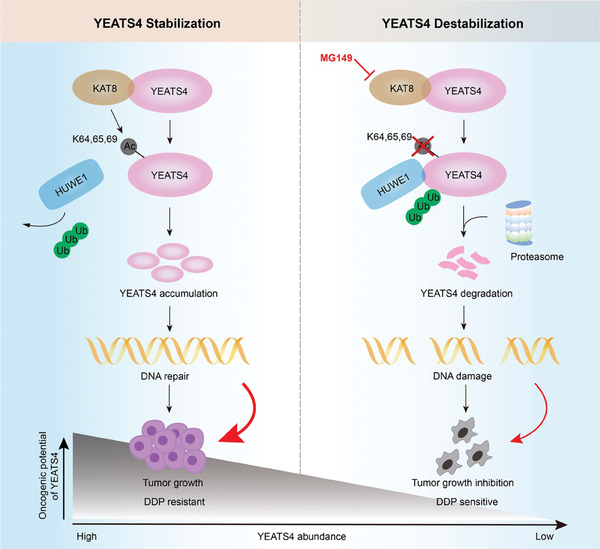
A proposed model for the function of the KAT8/YEATS4 axis in BC. KAT8‐medicated YEATS4 acetylation at K64, 65, and 69 blocks the binding of HUWE1 to YEATS4 and therefore promotes YEATS4 stabilization, which contributes to DNA repair and tumor growth. Inhibition of KAT8 by MG149 suppresses YEATS4 acetylation and leads to increased HUWE1 binding with YEATS4 and proteasomal degradation, which enhances genome instability and inhibits BC cell growth. Blockade of YEATS4 acetylation by MG149 increases DDP sensitivity and accelerates cell death in BC.

In this study, we uncovered that the KAT8/YEATS4 axis is essential for BC. Specifically, in BC cells, KAT8‐mediated YEATS4 acetylation at K64, 65, and 69 promotes YEATS4 stabilization by blocking its HUWE1‐dependent degradation, which in turn contributes to DNA repair and tumor growth and consequently results in DDP resistance. Inhibition of KAT8 by MG149 suppresses YEATS4 acetylation, leading to an increase in HUWE1 binding to YEATS4, and therefore facilitating HUWE1‐mediated YEATS4 ubiquitination and proteasomal degradation, which subsequently reduces DNA repair and tumor growth and enhances DDP sensitivity (**Figure** **8**).

YEATS4 belongs to a protein family that contains a well‐conserved YEATS domain, which has been recently identified as a novel reader of histone acetylation.^[^ ^6^, ^7^, ^8^ ^]^ The structure of these protein families consists of a YEATS domain, which is required for the recognition of acetylated histones and chromatin recruitment, as well as a coil‐coiled domain or/ and A box, which is involved in protein‐protein interactions.^[^ ^7^ ^]^ Previous studies have revealed a strong link between YEATS domain family members and human cancers, in which the YEATS domain was proven to be indispensable for their oncogenic function.^[^ ^8^, ^11^, ^29^ ^]^ Despite the realization of the importance of YEATS proteins in human cancers, how these proteins are regulated remains poorly known. In this study, we found that acetylation of YEATS4 by KAT8 promotes YEATS4 protein stabilization, which contributes to DNA repair and BC tumor growth. More importantly, disruption of YEATS4 acetylation inhibited tumor growth and sensitized BC cells to DDP. These findings implied that dysregulated posttranslational modification of YEATS4 may cause aberrant YEATS4 abundance and consequently result in elevated tumor growth and chemoresistance in BC. Our findings give new insights into how YEATS4 is regulated and provide a potential therapeutic strategy for BC patients.

KAT8 is a highly conserved MYST family HAT. In mammals, KAT8 specifically acetylates H4K16ac as well as nonhistone proteins, which play important roles in various cellular processes, such as DNA repair and genome stability.^[^ ^22^, ^24^, ^30^ ^]^ In this study, we report that YEATS4 is a new substrate of KAT8 and that HUWE1 is an E3 ligase responsible for YEATS4 proteasomal degradation. Interestingly, acetylation of YEATS4 at K64, 65, and 69 by KAT8 promotes YEATS4 protein stabilization by disrupting the binding of HUWE1 to YEATS4, supporting the notion that crosstalk between acetylation and ubiquitination affects protein stability.^[^ ^31^ ^]^ As lysine residues are sites of both acetylation and ubiquitination, competition‐based modification at the same lysine residues has been suggested to be one of the mechanisms involved in the control of protein stability following lysine acetylation.^[^ ^32^ ^]^ However, in our study, the ubiquitination levels of acetylation‐deficient YEATS4 3KR were much higher than those of WT YEATS4, excluding the possibility that K64, 65, 69 acetylation competes with K64, 65, 69 ubiquitination. Alternatively, YEATS4 K64, 65, and 69 acetylation counteracts its ubiquitination by affecting the binding affinity of YEATS4 to HUWE1. Further studies should be carried out to determine the precise ubiquitination site of YEATS4 mediated by HUWE1.

The roles of YEATS4 and KAT8 in promoting tumor growth have been reported previously.^[^ ^25^, ^29^ ^]^ However, whether they are potential prognostic markers and drug targets remains unknown. Here in this study, we demonstrated that high expression of both YEATS4 and KAT8 was associated with poor overall survival of BC patients. In clinical settings, DDP‐based combination chemotherapies have become important adjuvant therapies for patients with invasive or metastatic BC. Unfortunately, most patients who initially respond to DDP eventually develop resistance. Enhanced DNA repair or inhibition of apoptosis are potential mechanisms of DDP resistance.^[^ ^33^ ^]^ Interestingly, in our study, blockade of YEATS4 acetylation at K64, 65, 69 by pharmacological inhibition of KAT8 with MG149 impaired DNA repair efficiency and increased DDP‐induced apoptosis of BC cells and consequently sensitized BC cells to DDP. These findings emphasize the importance of YEATS4 acetylation in BC and reveal that targeting the KAT8/YEATS4 axis may be beneficial for BC patients with chemoresistance. However, due to the lack of specific YEATS4 K64, 65, and 69 acetylation antibodies, we could not validate the YEATS4 acetylation levels in BC patients especially those who developed chemoresistance, so we directly detected the total YEATS4 level as an alternative since the acetylation and total protein levels are positively correlated.

In summary, our study demonstrated that dysregulation of the posttranslational modification of YEATS4 may be the major cause of YEATS4 upregulation in BC. Therefore, suppression of YEATS4 acetylation by targeting the KAT8/YEATS4 axis may be a promising therapeutic intervention for BC patients.

## 4 Experimental Section

### Study Approval

The animal experiments were approved by the Animal Research Committee of Sun Yat‐sen University Cancer Center (L102012020100K) and were performed following the established guidelines and principles. The paraffin‐embedded bladder cancer tissues were obtained from the Department of Pathology and were approved by the Ethics Committee of Sun Yat‐sen University Cancer Center (G2023‐189‐01).

### Cell Culture

Cell lines T24, UMUC3, 5637, J82, TCCSUP, SCaBER, SW780, HT1376, RT4, and HEK293T embryonic kidney cells were obtained from the American Type Culture Collection (ATCC), RT112 were obtained from the European Collection of Authenticated Cell Cultures (ECACC), and the BIU87 cell line was obtained from Kunming Cell Bank, Chinese Academy of Sciences. Patients‐derived cells SYBC1 cell was a gift from Dr. Zhuowei Liu. All cell lines were validated by short‐tandem repeat DNA profiling analysis and cultured in Dulbecco's modified Eagle's medium supplemented with 10% fetal bovine serum and 1% penicillin/streptomycin at 37 °C and 5% CO2.

### Plasmid Construction

SFB‐tagged YEATS4 was cloned and inserted into the pSIN‐EF1α‐puro vector or PCDNA3.1 vector. The 3MYC‐tagged KAT8 and HA‐tagged KAT8, P300, CBP, TIP60, GCN5, and PCAF were cloned and inserted into the pSIN‐EF1α‐puro vector. The V5‐tagged KAT8 was cloned and inserted into the PCDNA3.1 vector. The PCDNA3.1‐HA‐HUWE1 plasmid was a gift from Prof. Genze Shao (Peking University). YEATS4 KR mutants and KAT8 C316S constructs were generated from WT YEATS4 and KAT8, respectively, with a one‐step cloning kit (Vazyme, C113‐02), and the sequences were verified by Sanger sequencing.

The lentiCRISPR‐V2‐puro vector was used to clone the sgRNAs targeting YEATS4 and KAT8. The sequences used for cloning the indicated sgRNAs are shown in Table S1, Supporting Information.

### MTT Assay

The indicated cell lines were seeded in 96‐well plates (2‐3 × 10^3^ cells per well) for 24 h. Cells were incubated with 0.5 mg/ml 3‐(4,5‐dimethylthiazol‐2‐yl)−2,5‐diphenyltetrazolium bromide (MTT) substrate for 4 h at 37 °C. After discarding the medium, DMSO was then added to each well for 10 min. The optical density (OD) was measured at 490 nm with a microplate reader once per day for 5 days.

### Colony Formation Assay

Cells were seeded into 24‐well plates (500 cells per well) and cultured for 8–10 days until visible colonies were formed. The colonies were fixed with paraformaldehyde and stained with 1% crystal violet overnight. The colony numbers were counted using ImageJ.

### DNA Repair Assays

DR‐GFP and EJ5‐GFP reporter systems were used to estimate repair efficiency.^[^ ^34^ ^]^ Briefly, 3 × 10^4^ UMUC3 cells stably expressing the indicated sgRNAs were seeded in 6 ‐well plates. Twenty‐hour hours later, the cells in each well were transfected with 200 µL opti‐MEM supplemented with plasmids containing 300 ng of DR‐GFP (or 300 ng of EJ5‐GFP), 1 µg of I‐Sce1 and 300 ng of mCherry. At 48 h after transfection, cells were collected and analyzed by flow cytometry. The relative percentage of GFP‐positive cells was calculated as follows: Repair efficiency (%) = mCherry and GFP double‐positive cells/mCherry positive cells × 100%.

### Immunofluorescence

Cells were fixed with 4% paraformaldehyde for 15 mins, washed with PBS twice, and then permeabilized with 0.5% Triton X‐100 for 10 mins. Subsequently, cells were blocked with serum for 30 min at room temperature followed by incubation with primary antibodies at 4 °C overnight. Cells were next washed with PBS 3 times and incubated with secondary antibodies for 1 h at room temperature. Cell nuclei were stained with Hoechst 33342 (Thermo Fisher, H3570) for 3 mins. After washing with PBS, cells were mounted (Beyotime, P0128M) and confocal images were acquired using a laser scanning microscope. Primary antibodies used in this study included anti‐γ‐H2AX (Cell Signaling Technoloby, 9718), anti‐53BP1 (Cell Signaling Technoloby, 4937S), and anti‐Rad51 (abcam, ab88572). Secondary antibodies included Donkey anti‐Rabbit IgG, Alexa Fluor 488 conjugate (Invitrogen, A21206), Goat anti‐Rabbit IgG, Alexa Fluor 594 conjugate (Invitrogen, A11037), and Goat anti‐Mouse IgG, Alexa Fluor 594 conjugate (Invitrogen, A11005)

### SA‐β‐gal Staining Assay

SA‐β‐gal staining assay was performed following the manufacturer's instructions (Beyotime Technology, China). Briefly, the indicated cells were obtained and stained overnight for SA‐β‐gal activity at 37 °C. Images were acquired and analyzed.

### Proximity Labeling Assay

The proximity labeling assay was performed as described previously.^[^ ^35^ ^]^ Briefly, to label YEATS4‐interacting proteins, cells expressing YEATS4‐V5‐TurboID were treated with 50 µM biotin for 30 min. Then, the cells were lysed in RIPA‐SDS buffer (150 mM NaCl, 0.125% SDS, 0.125% sodium deoxycholate, 1% Triton X‐100, and 50 mM Tris‐HCl pH 7.5). After sonication, the lysates were centrifuged at 12000 × g at 4 °C for 15 min, and the supernatant was incubated with streptavidin beads at 4 °C overnight. The beads were washed once with 1 M KCl buffer, once with 0.1 M Na_2_CO_3_ buffer, once with 2 M urea in 10 mM Tris buffer, and twice with RIPA‐SDS buffer. Then, the beads were resuspended in a 5× loading buffer, boiled, and subjected to mass spectrometry analysis.

### Western Blotting and Coimmunoprecipitation (Co‐IP)

Briefly, cells were collected and lysed in RIPA buffer (150 mM NaCl, 5 mM EDTA, 0.5% NP40, 50 mM Tris‐HCl, pH 8.0) supplemented with protease inhibitors and phosphatase inhibitors (Roche). Cell lysates were centrifuged for 15 min at 12,000 x g at 4 °C. Equal amounts of harvested total proteins were loaded and resolved by 10–12% sodium dodecyl sulfate‐polyacrylamide gradient gel. The proteins were then transferred to polyvinylidene difluoride membranes and blocked with 5% nonfat milk for 1 h at room temperature. The membranes were incubated with the indicated primary antibodies followed by horseradish peroxidase‐conjugated secondary antibodies. The protein signals were detected using the Western ECL Substrate.

For co‐IP, the clarified supernatants were incubated with the indicated beads for 2 h overnight at 4 °C. To detect the interaction between endogenous YEATS4 and KAT8 or HUWE1, the clarified supernatants were first incubated with anti‐YEATS4 (Santa Cruz, 1:200) supplemented with protein A/G agarose beads at 4 °C overnight. The immunoprecipitated beads were washed 5 times with RIPA buffer and subjected to SDS‐PAGE followed by Western blotting.

### RNA Interference Treatment

RNAi‐mediated knockdown of KAT8 or HUWE1 was performed by transfecting siRNA oligonucleotides into the indicated cell lines using Lipofectamine RNAi MAX according to the manufacturer's instructions. The oligonucleotide sequences targeting KAT8 or HUWE1 mRNA used in this study are listed in Table S3 (Supporting Information).

### Real‐Time RT‐PCR

Total RNA was extracted using a total RNA purification kit (TIANGEN) and the reverse‐transcription reaction was performed with the HiScript III 1st Strand cDNA synthesis kit (Vazyme). Real‐time RT‒PCR reaction was performed using SYBR Green master mix (Vazyme) on a LightCycler 480 Instrument II (Roche). GAPDH was used to normalize transcript abundance and the relative mRNA expression of genes was calculated using the ddCt method. All procedures were performed according to the manufacturer's instructions. The sequences of the primers are listed in Table S4 (Supporting Information).

### Flow Cytometry

To prepare the cells for flow cytometry sorting, live cells were harvested and centrifuged at 1000 r.p.m for 3 min. After discarding the supernatant, the cell pellets were resuspended in phosphate‐buffered saline (PBS) supplemented with 10% fetal bovine serum and filtered using a 70‐µm cell strainer. Cell sorting was performed using a Beckman MoFlo Cell Sorting System (Beckman Coulter, Brea, CA, USA), and flow cytometry analysis was performed using a Beckman Coulter cytoFLEX and CytExpert software.

### Ubiquitination Assay

HEK293T cells were transfected with the indicated constructs together with His‐tagged ubiquitin (His‐Ub) expressing plasmid for 24 h. Cells were treated with 10 µM MG132 for 6 h and then were lysed with buffer A (6 M guanidine‐HCl, 0.1 M Na2HPO4/NaH_2_PO_4_, 10 mM imidazole pH 8.0). After sonication, cell lysates were incubated with Ni‐NTA beads (Beyotime) at room temperature for 3 h. Subsequently, the His‐ubiquitinated proteins were washed 3 times with buffer A, twice with buffer A/TI (1 volume buffer A and 3 volumes buffer TI), and once with buffer TI (25 mM Tris‐HCl and 20 mM imidazole pH 6.8). The His‐ubiquitinated proteins were then subjected to SDS‐PAGE and Western blotting.

### Detection of YEATS4 Acetylation Site by Mass Spectrometry

To identify the acetylated lysine residues of YEATS4, HEK293T cells were cotransfected with SFB‐YEATS4 and HA‐KAT8 for 24 h and treated with 5 µM TSA plus 5 mM NAM for another 24 h. Afterward, cells were lysated and SFB‐YEATS4 proteins were first enriched by streptavidin beads at 4 °C for 4 h. After elution by biotin (2 mg/ml) for 4 h, SFB‐YEATS4 proteins were secondary enriched with Acetylated‐lysine antibody (Cell Signaling Technology, 9441S) supplemented with protein A/G agarose beads at 4 °C overnight. The immunoprecipitated beads were washed 5 times with RIPA and subjected to SDS‐PAGE. The band corresponding to ≈25–45 kDa was excised and subjected to mass spectrometry.

### Immunohistochemical Staining (IHC)

Paraffin‐embedded sections were cut at a thickness of 3 µm. After deparaffinization and dehydration, antigen retrieval was performed in EDTA‐Tris (pH 9.0). The tissue slides were then blocked with goat serum at room temperature for 30 min, followed by incubation with anti‐YEATS4 diluted 1:100 (Santa Cruz, sc‐393708) or anti‐KAT8 diluted 1:1000 (Abcam, ab200660) at 4 °C overnight. After 3 washes with PBS, endogenous peroxidase activity was blocked with 3% H_2_O_2_ for 10 min. Subsequently, the tissue slides were incubated with anti‐mouse/rabbit IgG secondary antibody followed by treatment with DAB reagent (Dako Omnis). IHC staining evaluation was performed using Halo software. The expression of YEATS4 and KAT8 were evaluated based on the H‐score incorporating both the staining intensity (0, no evidence of staining; 1, weak; 2, moderate; 3, strong) and the percentage of stained cells at each intensity level (0% to 100%). The final H‐score was calculated by multiplying the staining intensity by the percentage of cells as previously reported.^[^ ^23^, ^24^ ^]^ The IHC cutoff for high or low expression of YEATS4 and KAT8 was determined separately based on their H‐scores.

### YEATS4 3KR Knock‐in UMUC3 Cells

To generate YEATS4 K64,65,69R (3KR), which was located at exon 3, an optimal sgRNA closest to the genomic target site was selected, cloned, and inserted into the lentiCRISPRv2 vector. A 2 kb homologous recombination (HR) donor template containing sequences encoding the K64,65,69R substitution as well as the remaining exon 4–7 of YEATS4 was designed, cloned, and inserted into the PUC19 vector. Additionally, to improve the efficiency of positive clone selection, sequences encoding the internal ribosome entry site and EGFP were placed behind exon 7 mentioned above.

sgRNA‐containing lentiCRISPRv2 and HR donor template constructs were cotransfected into UMUC3 cells. Seven days after transfection, GFP‐positive cells were sorted by flow cytometry and single cells were seeded into each well of 96‐well plates. Ten days later, single‐cell clones were picked, and site‐specific PCR was performed. The sequences of the gene‐edited clones were validated by Sanger sequencing. The sgRNA sequence is listed in Table S3 (Supporting Information).

### Apoptosis Assay

The apoptotic cells were obtained and subjected to apoptosis assay using an Annexin V‐FITC/PI Apoptosis Detection Kit (KGA107, KeyGEN BioTECH) followed by flow cytometry.

### Animal Experiments

4 ‐ to five‐week‐old female nude mice used in this study were purchased from Vital River Laboratories (Beijing, China). ≈2–3 × 10^6^ UMUC3 or T24 cells expressing nontargeted control sgRNA (sgRNA) or YEATS4‐targeting sgRNA were suspended in PBS and mixed with Matrigel at a ratio of 1:1. Cells were injected subcutaneously into the posterior flanks of the nude mice (*n* = 6‐8 per group). Seven days after injection, tumors were measured every 3 days using a Vernier, and mice were sacrificed at the final time point. For MG149 treatment experiments, mice bearing UMUC3 tumors were randomly divided into 2 groups (*n* = 6 per group) and were injected intraperitoneally with DMSO or 5 mg kg^−1^ MG149 every other day. For DDP treatment experiments, mice bearing parental UMUC3 (hereafter termed WT cells) or YEATS4 3KR knock‐in UMUC3 (hereafter termed 3KR cells) tumors were randomly divided into 2 groups (n = 6 per group) and were injected intraperitoneally with PBS or 2 mg kg^−1^ DDP every 3 days. For DDP and MG149 combination treatment assays, mice bearing T24 tumors were randomly divided into 4 groups (*n* = 8 per group): vehicle, DDP (2 mg kg^−1^, every 4 days), MG149 (5 mg kg^−1^, every other day), and DDP plus MG149. Drugs were injected intraperitoneally into nude mice.

### RNA‐Sequencing Analysis

T24 cells stably expressing sgRNAs targeting YEATS4 or the control were collected, and total RNA was extracted using TRIzol reagent (Life Technologies, 15596026). RNA‐sequencing assays were performed by Jiayin Biotechonology Ltd. (Shanghai, China) using an Illumina NovaSeq 6000. For data analysis, after quality control, STAR was used to align clean reads to the human genome GRCh 38 (Hg38). The DESeq2 algorithm was applied to filter the differentially expressed genes with the following criteria: |log2 fold change| > 1, P value < 0.05.

### CRISPR‒Cas9 Screening for the YEATS4 Protein Stability Regulator Screening Assay (ProSRSA)

This procedure has been previously described.^[^ ^16a^ ^]^ Briefly, 2 × 10^7 UMUC3 cells stably expressing psin‐DsRed‐P2A‐EGFP‐YEATS4 were infected with the lenti‐CRISPR library targeting ubiquitin family genes at 0.3 MOI. Forty‐eight hours after transfection, the DMEM medium was discarded and replaced with medium supplemented with 0.5 µg/ml puromycin for selection. One week later, the cells were collected and subjected to flow cytometry sorting by using a Beckman MoFlo Cell Sorting System. Cells residing within the top 5% of the EGFP/DsRed ratio (hereafter named EGFP/DsRed^high^ cells) and the bottom 5% of the EGFP/DsRed ratio (hereafter named EGFP/DsRed^low^ cells) were sorted, respectively.

### sgRNA PCR Amplification and High‐Throughput Sequencing

Genomic DNA was extracted from EGFP/DsRed^high^ or EGFP/DsRed^low^ cells using a TIANamp genomic DNA Kit (Tiangen, Beijing, China). PCR amplification was performed using the following primers: F: tcttgtggaaaggacgaaacaccg, R: cttctctaggcaccggatcaattgc. The amplicons were purified and sequenced with 150‐base pair paired‐end sequencing strategies using Illumina NovaSeq.

### Statistical Analysis

All data in this study were presented as the mean ± SD from 3 independent experiments. GraphPad Prism (Version 9.0) was used for all statistical analyses. The error bars indicated the SD. Two‐tailed Student's *t*‐test were used to compare the statistical significance of differences between the 2 groups. YEATS4 or KAT8 expression and overall survival curves were assessed by Kaplan‒Meier plots and compared using a log‐rank test. For correlation analysis, the Spearman correlation coefficient was calculated for crosstab using Spearman's correlation test, and *p*‐value was calculated using Pearson's chi‐squared test. Differences were considered statistically significant when *p*‐values were < 0.05.

## Conflict of Interest

The authors declare no conflict of interest.

## Author Contributions

M.X., L.Z., and T.L. contributed equally to this work. M.X., Y.W., and T.K. conceived and designed the project. M.X. performed most of the experiments. L.Z. performed all bioinformatics. T.L. helped with the animal experiments. Y.L., X.Z., C.Z., L.Zhong, and Y.Z. helped with biochemistry experiments. X.H. helped with the plasmid clone. X.Z. and L.Z. helped to collect clinical samples. R.Z. provided technical assistance. M.X., Y.W., and T.K. wrote the manuscript, and the other authors helped to revise and proofread the manuscript.

## Supporting information

Supporting Information

Supporting Information

Supporting Information

## Data Availability

RNA‐sequencing data underlying this article have been deposited in the Genome Sequence Archive for Humans (PRJCA023499). Mass spectrometry data have been deposited in the ProteomeXchange Consortium via the iProX partner repository^[^ ^36^ ^]^with the accession codes PXD047300 and PXD047355. All the remaining data are available in the main text or the supplemental materials.
