# Correction to “Targeting the KAT8/YEATS4 Axis Represses Tumor Growth and Increases Cisplatin Sensitivity in Bladder Cancer”

**DOI:** 10.1002/advs.202513864

**Published:** 2025-08-04

Xie M, Zhou L, Li T, et al. Targeting the KAT8/YEATS4 Axis Represses Tumor Growth and Increases Cisplatin Sensitivity in Bladder Cancer. Adv Sci (Weinh), 2024. 11(22): p. e2310146.

In Figure 6L, the immunohistochemical image of YEATS4 in Case 1 was inadvertently duplicated with the image of KAT8.

The YEATS4 image in Case 1 should be corrected, therefore the Figure 6 should be revised to the following revised version.


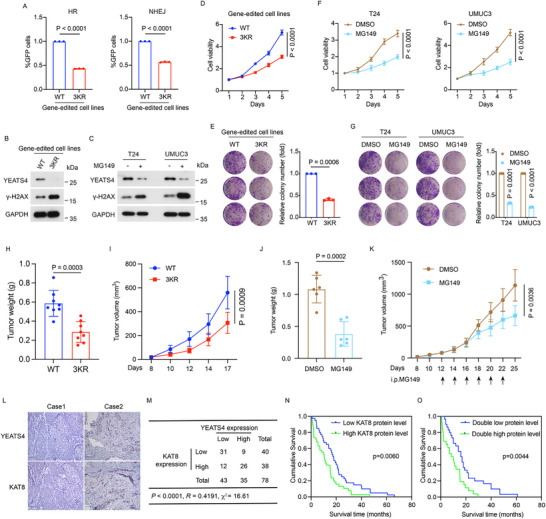


Figure 6. Acetylation of YEATS4 by KAT8 is critical for its oncogenic function in bladder cancer cell viability. A) Relative DNA repair efficiency in YEATS4 WT and 3KR cells. B) Western blotting of the indicated proteins in YEATS4 WT and 3KR cells. *n *= 3 biologically independent experiments. C) T24 and UMUC3 cells were treated with or without 50 µM MG149 for 48 h and then were lysed and analyzed by Western blotting. *n *= 3 biologically independent experiments. D and E) MTT assays D) and colony formation assays E) were performed in YEATS4 WT and 3KR cells. F,G) MTT assay (F) and colony formation assay (G) were performed in T24 and UMUC3 cells treated with DMSO or 50 µM MG149. H,I) Tumor growth of YEATS4 WT and 3KR cells was evaluated in vivo. *n *= 8 nude mice per group. Tumor weights (H) and tumor volumes (I) were measured. J,K) Mice bearing UMUC3 tumors were randomly divided into the indicated groups (*n *= 6 mice per group). DMSO or MG149 (5 mg kg^−1^) was injected intraperitoneally at the indicated time points. Tumor weights (J) and tumor volumes (K) were measured. L) Representative immunohistochemical staining images of both YEATS4 and KAT8 in 78 paraffin‐embedded BC tissues. Scale bar, 100 µm. M) Crosstab shows the distribution of cancer tissues in the 78 bladder cancer tissues used in (L) according to the H‐Score of YEATS4 and KAT8. *p <* 0.0001, *𝜒*2 tests. *R*, Spearman correlation coefficient. *p* values were analyzed using Pearson's chi‐squared test, and the *R*‐value was analyzed using Spearman's correlation test. N,O) Overall survival curves were generated based on the protein levels of YEATS4 or KAT8 in the BC tissues used in (L). *p* value was calculated using the Kaplan‒Meier plots. Data in A and D–K are presented as the mean ± SD. *p* values were calculated by a two‐tailed Student's *t*‐test.

We apologize for this error.

