# Supplementary material for: Targeting the KAT8/YEATS4 Axis Represses Tumor Growth and Increases Cisplatin Sensitivity in Bladder Cancer
Source: Adv Sci (Weinh). 2024 Mar 25;11(22):2310146. doi: 10.1002/advs.202310146 (PMC11165526; doi:10.1002/advs.202310146)
Supplement: Supplementary file 1 — Supporting Information [file ADVS-11-2310146-s003.pdf]

ADVANCED SCIENCE
Open Access

Supporting Information

for *Adv. Sci.*, DOI 10.1002/advs.202310146

Targeting the KAT8/YEATS4 Axis Represses Tumor Growth and Increases Cisplatin Sensitivity in Bladder Cancer

Miner Xie, Liwen Zhou, Ting Li, Yujie Lin, Ruhua Zhang, Xianchong Zheng, Cuiling Zeng, Lisi Zheng, Li Zhong, Xiaodan Huang, Yezi Zou, Tiebang Kang and Yuanzhong Wu**

Supplemental figures

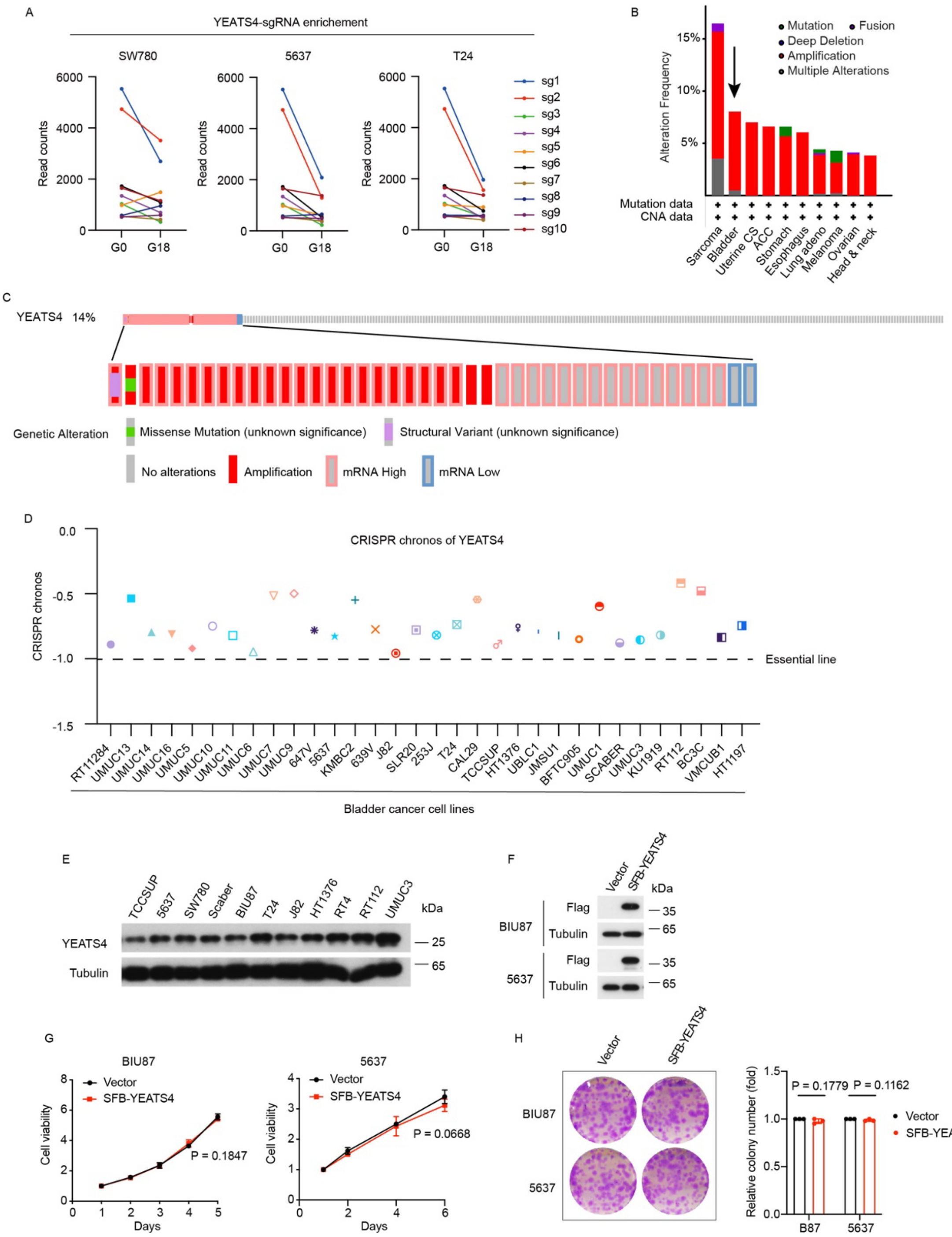

Figure S1. Overexpression of YEATS4 does not affect the proliferation of bladder cancer cells. (A) Read counts of individual sgRNAs targeting YEATS4 are shown**. (B)** YEATS4 is amplified in various of human cancers. The histogram indicates the alteration frequency of YEATS4. Data were acquired from cBioPortal. **(C)** Genetic alterations of YEATS4 in bladder cancer from TCGA. **(D)** YEATS4 is required for the

growth of bladder cancer cell lines. Dots indicate the CRISPR Chronos Score of each cell line. Data were acquired from the DepMap portal. The Chronos score (Y axis) is based on data from a cell depletion assay. A lower Chronos score indicates a higher likelihood that the gene of interest is essential in a given cell line. A score of 0 indicates a gene is not essential whereas a score of -1 corresponds to the median of pan-essential genes. **(E)** YEATS4 protein levels were analyzed in the indicated cell lines by Western blotting from one independent experiment. **(F)** Western blotting of the indicated proteins in the indicated stable cells. n = 3 independent experiments. **(G)** Cell viability of the indicated stable cells was evaluated by MTT assay. n = 3 independent experiments. (**H**) Colony formation assays were performed for the indicated stable cells. Colony numbers were quantified using ImageJ software. n = 3 independent experiments. Data in G and H are presented as the mean ± SD of three independent experiments. *P* values were calculated by two-tailed Student's *t* test.

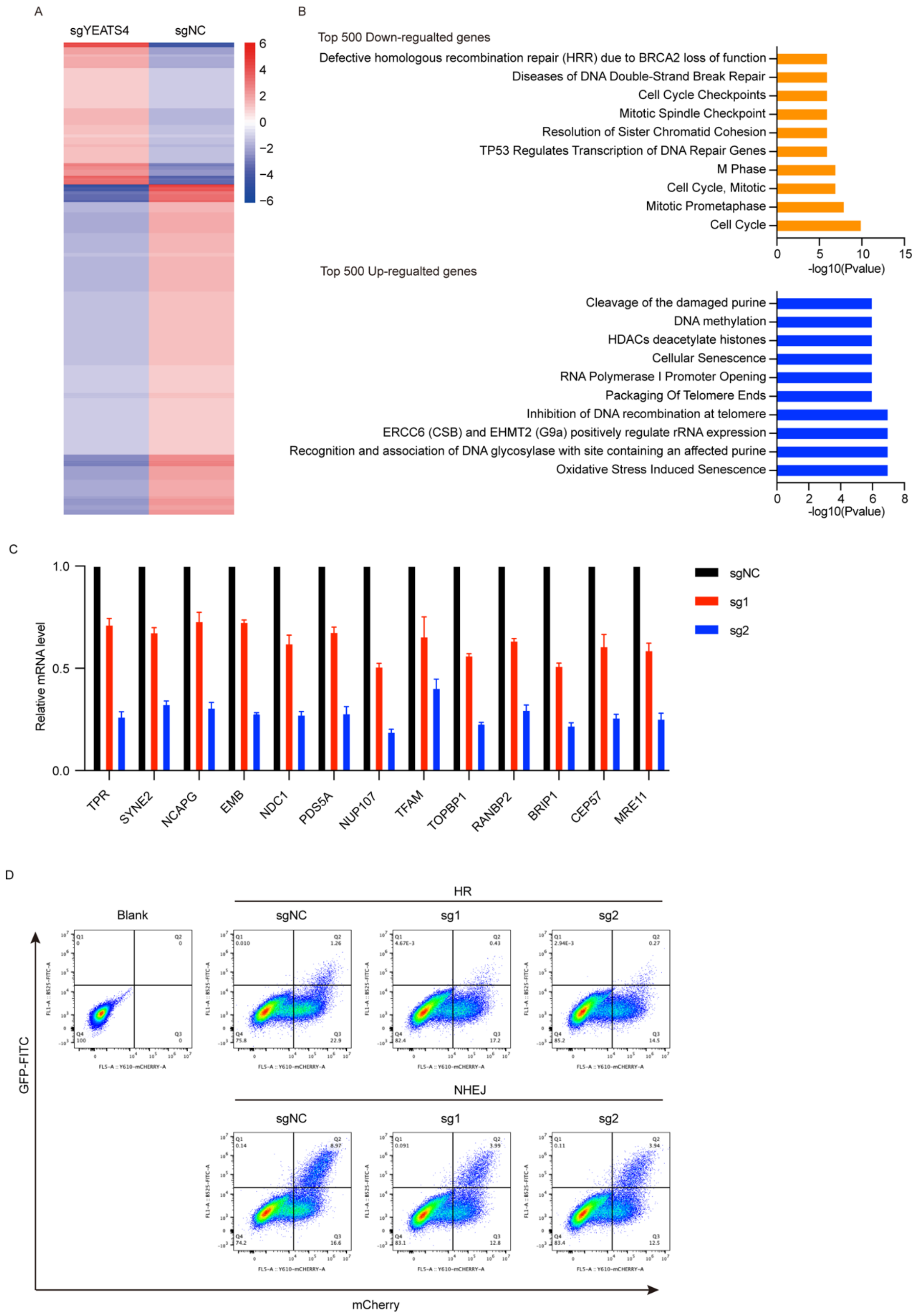

Figure S2. YEATS4 regulates the expression of DNA repair and cell cycle-related genes. **(A)** Heatmap representation of differentially expressed genes in the indicated T24 stable cells (with a cutoff of $\log_2$fold change ≥ 1, and false discovery rate (FDR) <

0.05). Red and blue indicate up and down genes, respectively. See Table S1. **(B)** Reactome analysis of the top 500 upregulated and 500 downregulated genes. **(C)** qRT-PCR analysis of selected downregulated genes in YEATS4 knockout T24 cells. The relative mRNA levels of the indicated genes were normalized to the GAPDH levels. Data are presented as the mean ± SD of three independent experiments. **(D)** Flow cytometry analysis of the HR or NHEJ repair efficiency of UMUC3 stable cells transfected with YEATS4-targeted sgRNAs.

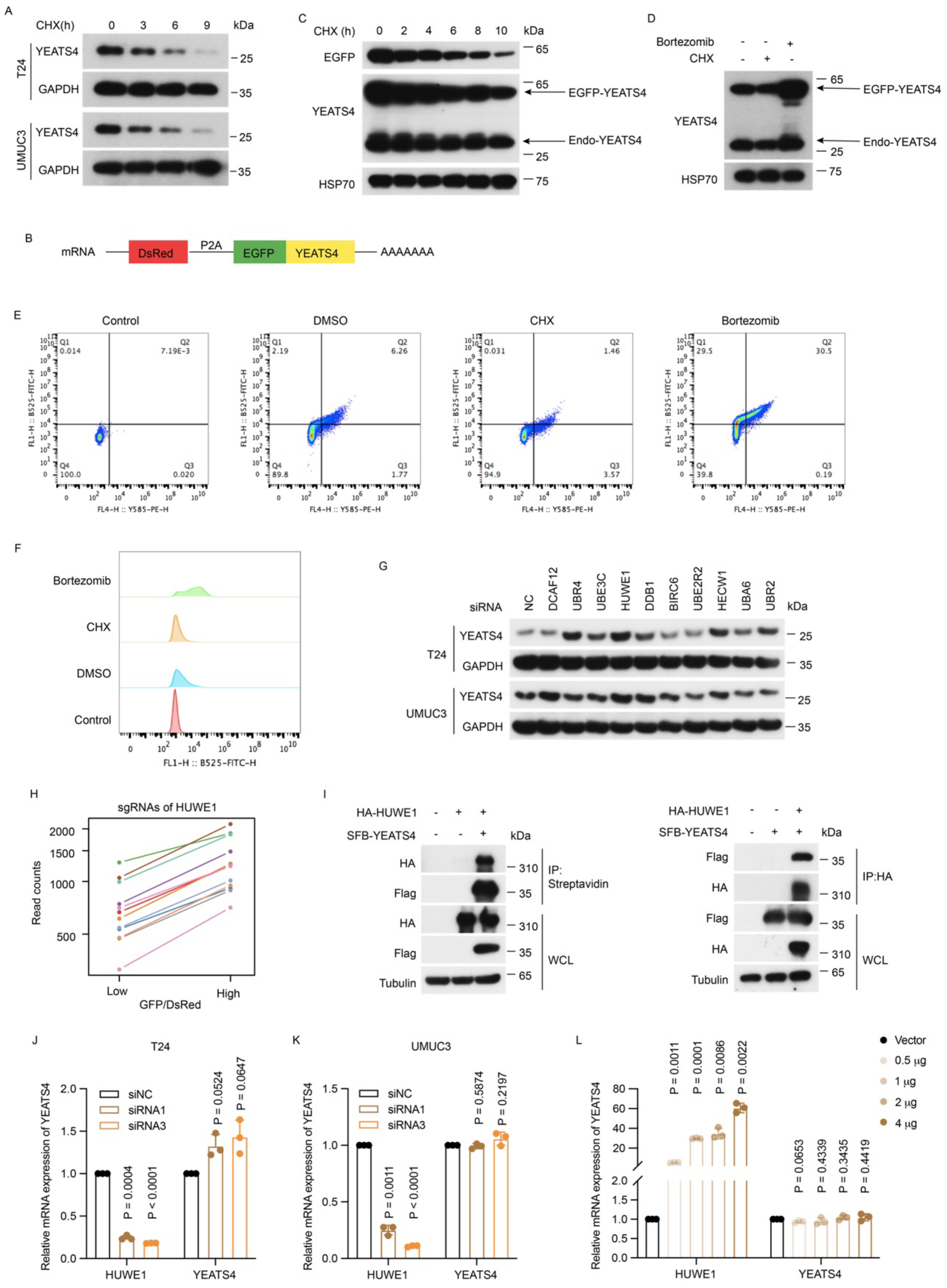

Figure S3. YEATS4 protein stability is regulated by HUWE1. (A) The indicated cell lines were treated with 40 μg/ml CHX at the indicated time points and then were lysed and analyzed by Western blotting. **(B)** The DsRed-P2A-EGFP-YEATS4 element was cloned and inserted into a lentiviral vector. **(C)** HEK293T cells transiently transfected with the plasmid constructed in **(A)** for 48 h were treated with 40 μg/ml

CHX at the indicated time points, then lysed and analyzed by Western blotting. **(D-F)** The indicated stable cells infected with psin-DsRed-P2A-EGFP-YEATS4 lentivirus were treated with DMSO, 40 μg/ml CHX or 1 μM bortezomib for 8 h, and then subjected to Western blotting **(D)** and flow cytometry **(E and F)**. **(G)** T24 and UMUC3 cells transfected with the indicated siRNAs for 48 h were analyzed by Western blotting. **(H)** Read counts of individual sgRNAs targeting HUWE1 in low and high samples are shown. **(I)** HEK293T cells were cotransfected with the indicated plasmids for 48 h, and then subjected to IP using streptavidin beads (left panel) or HA beads (right panel) followed by Western blotting. **(J-L)** qRT–PCR analysis of YEATS4 in T24 and UMUC3 cells expressing HUWE1-targeted siRNAs **(J and K)** and in HEK293T cells transfected with vector or HUWE1 plasmid at the indicated concentrations **(L)**. The relative mRNA levels of YEATS4 were normalized to those of GAPDH. Data are presented as the mean ± SD of n = 3 independent experiments. *P* values were analyzed using the two-tailed Student's *t*-test.

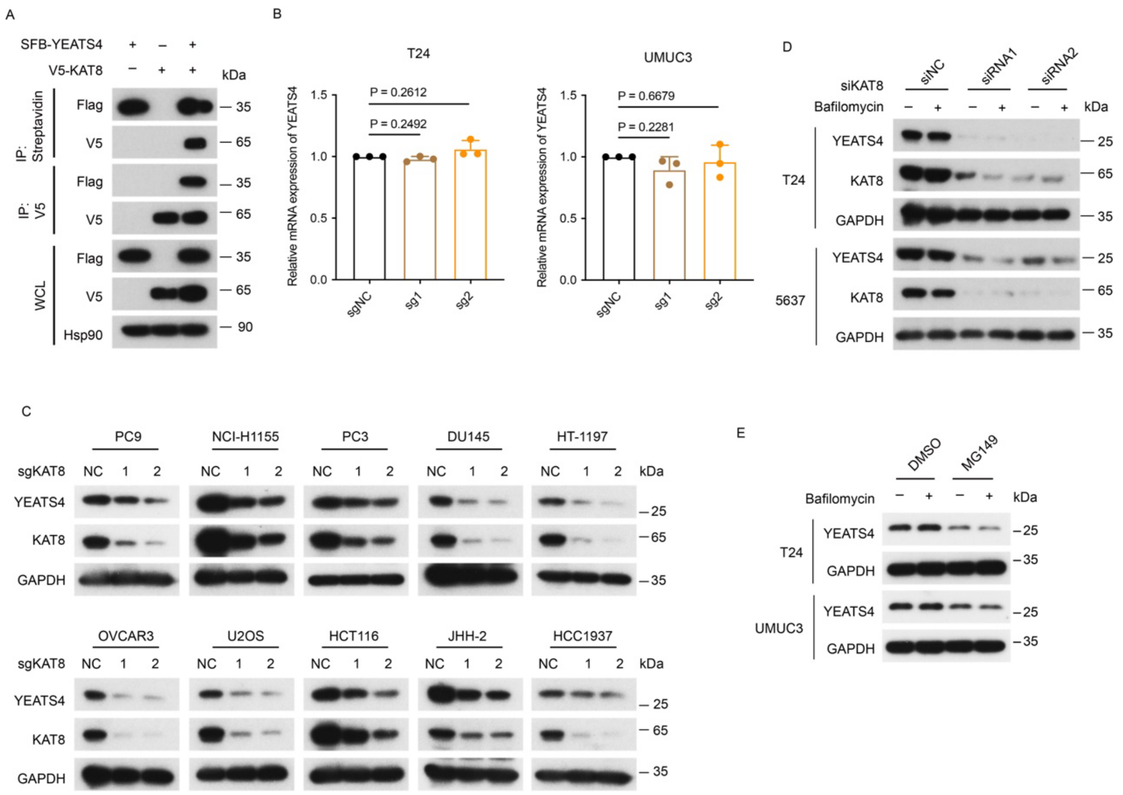

Figure S4. Knockout of KAT8 decreases the expression of YEATS4. (A) HEK293T cells were cotransfected with the indicated constructs for 48 h, and then subjected to IP

using V5-agarose beads or streptavidin beads followed by Western blotting. **(B)** The relative mRNA levels of YEATS4 in the indicated stable cells were normalized to the GAPDH levels as determined by qRT-PCR. Data are presented as the mean ± SD. of n = 3 independent experiments. *P* values were analyzed using the two-tailed Student's *t*-test. **(C)** Western blotting of YEATS4 expression in the indicated stable cells transfected with KAT8-targeted sgRNAs. **(D and E)** The indicated cells transfected with KAT8-targeted siRNAs for 48 h **(D)** or incubated with or without 50 μM MG149 for 48 h **(E)** were treated with or without 200 nM bafilomycin for 6 h, and then were lysed and analyzed by Western blotting.

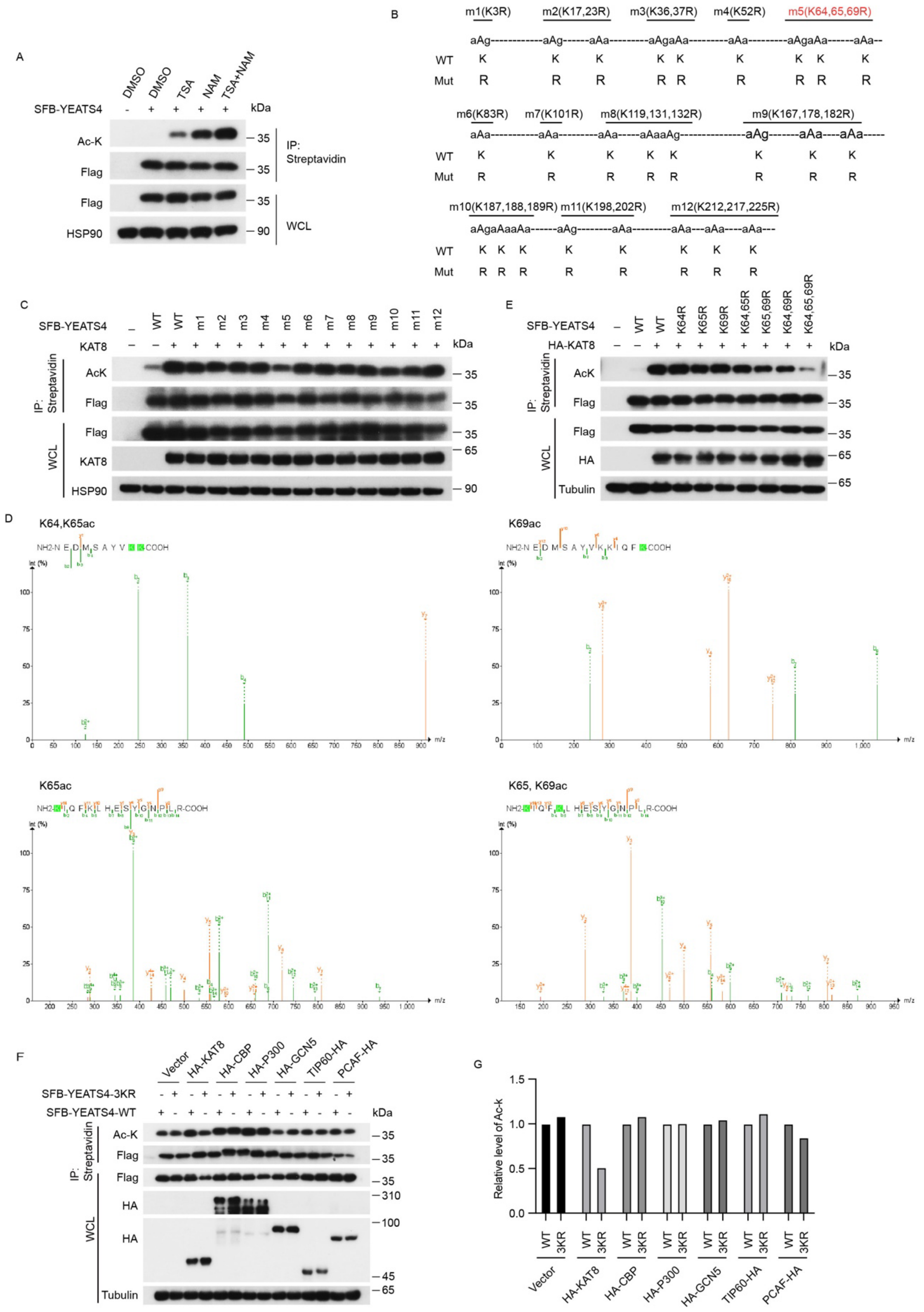

Figure S5. YEATS4 is acetylated by KAT8 at K64, 65, and 69. (**A**) HEK293T cells transiently transfected with SFB-YEATS4 plasmids for 24 h were treated with DMSO, 5 µM TSA, 5 mM NAM, or both compounds for another 24 h. Then the cells were subjected to IP using streptavidin beads and analyzed by Western blotting. **(B)** The

schematics of YEATS4 WT and its twelve mutants (presented as m1-m12). The Lys residues (K) in YEATS4 were replaced by Arg (R) (K-R) at the indicated positions. **(C)** HEK293T cells were transfected with SFB-YEATS4-WT or its mutants as indicated in **(B)** for 48 h, then subjected to IP using streptavidin beads and analyzed by Western blotting. **(D)** Acetylation at K64, 65, K65, K69, and K65, 69 was identified by mass spectrometry. **(E)** K64R, K65R, K69R, K64, 65R, K65, 69R, K64, 69R, and K64, 65, 69R mutants were constructed and transfected into HEK293T cells for 48 h. Then the cells were subjected to IP using streptavidin beads and analyzed by Western blotting. **(F and G)** SFB-YEATS4-WT or SFB-YEATS4-3KR mutant and the indicated acetyltransferases were cotransfected into HEK293T cells for 48 h, and then the cells were subjected to IP using streptavidin beads followed by Western blotting **(F).** The relative level of acetylation (shown as Ac-k) was normalized to Flag based on the Western blotting results **(G)**.

A

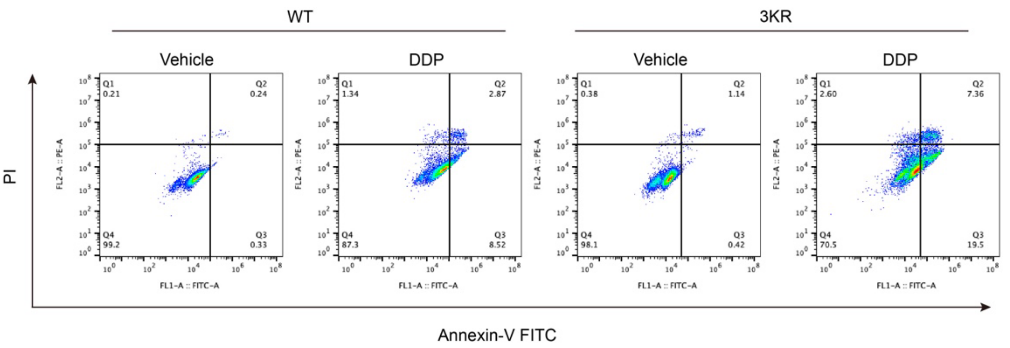

B

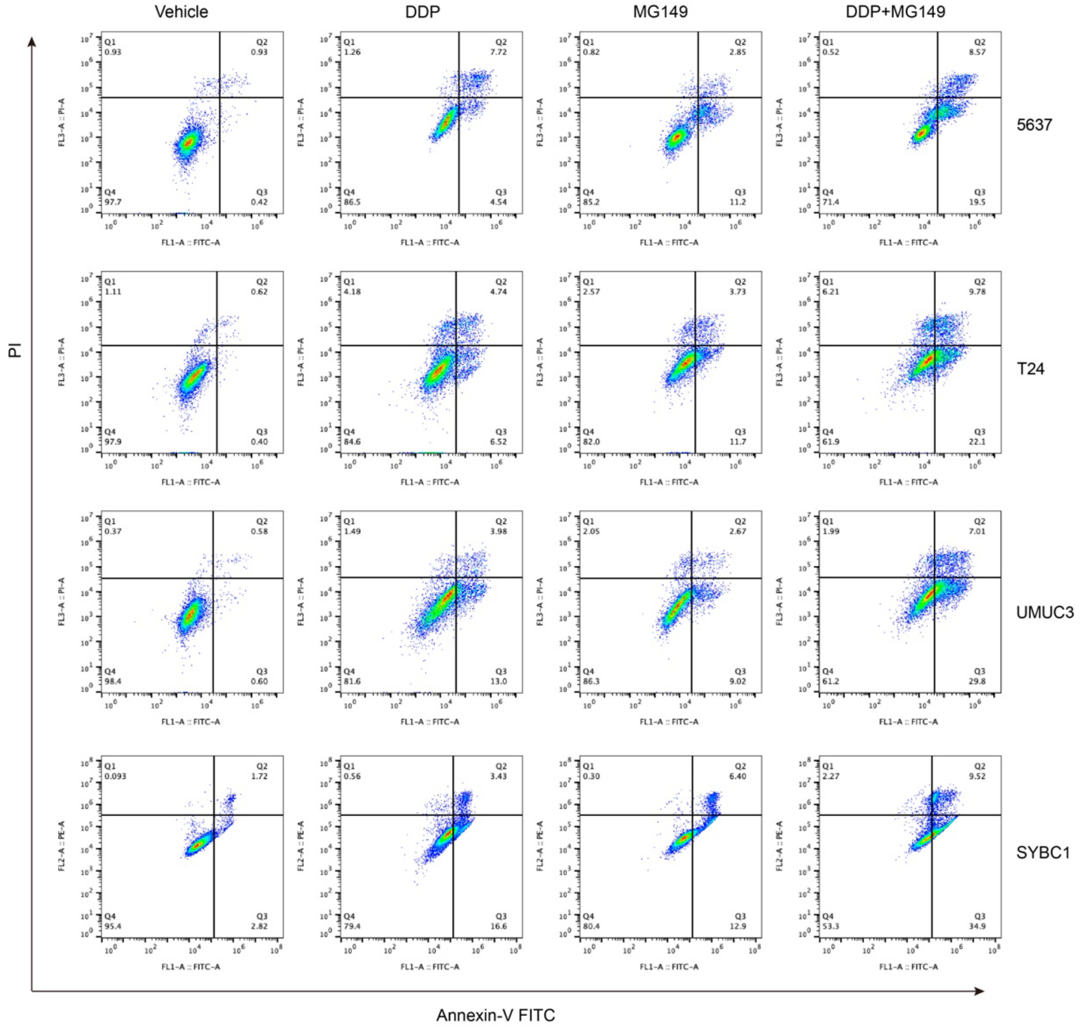

C

Combination group	DDP Concentration (μM)	MG149 Concentration (μM)
1	0	0
2	0.315	1.25
3	0.625	2.5
4	1.25	5
5	2.5	10
6	5	20
7	10	40
8	20	80
9	40	160

Figure S6. Defects in YEATS4 acetylation increase cell apoptosis upon DDP treatment. (A) Flow cytometry analysis of apoptosis in parental WT or 3KR cells treated with 5 μM DDP for 48 h. **(B)** Flow cytometry analysis of apoptosis in the indicated cells treated with 2.5 μM DDP, 50 μM MG149, and their combination for 48 h. **(C)** The concentrations of DDP and MG149 used in MTT assays, respectively.

Supplementary Tables

Table S1. Differential expression genes in RNA-seq analysis.

Table S2. Proteins identified by mass spectrometry in proximity labeling assay.

(Table S1 and S2 are independent Excel files)

Table S3. Target sequences of sgRNAs and siRNAs.

sgRNA or siRNA	Nucleotide sequence
YEATS4 sgRNA1	AAGATGGGCACACTCATCAG
YEATS4 sgRNA2	GTTTCAGTAATTTCATATGG
KAT8 sgRNA1	AGCCTTGGAGAAGGAGCATG
KAT8 sgRNA2	CTTGTCCACATACTTCACCT
NC sgRNA	TTCTCCGAACGTGTCACGTA
KAT8 siRNA1	GCAAGATCACTCGCAACCA
KAT8 siRNA2	GGAATTCTATGTACACTAC
HUWE1 siRNA1	GCTCAATACTAGCCGTCTA
HUWE1 siRNA2	CAGTGTTGCTCCTGATTGA
YEATS4 3KR KI sgRNA	AGCTATGGCAATCCTTTAAG

Table S4. Primers used in qRT-PCR.

**qRT-PCR Primers**		
**Gene**	**Forward sequence (5'-3')**	**Reverse sequence (5'-3')**
YEATS4	TACTGAAACAGGATGGGGTGAA	TTGCATCATTGCTGTTGGGTC
HUWE1	TTGGACCGCTTCGATGGAATA	TGAAGTTCAACACAGCCAAGAG
GAPDH	ACAGTCAGCCGCATCTTCTT	GACAAGCTTCCCGTTCTCAG
TPR	AAGGGGTAACCACGTAATTCGT	GGCCACTAAGAGACGTTGATTT

SYNE2	GTGGTCTCTGTCAACGTGAGC	GAGCGACTGTCGTAAGCCC
NCAPG	GAGGCTGCTGTCGATTAAGGA	AACTGTCTTATCATCCATCGTGC
EMB	ATGGGGAATCTTACTGGTGCC	CCCCTCATCTGAGTGCTTCTTT
NDC1	GGGCCAGTACAGCTTTCTTGT	CCATAAATGCTCCAGTCAGTAGG
PDS5A	GAACAGTCCAGACTATTGAGGC	GCTGTGGCATGACGGATAATAA
NUP107	AGTGCTGTTATGTTACGTGAGG	GTACTCGAAGAGTGCTTCAGAAA
TFAM	ATGGCGTTTCTCCGAAGCAT	TCCGCCCTATAAGCATCTTGA
TOPBP1	TTCAGCAACTCACAGTTAAGCA	GGCACACTCATACTTCTGACC
RANBP2	AAACCTCCGATTGCAGCTCAT	GGCAAAGATGGCCTTAATCCT
BRIP1	GGAAACAGTCAAGAGTCATCGAA	TCTGAGCAATCTGCTTGTGTG
CEP57	GTGGTAGCCAATGTTCAGCTT	AGGGAGGTGTTACTGACAACTT
MRE11	GGGGCAGATGCACTTTGTG	GAAGCAAAACCGGACTAATGTCT
