# Supplementary material for: Targeting the KAT8/YEATS4 Axis Represses Tumor Growth and Increases Cisplatin Sensitivity in Bladder Cancer
Source: Adv Sci (Weinh). 2024 Mar 25;11(22):2310146. doi: 10.1002/advs.202310146 (PMC11165526; doi:10.1002/advs.202310146)
Supplement: Supplementary file 2 — Supporting Information [file ADVS-11-2310146-s002.pdf]

ADVANCED SCIENCE
Open Access

Supporting Information

for *Adv. Sci.*, DOI 10.1002/advs.202310146

Targeting the KAT8/YEATS4 Axis Represses Tumor Growth and Increases Cisplatin Sensitivity in Bladder Cancer

Miner Xie, Liwen Zhou, Ting Li, Yujie Lin, Ruhua Zhang, Xianchong Zheng, Cuiling Zeng, Lisi Zheng, Li Zhong, Xiaodan Huang, Yezi Zou, Tiebang Kang and Yuanzhong Wu**

AccID	log2FC	Pvalue	FDR	Style
LRRIQ1	-6.103935721	7.14E-10	1.54E-09	down
AC092835.1	-5.634450438	1.57E-07	3.07E-07	down
KLHL4	-5.255938814	9.21E-11	2.05E-10	down
CCDC102B	-5.103935721	1.47E-05	2.60E-05	down
TRIM6	-5.103935721	1.47E-05	2.60E-05	down
GSAP	-5.063293736	1.57E-09	3.35E-09	down
CCDC121	-5.042535176	2.41E-17	6.57E-17	down
TNFSF15	-5.021473561	2.78E-09	5.87E-09	down
ENPEP	-5.00009991	7.48E-17	2.01E-16	down
ADAMTS3	-4.791991715	5.01E-08	9.99E-08	down
SLC26A7	-4.741365641	0.00015654	0.000261	down
WNT5A	-4.741365641	8.97E-08	1.78E-07	down
BCHE	-4.634450438	0.0002863	0.00046846	down
LRP1B	-4.634450438	0.0002863	0.00046846	down
GPR162	-4.51897322	0.00052644	0.00084805	down
CELF4	-4.393442338	0.00097344	0.00153944	down
NPHP3-ACAI	-4.393442338	0.00097344	0.00153944	down
GPRASP1	-4.326328142	5.63E-06	1.02E-05	down
ARL13A	-4.255938814	0.00181069	0.00280814	down
CBLN3	-4.255938814	0.00181069	0.00280814	down
KCNH8	-4.255938814	0.00181069	0.00280814	down
LRRC17	-4.255938814	0.00181069	0.00280814	down
LRRC19	-4.255938814	0.00181069	0.00280814	down
NLRC3	-4.255938814	0.00181069	0.00280814	down
NPY1R	-4.255938814	1.02E-05	1.83E-05	down
ANKRD20A1	-4.103935721	0.00338937	0.00513226	down
FHIT	-4.103935721	0.00338937	0.00513226	down
KLB	-4.103935721	0.00338937	0.00513226	down
PDE11A	-4.103935721	0.00338937	0.00513226	down
REP15	-4.103935721	3.42E-05	5.92E-05	down
RSPH4A	-4.103935721	3.42E-05	5.92E-05	down
KLRG1	-4.021473561	6.26E-05	0.00010686	down
BDH2	-3.963758063	1.33E-11	3.04E-11	down
CLHC1	-3.956378532	6.82E-15	1.74E-14	down
C1orf146	-3.934010719	0.00638733	0.00943105	down
CXXC4	-3.934010719	0.00638733	0.00943105	down
DLEC1	-3.934010719	0.00638733	0.00943105	down
EPS8L1	-3.934010719	0.00638733	0.00943105	down
PCDHGA11	-3.934010719	0.00638733	0.00943105	down
PP2D1	-3.934010719	0.00638733	0.00943105	down
CACNB2	-3.840901315	0.00021114	0.00034881	down
JPH3	-3.840901315	0.00021114	0.00034881	down
MAF	-3.840901315	0.00021114	0.00034881	down
CDR1	-3.782007626	6.72E-16	1.76E-15	down
MNS1	-3.782007626	6.72E-16	1.76E-15	down
WDR72	-3.756740867	1.97E-57	1.22E-56	down
ANGPTL1	-3.741365641	0.01212443	0.01743173	down
COL4A4	-3.741365641	0.01212443	0.01743173	down
CRYBG1	-3.741365641	0.01212443	0.01743173	down
FSBP	-3.741365641	0.01212443	0.01743173	down
GBP1	-3.741365641	0.01212443	0.01743173	down
GNRH1	-3.741365641	5.25E-07	1.00E-06	down
MAGEA6	-3.741365641	0.01212443	0.01743173	down

MAP2K6	-3.741365641	0.01212443	0.01743173	down
NLRC4	-3.741365641	0.01212443	0.01743173	down
NOTCH2NLA	-3.741365641	0.00038882	0.0006307	down
OR1F1	-3.741365641	0.01212443	0.01743173	down
RASA4	-3.741365641	0.01212443	0.01743173	down
RORB	-3.741365641	0.01212443	0.01743173	down
TTC23L	-3.741365641	0.01212443	0.01743173	down
TTPA	-3.741365641	0.00038882	0.0006307	down
ABHD12B	-3.634450438	0.00071719	0.00114449	down
GHR	-3.634450438	0.00071719	0.00114449	down
GPM6A	-3.634450438	0.00071719	0.00114449	down
MAGI2	-3.634450438	0.00071719	0.00114449	down
N4BP2L1	-3.634450438	0.00071719	0.00114449	down
TCEAL8	-3.596975732	8.34E-09	1.73E-08	down
PABPC4L	-3.592222202	6.10E-21	1.81E-20	down
LRRIQ3	-3.577866909	3.11E-06	5.70E-06	down
COL4A3	-3.51897322	0.02319563	0.03225107	down
FAM47E	-3.51897322	0.02319563	0.03225107	down
NFIA	-3.51897322	0.02319563	0.03225107	down
NTF4	-3.51897322	0.02319563	0.03225107	down
PCDHB9	-3.51897322	0.02319563	0.03225107	down
RHCE	-3.51897322	0.02319563	0.03225107	down
SAMD13	-3.51897322	0.02319563	0.03225107	down
SLCO2B1	-3.51897322	0.02319563	0.03225107	down
SMC1B	-3.51897322	0.00132484	0.00207369	down
SMIM30	-3.476744985	5.16E-58	3.21E-57	down
TRIM46	-3.43651106	8.63E-08	1.71E-07	down
F2R	-3.393442338	0.00245044	0.00375396	down
IFNLR1	-3.393442338	0.00245044	0.00375396	down
MAML2	-3.393442338	1.83E-05	3.23E-05	down
MDH1B	-3.393442338	1.83E-05	3.23E-05	down
MFSD4A	-3.393442338	0.00245044	0.00375396	down
DDX60	-3.349048219	5.56E-19	1.57E-18	down
PIH1D2	-3.349048219	2.77E-07	5.35E-07	down
SYCP3	-3.326328142	3.31E-05	5.74E-05	down
TNFSF10	-3.326328142	3.31E-05	5.74E-05	down
ZNF284	-3.312522343	7.02E-11	1.57E-10	down
NAT1	-3.303244529	4.95E-07	9.45E-07	down
HNF4G	-3.279785556	2.03E-12	4.79E-12	down
CPS1	-3.276402917	3.37E-14	8.40E-14	down
ABCD2	-3.255938814	0.04475895	0.05993868	down
AC106886.5	-3.255938814	5.97E-05	0.00010207	down
AC112128.1	-3.255938814	0.04475895	0.05993868	down
AMY2B	-3.255938814	0.04475895	0.05993868	down
ASB14	-3.255938814	5.97E-05	0.00010207	down
B3GALT2	-3.255938814	0.04475895	0.05993868	down
BEST4	-3.255938814	0.00453682	0.00678396	down
CD226	-3.255938814	5.97E-05	0.00010207	down
CES3	-3.255938814	0.04475895	0.05993868	down
CRIP1	-3.255938814	0.04475895	0.05993868	down
ERVW-1	-3.255938814	0.04475895	0.05993868	down
GABRR2	-3.255938814	0.04475895	0.05993868	down
GPNMB	-3.255938814	0.00453682	0.00678396	down
GPRIN3	-3.255938814	0.04475895	0.05993868	down

IRF4	-3.255938814	0.04475895	0.05993868	down
KIAA2012	-3.255938814	0.04475895	0.05993868	down
LIPI	-3.255938814	0.00453682	0.00678396	down
LKAAEAR1	-3.255938814	0.04475895	0.05993868	down
MROH2A	-3.255938814	0.04475895	0.05993868	down
MYBPH	-3.255938814	0.04475895	0.05993868	down
MYO1H	-3.255938814	0.04475895	0.05993868	down
PCDHB11	-3.255938814	0.04475895	0.05993868	down
PI15	-3.255938814	0.04475895	0.05993868	down
PPP1R42	-3.255938814	0.04475895	0.05993868	down
RANGRF	-3.255938814	0.04475895	0.05993868	down
RARB	-3.255938814	0.04475895	0.05993868	down
RNF133	-3.255938814	0.04475895	0.05993868	down
SAMD9L	-3.255938814	0.04475895	0.05993868	down
SLAMF8	-3.255938814	0.04475895	0.05993868	down
SLC10A5	-3.255938814	0.00453682	0.00678396	down
SLC13A4	-3.255938814	0.00453682	0.00678396	down
SLC28A2	-3.255938814	0.04475895	0.05993868	down
SLC3A1	-3.255938814	0.04475895	0.05993868	down
TTYH2	-3.255938814	0.04475895	0.05993868	down
UNC5CL	-3.255938814	0.04475895	0.05993868	down
ZNF20	-3.255938814	0.04475895	0.05993868	down
GPX8	-3.225776844	2.81E-51	1.57E-50	down
MECOM	-3.210135125	1.19E-28	4.24E-28	down
ZNF223	-3.207029214	1.58E-06	2.94E-06	down
AC091551.1	-3.181938233	0.00010767	0.00018151	down
THBS2	-3.181938233	0.00010767	0.00018151	down
GGACT	-3.156403141	2.81E-06	5.17E-06	down
ZNF516	-3.156403141	2.81E-06	5.17E-06	down
IFT81	-3.149607579	5.38E-62	3.60E-61	down
E2F5	-3.147004443	4.33E-19	1.23E-18	down
PCDHB16	-3.103935721	0.00840471	0.01226981	down
QRICH2	-3.103935721	0.00840471	0.01226981	down
RGPD3	-3.103935721	5.00E-06	9.08E-06	down
SCUBE3	-3.103935721	0.0001938	0.00032111	down
ZNF418	-3.103935721	0.00840471	0.01226981	down
LRRCC1	-3.084952617	5.09E-27	1.74E-26	down
SENP7	-3.063293736	3.71E-54	2.16E-53	down
SAMD9	-3.049487937	3.33E-10	7.25E-10	down
IFT80	-3.03461466	2.18E-65	1.54E-64	down
CCDC191	-3.013737912	6.55E-17	1.76E-16	down
B3GNT5	-2.995786917	3.75E-56	2.27E-55	down
ETV1	-2.970352566	1.12E-66	8.08E-66	down
BCO2	-2.934010719	0.01557144	0.02210721	down
CASP1	-2.934010719	0.01557144	0.02210721	down
CCDC85A	-2.934010719	0.01557144	0.02210721	down
EGR4	-2.934010719	0.01557144	0.02210721	down
ERBB4	-2.934010719	3.97E-13	9.58E-13	down
GRIA4	-2.934010719	0.01557144	0.02210721	down
ISPD	-2.934010719	2.80E-05	4.87E-05	down
ITGB1BP2	-2.934010719	0.01557144	0.02210721	down
PCDHB14	-2.934010719	0.00062457	0.00100083	down
ZBED9	-2.934010719	0.01557144	0.02210721	down
ZNF396	-2.88820703	2.31E-06	4.26E-06	down

N4BP2	-2.870327429	1.76E-67	1.28E-66	down
FUT9	-2.856842859	8.26E-16	2.16E-15	down
SLC44A5	-2.848894106	8.56E-30	3.13E-29	down
KLF12	-2.844214049	3.40E-69	2.54E-68	down
CCDC125	-2.840901315	4.04E-06	7.38E-06	down
POTEF	-2.840901315	0.00111748	0.00175971	down
ZNF597	-2.840901315	4.04E-06	7.38E-06	down
ZNF727	-2.840901315	4.04E-06	7.38E-06	down
KLHL14	-2.838150704	3.29E-42	1.56E-41	down
SH3BGRL	-2.836785207	4.39E-271	2.17E-269	down
DSC2	-2.812147486	4.35E-142	7.85E-141	down
EEF1AKMT2	-2.799081139	2.46E-09	5.20E-09	down
CAPS2	-2.791991715	7.07E-06	1.27E-05	down
BTBD8	-2.772729812	1.10E-15	2.87E-15	down
HOOK1	-2.770789402	1.55E-113	2.07E-112	down
CEP290	-2.761628545	2.92E-109	3.69E-108	down
TTC33	-2.754032053	2.61E-87	2.47E-86	down
ZDHHC21	-2.746264441	9.76E-90	9.58E-89	down
ARID5B	-2.741365641	0.02883085	0.03967742	down
B3GAT2	-2.741365641	0.00199365	0.00308046	down
BOC	-2.741365641	0.02883085	0.03967742	down
CATSPERG	-2.741365641	0.02883085	0.03967742	down
CBLN2	-2.741365641	0.00015315	0.00025548	down
CLN3	-2.741365641	0.02883085	0.03967742	down
DENND2D	-2.741365641	0.02883085	0.03967742	down
DPP4	-2.741365641	0.00015315	0.00025548	down
GLIPR1L1	-2.741365641	0.02883085	0.03967742	down
NEK5	-2.741365641	0.02883085	0.03967742	down
NLRP11	-2.741365641	0.02883085	0.03967742	down
NOSTRIN	-2.741365641	0.02883085	0.03967742	down
PDE1C	-2.741365641	0.02883085	0.03967742	down
SEC16B	-2.741365641	0.00199365	0.00308046	down
SPOCK3	-2.741365641	0.02883085	0.03967742	down
TBR1	-2.741365641	0.02883085	0.03967742	down
TENM2	-2.741365641	0.00199365	0.00308046	down
UNC5C	-2.741365641	0.02883085	0.03967742	down
ITGA1	-2.710504678	5.72E-29	2.05E-28	down
BTC	-2.706600223	1.48E-07	2.91E-07	down
KCNT2	-2.699545466	1.77E-06	3.29E-06	down
L3MBTL1	-2.699545466	1.77E-06	3.29E-06	down
ATXN1	-2.688898222	2.15E-05	3.76E-05	down
KIAA0825	-2.688898222	2.15E-05	3.76E-05	down
MTMR11	-2.681244649	2.45E-15	6.31E-15	down
ZNF730	-2.678171815	3.53E-21	1.05E-20	down
PDE8B	-2.670976314	2.56E-07	4.96E-07	down
ZDHHC15	-2.670976314	0.00026846	0.00044048	down
C5orf49	-2.634450438	0.0035445	0.00535793	down
FBXO16	-2.634450438	3.72E-05	6.44E-05	down
LRGUK	-2.634450438	3.72E-05	6.44E-05	down
PAQR9	-2.634450438	0.0035445	0.00535793	down
TSHZ2	-2.634450438	0.0035445	0.00535793	down
VNN1	-2.634450438	0.0035445	0.00535793	down
ZFP2	-2.634450438	0.0035445	0.00535793	down
TBC1D8B	-2.631602075	7.87E-38	3.42E-37	down

AL096711.2	-2.625888424	1.37E-13	3.34E-13	down
ANKAR	-2.618508894	6.39E-08	1.27E-07	down
RIMS2	-2.617537055	8.46E-32	3.23E-31	down
DENND1B	-2.615188535	1.95E-54	1.15E-53	down
SPICE1	-2.612082625	5.27E-06	9.55E-06	down
RBM44	-2.596975732	7.55E-07	1.43E-06	down
TNFAIP6	-2.596975732	5.22E-15	1.33E-14	down
HDX	-2.592222202	7.85E-16	2.05E-15	down
COL4A5	-2.591988017	9.58E-56	5.74E-55	down
KBTBD3	-2.586087416	5.89E-14	1.46E-13	down
COL5A2	-2.577866909	6.44E-05	0.00010989	down
LRCH2	-2.577866909	1.59E-08	3.25E-08	down
INTS6L	-2.576217172	1.28E-62	8.70E-62	down
ERAP2	-2.574468333	2.00E-16	5.29E-16	down
STEAP1	-2.566278935	9.05E-06	1.62E-05	down
PPP1R9A	-2.565366126	1.43E-45	7.22E-45	down
CTAGE4	-2.558501584	1.29E-06	2.42E-06	down
TMC5	-2.558501584	1.29E-06	2.42E-06	down
C8orf44	-2.552920552	1.86E-07	3.64E-07	down
SLC18B1	-2.548720564	1.02E-28	3.64E-28	down
ALG10B	-2.54330255	1.12E-165	2.61E-164	down
YEATS4	-2.542606877	1.30E-94	1.37E-93	down
SMPDL3A	-2.540668291	8.64E-11	1.92E-10	down
LNX1	-2.534914764	4.26E-14	1.06E-13	down
TMTC3	-2.528905755	1.46E-160	3.22E-159	down
ATG4C	-2.523112951	2.96E-49	1.60E-48	down
DPY19L4	-2.52167237	4.84E-147	9.32E-146	down
C5	-2.51897322	3.17E-07	6.12E-07	down
DNAI1	-2.51897322	0.00627555	0.00927298	down
PLAC1	-2.51897322	0.00627555	0.00927298	down
PRKG2	-2.51897322	2.20E-06	4.07E-06	down
SCIMP	-2.51897322	0.00011105	0.00018697	down
SLC15A2	-2.51897322	0.00081615	0.00129608	down
THEG	-2.51897322	0.00627555	0.00927298	down
TMEM144	-2.51897322	6.64E-32	2.55E-31	down
ZNF112	-2.51897322	2.42E-16	6.41E-16	down
PPIP5K2	-2.517519623	2.24E-270	1.10E-268	down
ZBTB41	-2.515698088	5.75E-121	8.36E-120	down
ELOVL6	-2.51119587	8.91E-27	3.03E-26	down
HMGN5	-2.509512891	7.02E-43	3.37E-42	down
PDE5A	-2.504759361	2.71E-15	6.98E-15	down
SAMD12	-2.497911605	2.34E-38	1.03E-37	down
ZNF716	-2.494725674	3.03E-57	1.87E-56	down
DCLRE1A	-2.490060528	5.68E-68	4.16E-67	down
SMAD9	-2.488599571	7.80E-08	1.55E-07	down
RNFT1	-2.481498515	1.66E-52	9.43E-52	down
TCP11L2	-2.478331236	3.75E-06	6.86E-06	down
ZNF555	-2.478331236	9.52E-30	3.48E-29	down
FAM13A	-2.475904498	7.63E-15	1.94E-14	down
RBM43	-2.469142064	2.53E-43	1.22E-42	down
EXTL2	-2.464723595	7.54E-109	9.50E-108	down
MINDY2	-2.453055909	3.33E-117	4.67E-116	down
HOXA9	-2.448583892	3.82E-12	8.90E-12	down
ZNF711	-2.443024367	2.70E-50	1.49E-49	down

MARCKS	-2.441270863	2.73E-200	8.48E-199	down
AC073896.1	-2.43651106	0.00141416	0.00220821	down
LONRF1	-2.43651106	0.00141416	0.00220821	down
BBS10	-2.427550192	1.31E-29	4.77E-29	down
ZNF772	-2.425863816	6.91E-37	2.94E-36	down
BAZ2B	-2.421276546	6.77E-63	4.60E-62	down
DPY19L2	-2.419437547	4.51E-05	7.76E-05	down
PRTFDC1	-2.419437547	3.37E-16	8.89E-16	down
NHLRC3	-2.413701177	3.20E-34	1.29E-33	down
B4GALT6	-2.408414845	1.68E-45	8.47E-45	down
FPGT-TNNI3I	-2.393442338	0.0110552	0.01596918	down
PMCH	-2.393442338	0.0110552	0.01596918	down
TUBA3D	-2.393442338	0.0110552	0.01596918	down
ZNF19	-2.393442338	1.08E-05	1.92E-05	down
TLR6	-2.384672128	4.43E-12	1.03E-11	down
ZFHX4	-2.377743652	7.73E-60	4.97E-59	down
ZNF571	-2.376233048	1.47E-17	4.03E-17	down
BLOC1S1-RD	-2.374583311	2.59E-06	4.77E-06	down
AK9	-2.369396864	9.73E-17	2.60E-16	down
MANEA	-2.369226101	7.11E-99	7.93E-98	down
DUSP19	-2.366970127	2.57E-15	6.64E-15	down
CC2D2A	-2.364939591	1.47E-55	8.77E-55	down
ZNF449	-2.363694995	4.47E-35	1.84E-34	down
TCEAL9	-2.358300532	1.05E-47	5.50E-47	down
IMPACT	-2.358037002	1.54E-62	1.04E-61	down
ZFP14	-2.355474488	1.47E-20	4.33E-20	down
ADAT2	-2.35485384	1.42E-22	4.38E-22	down
CCDC169	-2.349048219	0.00243837	0.00373705	down
LYSMD1	-2.349048219	0.00243837	0.00373705	down
PDE10A	-2.349048219	0.00243837	0.00373705	down
TBC1D32	-2.349048219	1.07E-15	2.78E-15	down
WDR63	-2.349048219	0.00243837	0.00373705	down
ZNF345	-2.349048219	1.22E-11	2.81E-11	down
PEX1	-2.345824318	1.14E-57	7.05E-57	down
INSIG2	-2.337907661	1.28E-44	6.35E-44	down
CARF	-2.336109163	8.20E-11	1.83E-10	down
STEAP2	-2.336109163	1.76E-15	4.56E-15	down
PIGB	-2.330900872	3.61E-31	1.36E-30	down
RBM41	-2.327674568	5.71E-35	2.34E-34	down
EFCAB7	-2.326328142	2.76E-17	7.49E-17	down
LRRC39	-2.326328142	0.00055583	0.00089405	down
ZNF681	-2.320069152	2.36E-44	1.17E-43	down
RDH14	-2.316480356	1.09E-19	3.14E-19	down
CORO2B	-2.312522343	0.00012934	0.00021678	down
FAM227B	-2.312522343	0.00012934	0.00021678	down
FRMD3	-2.312522343	1.93E-14	4.84E-14	down
FAM122C	-2.299660192	9.12E-10	1.96E-09	down
DYNC2LI1	-2.298285105	2.75E-31	1.04E-30	down
ARMCX3	-2.295565888	1.21E-100	1.38E-99	down
GULP1	-2.293906665	1.66E-123	2.48E-122	down
RAD54B	-2.291562724	1.14E-26	3.87E-26	down
ZNF519	-2.291562724	1.37E-11	3.15E-11	down
ZNF823	-2.289491374	3.41E-12	7.96E-12	down
ETAA1	-2.287157545	4.43E-81	3.92E-80	down

PUS7L	-2.28281452	7.56E-146	1.43E-144	down
CDC14C	-2.281934023	2.51E-08	5.08E-08	down
ODF2L	-2.28170191	2.76E-138	4.80E-137	down
ZNF891	-2.280288676	1.21E-30	4.51E-30	down
LZTFL1	-2.280070477	8.87E-53	5.07E-52	down
FOXN2	-2.278433452	3.36E-173	8.47E-172	down
C11orf54	-2.272812633	1.98E-22	6.10E-22	down
C6orf163	-2.255938814	0.00418112	0.00627304	down
CDRT15	-2.255938814	0.01935721	0.02718686	down
CYP3A5	-2.255938814	0.00418112	0.00627304	down
DCDC1	-2.255938814	0.01935721	0.02718686	down
ELAVL2	-2.255938814	0.01935721	0.02718686	down
EYS	-2.255938814	0.00094216	0.00149091	down
FCRLB	-2.255938814	0.01935721	0.02718686	down
HCAR3	-2.255938814	0.01935721	0.02718686	down
HESX1	-2.255938814	1.21E-05	2.16E-05	down
PEX5L	-2.255938814	0.01935721	0.02718686	down
PTGS2	-2.255938814	2.91E-06	5.34E-06	down
RTKN2	-2.255938814	1.42E-34	5.78E-34	down
VMAC	-2.255938814	0.00094216	0.00149091	down
ZNF700	-2.255938814	5.34E-19	1.51E-18	down
ZNF98	-2.255938814	1.51E-10	3.32E-10	down
BRWD3	-2.250650983	6.71E-99	7.49E-98	down
DYNC2H1	-2.247901495	5.35E-108	6.68E-107	down
KPNA5	-2.247551029	1.64E-42	7.81E-42	down
POC1B	-2.24446603	3.58E-61	2.36E-60	down
CEP112	-2.242133015	5.76E-14	1.42E-13	down
TMEM106B	-2.236366072	1.03E-165	2.42E-164	down
VAMP4	-2.233571001	1.41E-54	8.29E-54	down
CEP97	-2.233024422	2.20E-97	2.40E-96	down
LYPD6	-2.229466603	3.81E-27	1.31E-26	down
PRDM5	-2.229466603	6.84E-08	1.36E-07	down
CEP126	-2.223517337	1.16E-06	2.18E-06	down
DCUN1D4	-2.222828338	7.26E-138	1.26E-136	down
GPR180	-2.22066334	7.55E-120	1.09E-118	down
TRMT10A	-2.207029214	3.89E-15	9.96E-15	down
MSANTD4	-2.20486463	5.30E-79	4.58E-78	down
NUDT12	-2.20486463	2.79E-53	1.61E-52	down
MTRF1	-2.204539662	4.08E-24	1.31E-23	down
PRKAA2	-2.20061174	5.14E-58	3.20E-57	down
POLK	-2.199640235	8.05E-92	8.16E-91	down
SYT13	-2.197045125	0.00036452	0.00059267	down
JRKL	-2.195817822	7.15E-29	2.56E-28	down
ZNF484	-2.193572933	1.86E-30	6.92E-30	down
NREP	-2.192162904	1.51E-42	7.21E-42	down
PHF6	-2.190740548	0	0	down
NOX1	-2.190350473	1.91E-06	3.55E-06	down
SPART	-2.190092365	3.40E-73	2.71E-72	down
IL7	-2.187767312	1.10E-08	2.27E-08	down
TSTD3	-2.187767312	1.10E-08	2.27E-08	down
LRRC49	-2.183988972	6.77E-22	2.06E-21	down
SCRN3	-2.18348702	1.88E-28	6.66E-28	down
CENPQ	-2.182924017	1.04E-43	5.09E-43	down
FPGT	-2.181938233	1.82E-47	9.55E-47	down

CCDC14	-2.181277809	1.26E-126	1.93E-125	down
ELMOD2	-2.173476654	6.88E-60	4.42E-59	down
STARD4	-2.171138412	0	0	down
C5orf63	-2.171049917	3.34E-05	5.79E-05	down
CDH8	-2.171049917	4.42E-09	9.26E-09	down
MBIP	-2.17020894	1.66E-57	1.02E-56	down
TMEM170B	-2.168475973	2.57E-56	1.56E-55	down
ZC2HC1A	-2.168475973	3.15E-47	1.64E-46	down
HOXC8	-2.166671476	7.61E-07	1.44E-06	down
MLLT11	-2.164308339	1.69E-15	4.39E-15	down
SLC16A7	-2.161962666	1.23E-60	8.03E-60	down
NBEAL1	-2.161941324	1.07E-70	8.20E-70	down
ATAD5	-2.159777868	1.75E-98	1.94E-97	down
FRA10AC1	-2.159185581	2.88E-21	8.62E-21	down
THAP5	-2.15824214	2.14E-90	2.12E-89	down
CD160	-2.156403141	0.00712436	0.01046295	down
ZNF713	-2.150328626	1.76E-10	3.87E-10	down
ZNF680	-2.150105902	1.31E-44	6.50E-44	down
ZNF260	-2.148732813	4.65E-121	6.78E-120	down
POLI	-2.147931625	1.54E-33	6.12E-33	down
MAP9	-2.147004443	1.46E-47	7.65E-47	down
DIXDC1	-2.143464085	9.23E-31	3.45E-30	down
STYK1	-2.143464085	1.31E-05	2.33E-05	down
ZFP37	-2.143464085	1.33E-26	4.49E-26	down
KITLG	-2.140885234	7.69E-119	1.09E-117	down
ZBTB10	-2.138899393	4.85E-81	4.28E-80	down
TRIQK	-2.137410425	2.92E-38	1.28E-37	down
EMB	-2.136578162	2.85E-208	9.40E-207	down
LONRF2	-2.135644581	0.00060751	0.00097453	down
TLR3	-2.135644581	0.00060751	0.00097453	down
FAM111B	-2.126952374	1.61E-232	6.21E-231	down
SCN9A	-2.126655797	5.51E-05	9.43E-05	down
ZNF280D	-2.126655797	1.40E-26	4.74E-26	down
SASS6	-2.125962027	1.34E-67	9.75E-67	down
MOSPD2	-2.118435291	6.50E-46	3.32E-45	down
RAB27B	-2.116874777	3.15E-20	9.18E-20	down
NAP1L5	-2.116214051	1.16E-14	2.93E-14	down
ALG10	-2.114131488	3.53E-25	1.16E-24	down
GCSH	-2.112347961	4.60E-11	1.03E-10	down
HECTD2	-2.111548905	1.56E-43	7.61E-43	down
KCNJ16	-2.109860555	5.94E-42	2.80E-41	down
PJA2	-2.108293003	6.82E-274	3.40E-272	down
SLITRK4	-2.107342322	5.68E-25	1.85E-24	down
CHIC1	-2.106223897	7.09E-71	5.45E-70	down
SGCE	-2.105553999	2.27E-99	2.56E-98	down
FRMD4B	-2.103935721	1.91E-16	5.07E-16	down
HOXA2	-2.103935721	0.03364422	0.04587832	down
KLRB1	-2.103935721	0.03364422	0.04587832	down
PHACTR1	-2.103935721	0.03364422	0.04587832	down
PHOSPHO2	-2.103935721	1.87E-09	3.98E-09	down
PLXNB3	-2.103935721	0.03364422	0.04587832	down
PRRG4	-2.103935721	2.15E-05	3.77E-05	down
SBSPON	-2.103935721	0.03364422	0.04587832	down
SLC16A4	-2.103935721	0.00023377	0.00038502	down

SLC16A9	-2.103935721	0.00023377	0.00038502	down
SLC23A3	-2.103935721	0.03364422	0.04587832	down
LPP	-2.10302979	3.01E-262	1.42E-260	down
N4BP2L2	-2.101415729	2.02E-95	2.15E-94	down
RAB8B	-2.100521056	7.41E-71	5.70E-70	down
ZNF624	-2.099596785	8.14E-20	2.36E-19	down
SCAMP1	-2.099462244	1.33E-159	2.89E-158	down
ZBTB20	-2.099213311	4.94E-35	2.03E-34	down
ARHGAP5	-2.09371105	1.47E-230	5.61E-229	down
SLC33A1	-2.093209314	1.83E-45	9.21E-45	down
RGS17	-2.092158553	1.59E-41	7.45E-41	down
NHLRC2	-2.090428215	1.71E-151	3.42E-150	down
SMARCE1	-2.090129921	3.18E-103	3.74E-102	down
EXPH5	-2.087816056	2.98E-11	6.73E-11	down
XKR9	-2.086013813	8.42E-06	1.51E-05	down
FAM161A	-2.085076694	9.38E-39	4.17E-38	down
KIAA1551	-2.082209158	2.73E-296	1.51E-294	down
STEAP1B	-2.082109358	2.10E-19	6.00E-19	down
FAR1	-2.081815164	2.22E-301	1.26E-299	down
KSR2	-2.080852108	9.04E-05	0.00015303	down
MDM1	-2.080852108	7.51E-32	2.88E-31	down
ZFHX2	-2.080852108	9.04E-05	0.00015303	down
SNX10	-2.078523302	1.98E-68	1.46E-67	down
PROS1	-2.075879436	1.87E-108	2.34E-107	down
TRPS1	-2.074873255	3.43E-20	1.00E-19	down
CNTF	-2.071514243	3.31E-06	6.06E-06	down
HOXC6	-2.071514243	0.00100707	0.00159135	down
IL18	-2.071514243	3.31E-06	6.06E-06	down
LRRC40	-2.071514243	2.92E-117	4.10E-116	down
DNAAF4	-2.066461015	1.26E-07	2.47E-07	down
ZNF268	-2.066235012	2.95E-54	1.72E-53	down
PLA2R1	-2.065967871	6.84E-74	5.50E-73	down
KBTBD7	-2.063293736	6.92E-28	2.42E-27	down
PKIA	-2.063293736	8.71E-58	5.39E-57	down
POU6F1	-2.063293736	3.51E-05	6.08E-05	down
ZNF391	-2.063293736	1.29E-16	3.43E-16	down
ZNF610	-2.063293736	3.51E-05	6.08E-05	down
EEA1	-2.06230412	3.38E-118	4.77E-117	down
TRIM2	-2.060187825	5.21E-45	2.60E-44	down
ZNF182	-2.059211276	5.03E-44	2.48E-43	down
FANCF	-2.052655216	8.45E-18	2.32E-17	down
C2CD5	-2.049487937	4.47E-102	5.20E-101	down
CRISPLD1	-2.049487937	0.01205235	0.01734375	down
SLC27A5	-2.049487937	0.01205235	0.01734375	down
ZNF138	-2.049487937	1.24E-30	4.64E-30	down
NUCB2	-2.046564542	7.95E-54	4.61E-53	down
HMGCS1	-2.044073945	0	0	down
AKAP7	-2.041814009	8.08E-15	2.05E-14	down
SKAP2	-2.035994752	1.92E-72	1.51E-71	down
PHIP	-2.035326399	0	0	down
KLF8	-2.033546393	0.00014783	0.00024684	down
NES	-2.033546393	8.03E-08	1.59E-07	down
SPAG16	-2.033546393	1.02E-23	3.23E-23	down
ZKSCAN8	-2.032500583	9.20E-105	1.10E-103	down

ZNF33A	-2.031109408	4.17E-37	1.78E-36	down
AP1AR	-2.028869906	1.38E-93	1.42E-92	down
CEP44	-2.028246789	2.45E-46	1.26E-45	down
TRIM45	-2.027120124	5.19E-15	1.32E-14	down
TIA1	-2.026968827	3.01E-200	9.31E-199	down
ARHGAP18	-2.025856435	9.63E-105	1.16E-103	down
CCZ1	-2.021473561	1.26E-08	2.59E-08	down
LEAP2	-2.021473561	5.72E-05	9.78E-05	down
MPP7	-2.021473561	0.0044338	0.0066373	down
RNF150	-2.021473561	8.28E-07	1.57E-06	down
ZNF233	-2.021473561	0.0044338	0.0066373	down
MIS18BP1	-2.021294132	3.42E-210	1.14E-208	down
TENT2	-2.020760238	1.10E-106	1.35E-105	down
G2E3	-2.019023469	1.47E-184	4.10E-183	down
RASSF8	-2.018899617	8.42E-147	1.62E-145	down
ITGB6	-2.01647288	5.02E-09	1.05E-08	down
PIGK	-2.016327322	1.37E-182	3.78E-181	down
PPP2R3A	-2.015109145	3.95E-66	2.82E-65	down
EFHC1	-2.013864027	1.53E-55	9.12E-55	down
KRIT1	-2.013445187	6.87E-25	2.24E-24	down
ASAH2	-2.012013231	2.22E-05	3.89E-05	down
PMFBP1	-2.012013231	2.22E-05	3.89E-05	down
FAM135A	-2.011754498	4.54E-257	2.05E-255	down
ATP5MG	-2.01117858	2.07E-41	9.66E-41	down
GPR137C	-2.009608338	4.19E-36	1.76E-35	down
ATM	-2.009258562	7.10E-265	3.42E-263	down
UBA6	-2.008756962	5.61E-295	3.08E-293	down
ZNF253	-2.008011301	1.83E-26	6.18E-26	down
TRAPPC13	-2.005093817	1.52E-44	7.53E-44	down
SEMA3E	-2.004400047	0.0016597	0.00257914	down
TBCEL	-2.004400047	2.02E-12	4.77E-12	down
ZNF620	-2.001755326	8.10E-13	1.93E-12	down
CLIP4	-2.001601414	1.04E-75	8.63E-75	down
ZNF25	-2.00009991	2.02E-08	4.11E-08	down
CCDC91	-1.999924836	1.52E-54	8.94E-54	down
ZNF737	-1.998966161	5.67E-18	1.57E-17	down
NMRK1	-1.998141057	5.04E-11	1.13E-10	down
ZRANB3	-1.997626819	7.77E-28	2.71E-27	down
FSD1L	-1.995174582	6.52E-56	3.92E-55	down
AP1S2	-1.994446573	2.23E-81	1.98E-80	down
ZNF37A	-1.992449945	1.03E-55	6.16E-55	down
TMEM47	-1.992042841	1.18E-58	7.45E-58	down
ZMYM6	-1.992042841	3.40E-30	1.26E-29	down
C11orf74	-1.988458503	3.62E-18	1.00E-17	down
KLHL28	-1.981945196	9.55E-33	3.74E-32	down
XRCC4	-1.977805837	3.73E-32	1.44E-31	down
ZNF518A	-1.976445986	1.82E-57	1.12E-56	down
PLEKHG1	-1.975830895	1.28E-08	2.63E-08	down
ZSCAN31	-1.975830895	1.28E-08	2.63E-08	down
NIPSNAP3A	-1.972929709	1.02E-54	5.99E-54	down
NDUFA5	-1.972189257	4.57E-121	6.68E-120	down
SHPRH	-1.972145848	1.28E-59	8.20E-59	down
RFX3	-1.969634629	5.27E-14	1.30E-13	down
TIGD7	-1.969634629	5.27E-14	1.30E-13	down

IER3IP1	-1.965933124	5.59E-107	6.91E-106	down
KIAA1109	-1.964878286	6.93E-120	9.97E-119	down
CEP85L	-1.964599039	5.49E-16	1.44E-15	down
SEMA3A	-1.963758063	2.13E-06	3.94E-06	down
LACC1	-1.962579872	8.12E-12	1.87E-11	down
PRIM1	-1.961844928	3.92E-33	1.54E-32	down
TMEM237	-1.960877325	6.56E-110	8.40E-109	down
ZNF615	-1.958505281	2.30E-31	8.74E-31	down
FNBP1L	-1.956828802	3.43E-141	6.13E-140	down
CHD9	-1.956441766	1.55E-143	2.84E-142	down
PLEKHH2	-1.955705791	5.38E-15	1.37E-14	down
RETREG1	-1.955072335	2.04E-08	4.14E-08	down
ZGRF1	-1.953910277	4.83E-38	2.11E-37	down
ZNF708	-1.953639526	3.63E-31	1.37E-30	down
CEP120	-1.952747282	2.47E-63	1.69E-62	down
AP5M1	-1.951498146	1.62E-100	1.84E-99	down
MGST1	-1.950884538	5.44E-70	4.11E-69	down
F8	-1.947307542	2.71E-23	8.52E-23	down
OSBPL8	-1.946422855	0	0	down
TM4SF18	-1.94571907	1.10E-306	6.36E-305	down
PIK3C2A	-1.942780929	2.97E-164	6.81E-163	down
GPR19	-1.934010719	0.00733018	0.01074789	down
KCNAB1	-1.934010719	0.00014934	0.00024927	down
METTL4	-1.934010719	1.61E-44	7.99E-44	down
MPZL3	-1.934010719	0.00733018	0.01074789	down
PDIK1L	-1.934010719	1.09E-23	3.46E-23	down
PLEKHA4	-1.934010719	0.02022153	0.02832591	down
PRMT9	-1.934010719	1.66E-22	5.13E-22	down
SPATA25	-1.934010719	0.02022153	0.02832591	down
SPATA7	-1.934010719	5.09E-09	1.06E-08	down
ZBTB8A	-1.934010719	9.09E-18	2.50E-17	down
ZNF540	-1.934010719	0.00733018	0.01074789	down
ENPP4	-1.932643884	5.08E-105	6.12E-104	down
ZNF189	-1.930504776	5.70E-42	2.69E-41	down
GGPS1	-1.929810731	2.70E-35	1.12E-34	down
KNTC1	-1.928205098	3.81E-195	1.14E-193	down
ZWILCH	-1.927646814	4.58E-112	6.04E-111	down
ZMYM1	-1.927438065	3.20E-87	3.03E-86	down
GOLPH3L	-1.924487945	6.77E-46	3.45E-45	down
CXADR	-1.921662327	1.31E-69	9.84E-69	down
ETFRF1	-1.921071664	2.05E-12	4.82E-12	down
PYGO1	-1.921071664	2.69E-23	8.46E-23	down
ZNF292	-1.920231292	7.01E-103	8.20E-102	down
ZNF462	-1.919702883	3.30E-60	2.13E-59	down
IRAK1BP1	-1.918243404	2.37E-19	6.78E-19	down
SLC35A3	-1.918243404	1.54E-63	1.06E-62	down
ARL5B	-1.917605924	4.96E-78	4.23E-77	down
DYNLT3	-1.917562173	3.91E-162	8.75E-161	down
SPEF2	-1.916732728	1.27E-09	2.72E-09	down
CCDC150	-1.916221406	8.47E-41	3.90E-40	down
ZNF302	-1.915863373	5.92E-63	4.03E-62	down
MCC	-1.914381913	8.61E-16	2.25E-15	down
ZNF230	-1.913252159	5.34E-15	1.36E-14	down
CBWD6	-1.912637069	1.33E-14	3.36E-14	down

LTN1	-1.911404568	2.50E-154	5.14E-153	down
AGL	-1.911012028	1.19E-157	2.54E-156	down
DDX60L	-1.909763173	1.75E-24	5.65E-24	down
KIAA1586	-1.909763173	6.82E-36	2.85E-35	down
CEP70	-1.906840518	2.23E-52	1.26E-51	down
THAP6	-1.906840518	4.80E-27	1.64E-26	down
OGFRL1	-1.906632276	0	0	down
TRPC1	-1.905996343	1.03E-55	6.18E-55	down
ZNF678	-1.905996343	6.59E-46	3.36E-45	down
ZNF175	-1.90414019	1.11E-24	3.59E-24	down
NEB	-1.90363707	5.43E-06	9.84E-06	down
KIAA0391	-1.90230186	3.18E-10	6.93E-10	down
CCNG2	-1.900501457	1.88E-38	8.30E-38	down
SMARCA1	-1.900489524	0	0	down
GNPDA2	-1.899115929	1.39E-52	7.93E-52	down
CCDC39	-1.897484843	5.05E-09	1.05E-08	down
ARL6IP5	-1.897330056	1.36E-179	3.61E-178	down
ZHX1	-1.896208687	8.21E-99	9.14E-98	down
ZNF148	-1.89393328	5.67E-110	7.27E-109	down
ARHGEF35	-1.893368735	9.23E-05	0.00015605	down
RAD51AP1	-1.891623409	1.71E-167	4.12E-166	down
TMEM267	-1.891598557	7.36E-88	7.03E-87	down
LGR4	-1.891081814	9.88E-175	2.52E-173	down
SMARCAD1	-1.890884774	1.18E-142	2.14E-141	down
ZBTB33	-1.890523182	4.83E-98	5.33E-97	down
MACROD2	-1.8896166	2.89E-38	1.27E-37	down
MRPL42	-1.889049534	1.61E-184	4.48E-183	down
SLC38A6	-1.88820703	2.78E-25	9.11E-25	down
SREK1IP1	-1.887941071	4.00E-66	2.86E-65	down
FKTN	-1.886306246	2.06E-145	3.90E-144	down
PARPBP	-1.884479134	6.00E-75	4.92E-74	down
ERO1B	-1.882856631	2.52E-46	1.30E-45	down
DIAPH2	-1.882838067	5.12E-62	3.43E-61	down
RBMS3	-1.882785396	2.19E-17	5.97E-17	down
C5orf51	-1.88232456	5.53E-201	1.73E-199	down
BRIP1	-1.882198772	1.41E-120	2.05E-119	down
UNC13C	-1.8815433	1.32E-06	2.48E-06	down
ZNF790	-1.880239463	1.35E-16	3.60E-16	down
PPM1K	-1.879267755	8.98E-19	2.53E-18	down
DNAJB14	-1.878157485	2.76E-116	3.82E-115	down
NME7	-1.876677544	1.26E-29	4.61E-29	down
CFAP97	-1.875844012	9.51E-114	1.28E-112	down
PLA2G4A	-1.873750195	1.52E-21	4.60E-21	down
STXBP4	-1.873672934	1.45E-65	1.03E-64	down
RAPGEF5	-1.872610175	2.56E-36	1.08E-35	down
CENPK	-1.871426182	6.36E-97	6.88E-96	down
FAM214A	-1.870816893	3.22E-14	8.03E-14	down
ZNF492	-1.870648658	1.05E-24	3.42E-24	down
PGAP1	-1.870368224	4.84E-62	3.25E-61	down
BIVM	-1.868915691	5.76E-20	1.67E-19	down
ZBTB38	-1.8682273	5.38E-94	5.60E-93	down
LIPT1	-1.867777021	1.31E-15	3.39E-15	down
FBXO4	-1.867668225	1.43E-33	5.69E-33	down
MATN2	-1.864748057	3.24E-07	6.24E-07	down

JAZF1	-1.863237645	2.18E-18	6.09E-18	down
SWT1	-1.862457458	1.25E-12	2.96E-12	down
C14orf93	-1.860010138	5.68E-05	9.72E-05	down
SEMA3D	-1.860010138	0.00442005	0.00661788	down
IFT74	-1.858459686	2.45E-36	1.04E-35	down
INTU	-1.858459686	5.52E-19	1.56E-18	down
SLC25A36	-1.858148705	7.56E-160	1.66E-158	down
C18orf54	-1.85787965	2.08E-152	4.20E-151	down
TOP2B	-1.85756113	0	0	down
BHLHB9	-1.856842859	4.90E-10	1.06E-09	down
ACADSB	-1.856728798	1.09E-50	6.06E-50	down
DMRTA1	-1.854576252	2.09E-06	3.86E-06	down
YTHDC2	-1.854576252	5.22E-144	9.68E-143	down
NEDD1	-1.854438767	1.75E-140	3.11E-139	down
BTN3A1	-1.851548559	4.87E-11	1.09E-10	down
MAML3	-1.851548559	0.00014701	0.00024554	down
SHTN1	-1.851270288	3.57E-108	4.46E-107	down
RPS6KA6	-1.85104927	4.15E-61	2.73E-60	down
TAF9B	-1.85079334	1.68E-197	5.10E-196	down
GNAI1	-1.849398492	2.92E-29	1.05E-28	down
UBLCP1	-1.848894106	1.43E-68	1.05E-67	down
ZNF605	-1.848280845	6.29E-38	2.75E-37	down
XRN1	-1.84766987	1.90E-85	1.76E-84	down
ZFC3H1	-1.846309738	3.68E-100	4.17E-99	down
GLRB	-1.846137963	8.94E-21	2.65E-20	down
FRRS1	-1.844743381	3.05E-10	6.66E-10	down
ATP8A1	-1.844214049	9.13E-22	2.77E-21	down
PMS1	-1.84381291	9.00E-59	5.68E-58	down
CTPS2	-1.843037059	3.26E-17	8.84E-17	down
CRYZ	-1.842981625	6.12E-161	1.36E-159	down
PLCB4	-1.841752212	1.38E-79	1.20E-78	down
CCDC18	-1.841646701	3.35E-46	1.72E-45	down
AC010326.2	-1.840901315	1.36E-05	2.42E-05	down
DLG2	-1.840901315	0.00038258	0.00062097	down
FAR2	-1.840901315	0.00038258	0.00062097	down
HSD17B11	-1.840901315	1.60E-103	1.89E-102	down
TMBIM4	-1.840901315	3.31E-18	9.20E-18	down
HSPA13	-1.840509757	1.31E-254	5.84E-253	down
ANKRD36	-1.838853486	8.22E-18	2.26E-17	down
ACBD5	-1.838018806	5.50E-59	3.49E-58	down
ZNF10	-1.8373347	3.44E-20	1.00E-19	down
RASEF	-1.836943301	1.16E-76	9.73E-76	down
ZNF254	-1.836002516	4.39E-42	2.07E-41	down
1-3月	-1.834475046	1.29E-06	2.42E-06	down
LNPK	-1.834175452	7.37E-119	1.05E-117	down
HINT3	-1.83318635	8.65E-36	3.61E-35	down
CHML	-1.832796265	2.22E-163	5.03E-162	down
GCA	-1.832537842	1.42E-60	9.22E-60	down
DBT	-1.831649002	2.44E-51	1.36E-50	down
VWDE	-1.83063298	1.28E-17	3.50E-17	down
SCP2	-1.82812271	2.69E-129	4.23E-128	down
ANKRD18B	-1.827095516	2.41E-25	7.91E-25	down
FASTKD1	-1.827095516	5.24E-82	4.69E-81	down
ZNF81	-1.825757829	1.08E-29	3.96E-29	down

PTAR1	-1.825387173	1.26E-154	2.62E-153	down
TMEM161B	-1.822879907	2.21E-50	1.22E-49	down
DNAJC10	-1.821573231	0	0	down
C11orf1	-1.82153599	3.06E-08	6.16E-08	down
SOCS4	-1.819474288	6.00E-172	1.50E-170	down
TSNAX	-1.818358746	4.71E-59	2.99E-58	down
DMXL1	-1.817925301	8.14E-117	1.14E-115	down
DCAF17	-1.816795106	2.95E-59	1.88E-58	down
FAM126B	-1.816653769	4.42E-92	4.50E-91	down
RBL2	-1.816174229	6.25E-154	1.28E-152	down
BMI1	-1.816063009	1.81E-70	1.39E-69	down
DPY19L3	-1.814749027	4.23E-57	2.60E-56	down
ZRANB2	-1.813237757	9.79E-229	3.70E-227	down
AKAP5	-1.812704423	7.33E-12	1.69E-11	down
CLDN12	-1.811365806	1.38E-76	1.16E-75	down
PEG10	-1.810304169	0	0	down
ACTR3C	-1.808479837	0.00023306	0.00038393	down
AIFM3	-1.808479837	0.0336068	0.04586162	down
ALS2CR12	-1.808479837	0.0336068	0.04586162	down
BLOC1S5	-1.808479837	3.07E-36	1.30E-35	down
C14orf28	-1.808479837	2.02E-06	3.75E-06	down
C1orf162	-1.808479837	0.0336068	0.04586162	down
EFEMP2	-1.808479837	0.0336068	0.04586162	down
FMO5	-1.808479837	0.0336068	0.04586162	down
GPX3	-1.808479837	0.0336068	0.04586162	down
KLHL13	-1.808479837	2.05E-21	6.19E-21	down
SMPDL3B	-1.808479837	0.00265701	0.00405532	down
SPDYA	-1.808479837	0.0336068	0.04586162	down
SRSF12	-1.808479837	1.83E-11	4.17E-11	down
TNIK	-1.808479837	0.0336068	0.04586162	down
ZNF507	-1.808479837	9.83E-69	7.30E-68	down
CEP152	-1.807430223	9.49E-43	4.55E-42	down
SLK	-1.80741747	1.89E-163	4.28E-162	down
TTN	-1.806591493	2.77E-47	1.44E-46	down
HAUS6	-1.80646067	4.58E-108	5.72E-107	down
ZNF107	-1.80646067	2.50E-44	1.23E-43	down
ARL1	-1.804968774	8.48E-124	1.27E-122	down
ZNF420	-1.804727702	1.37E-24	4.44E-24	down
STAU2	-1.80367352	6.14E-82	5.48E-81	down
SLC39A10	-1.801255511	6.68E-120	9.62E-119	down
MTFR2	-1.801008588	2.48E-30	9.18E-30	down
GLS	-1.800082287	0	0	down
WDHD1	-1.799135434	3.12E-170	7.67E-169	down
ERGIC2	-1.797543904	7.90E-103	9.23E-102	down
IPO8	-1.797402384	1.74E-235	6.83E-234	down
BRWD1	-1.795692804	2.47E-144	4.60E-143	down
ZFP90	-1.795209224	1.55E-46	8.00E-46	down
PLS1	-1.79261499	1.28E-129	2.02E-128	down
PREPL	-1.792199851	8.53E-155	1.77E-153	down
METTL25	-1.791991715	1.25E-06	2.34E-06	down
AKAP11	-1.791317463	8.45E-120	1.21E-118	down
LYRM7	-1.790801077	1.25E-68	9.25E-68	down
TMEM254	-1.790483408	7.87E-23	2.45E-22	down
VPS13A	-1.788969537	1.41E-122	2.08E-121	down

ZFAND1	-1.78849166	9.25E-61	6.05E-60	down
TBL1XR1	-1.78819946	3.01E-248	1.30E-246	down
ERI2	-1.788159853	2.88E-18	8.02E-18	down
PCLO	-1.788159853	4.41E-14	1.10E-13	down
ITGB3BP	-1.787406389	1.15E-53	6.63E-53	down
CNTLN	-1.787385806	1.18E-39	5.31E-39	down
CCDC15	-1.78591208	1.10E-14	2.79E-14	down
ZNF888	-1.78581924	9.54E-102	1.10E-100	down
CEP162	-1.784563331	1.47E-31	5.60E-31	down
VPS50	-1.783817783	7.94E-58	4.93E-57	down
PHF11	-1.783519093	1.79E-18	5.02E-18	down
FAM92A	-1.783279283	1.21E-41	5.65E-41	down
AC124312.1	-1.782007626	0.00014181	0.00023711	down
DAAM2	-1.782007626	0.0071339	0.01047575	down
GPR158	-1.782007626	1.09E-12	2.60E-12	down
WDR5B	-1.782007626	1.79E-09	3.80E-09	down
FANCM	-1.780098036	1.85E-41	8.65E-41	down
NPHP1	-1.780098036	6.86E-15	1.75E-14	down
DLG3	-1.779288125	2.87E-20	8.38E-20	down
BBOF1	-1.778359849	1.82E-08	3.70E-08	down
SYNJ2BP	-1.778139809	6.07E-80	5.28E-79	down
DSG2	-1.777974369	0	0	down
ZNF606	-1.777285216	2.72E-12	6.37E-12	down
SLX4IP	-1.776271256	1.19E-23	3.76E-23	down
GRAMD2B	-1.775312973	4.47E-09	9.36E-09	down
CEP57	-1.774287031	1.33E-144	2.47E-143	down
ZNF836	-1.774230275	2.72E-11	6.16E-11	down
CEBPZOS	-1.773187925	1.22E-75	1.01E-74	down
PON2	-1.772997251	3.83E-110	4.94E-109	down
NAB1	-1.77288973	1.47E-122	2.18E-121	down
CCDC68	-1.772729812	3.63E-34	1.47E-33	down
IGIP	-1.770511987	0.00159595	0.00248432	down
MTERF2	-1.770511987	1.82E-23	5.76E-23	down
ZNF441	-1.770511987	4.56E-08	9.13E-08	down
BRCA2	-1.770015846	5.61E-110	7.20E-109	down
MAOA	-1.768951473	1.16E-24	3.77E-24	down
CHMP2B	-1.768128873	4.04E-129	6.36E-128	down
ZNF91	-1.767439153	8.16E-31	3.05E-30	down
OXR1	-1.767396931	7.94E-65	5.56E-64	down
NMU	-1.766900734	1.94E-06	3.60E-06	down
TRMT13	-1.766900734	2.07E-31	7.85E-31	down
ZNF736	-1.766066082	6.38E-58	3.96E-57	down
C1GALT1	-1.76285722	1.18E-80	1.04E-79	down
TMEM56	-1.76244536	2.08E-52	1.18E-51	down
PIK3CA	-1.762378819	1.55E-58	9.74E-58	down
L3MBTL3	-1.760559207	2.67E-21	8.02E-21	down
TFPI	-1.757350362	3.33E-177	8.60E-176	down
CDC7	-1.755932783	2.17E-57	1.34E-56	down
RFX7	-1.755706665	1.29E-88	1.25E-87	down
KRR1	-1.754708581	2.24E-76	1.88E-75	down
STAM2	-1.753581591	1.88E-103	2.22E-102	down
LRRC6	-1.753438474	1.19E-06	2.23E-06	down
NXT2	-1.753132137	2.72E-49	1.47E-48	down
PYROXD1	-1.752563848	2.52E-18	7.01E-18	down

HLTF	-1.750038516	5.07E-187	1.45E-185	down
MIER3	-1.749389207	6.42E-102	7.43E-101	down
FANCB	-1.74927084	2.47E-17	6.73E-17	down
NNT	-1.749004478	1.53E-179	4.07E-178	down
KIAA0586	-1.748707617	4.14E-69	3.09E-68	down
SCD	-1.748511248	0	0	down
DNAJC19	-1.748219313	9.89E-20	2.85E-19	down
RHOQ	-1.746523246	2.89E-109	3.66E-108	down
STK36	-1.744854628	2.44E-19	6.96E-19	down
ANP32E	-1.74411965	0	0	down
TMEM67	-1.744111018	3.98E-24	1.28E-23	down
POT1	-1.743290518	1.37E-65	9.73E-65	down
ACRV1	-1.741365641	0.01953852	0.02742188	down
C7orf25	-1.741365641	0.01953852	0.02742188	down
ETFBKMT	-1.741365641	0.00095888	0.00151689	down
GRIN3A	-1.741365641	0.00423782	0.00635511	down
LNP1	-1.741365641	0.0002224	0.0003667	down
PARM1	-1.741365641	0.0002224	0.0003667	down
POLR2M	-1.741365641	3.45E-67	2.50E-66	down
RASIP1	-1.741365641	0.01953852	0.02742188	down
SATB1	-1.741365641	6.49E-10	1.40E-09	down
THRB	-1.741365641	0.0002224	0.0003667	down
TMEM56-RW	-1.741365641	0.00423782	0.00635511	down
ZNF225	-1.741365641	3.83E-14	9.53E-14	down
RABGAP1L	-1.735784609	2.01E-45	1.01E-44	down
TRAF5	-1.735629272	5.93E-23	1.85E-22	down
BARD1	-1.735230855	5.26E-91	5.27E-90	down
ZSCAN26	-1.735106651	3.70E-21	1.11E-20	down
BMT2	-1.73351778	5.93E-25	1.93E-24	down
ZNF181	-1.733416888	5.79E-17	1.56E-16	down
MCOLN3	-1.733228592	4.41E-39	1.97E-38	down
PSMA2	-1.733098025	6.56E-09	1.36E-08	down
ZNF829	-1.733098025	9.29E-24	2.95E-23	down
TET2	-1.73132597	6.23E-32	2.39E-31	down
SMC2	-1.731239912	0	0	down
CD2AP	-1.730504649	2.39E-235	9.38E-234	down
PEX12	-1.730477325	4.47E-07	8.56E-07	down
ZNF682	-1.730477325	4.47E-07	8.56E-07	down
ZMYM2	-1.727845836	1.56E-86	1.47E-85	down
CCDC82	-1.727104948	1.66E-90	1.65E-89	down
DNAL1	-1.727081415	1.57E-35	6.51E-35	down
NAE1	-1.726549978	4.27E-233	1.66E-231	down
RIOK2	-1.725726518	2.37E-63	1.62E-62	down
ERCC4	-1.725424098	9.73E-36	4.06E-35	down
ACSL4	-1.724827114	0	0	down
CD109	-1.72423967	2.69E-299	1.51E-297	down
RNF125	-1.72408765	1.64E-08	3.35E-08	down
HMSD	-1.72359094	9.38E-12	2.16E-11	down
OPN1SW	-1.722506614	6.69E-08	1.33E-07	down
SYCP2	-1.722506614	0.0001346	0.00022542	down
ZNF649	-1.722506614	0.0001346	0.00022542	down
RNF217	-1.722237329	1.61E-42	7.69E-42	down
UFL1	-1.72213617	5.34E-61	3.50E-60	down
UMAD1	-1.721515866	5.84E-35	2.40E-34	down

CASD1	-1.721204347	2.89E-52	1.63E-51	down
STAG2	-1.721009114	5.16E-301	2.91E-299	down
RMI1	-1.720745542	2.60E-45	1.30E-44	down
HORMAD1	-1.720607081	2.74E-07	5.30E-07	down
CWC22	-1.720534731	4.96E-85	4.59E-84	down
TSPAN6	-1.720180312	4.02E-44	1.98E-43	down
RPGRIP1L	-1.719885914	3.19E-60	2.06E-59	down
TROVE2	-1.719070602	2.92E-154	6.00E-153	down
ZNF770	-1.718531543	7.30E-109	9.20E-108	down
EFHB	-1.718282028	0.00057655	0.00092631	down
ZC3H12B	-1.718282028	5.79E-12	1.34E-11	down
GALNT7	-1.717877289	9.72E-101	1.11E-99	down
HFE	-1.717301937	5.35E-29	1.92E-28	down
ZNF621	-1.716947929	9.60E-102	1.11E-100	down
APH1B	-1.716703587	1.33E-18	3.72E-18	down
CHURC1	-1.716419284	1.42E-37	6.14E-37	down
GOLT1B	-1.716268146	1.81E-160	3.98E-159	down
LGALSL	-1.716165087	1.44E-39	6.49E-39	down
CARNMT1	-1.715561886	5.78E-41	2.67E-40	down
ICK	-1.715000937	5.17E-22	1.58E-21	down
ZNF569	-1.714348091	9.48E-46	4.81E-45	down
ZBTB26	-1.713946353	1.39E-38	6.16E-38	down
OTUD6B	-1.713028695	1.81E-62	1.22E-61	down
SCAPER	-1.712654345	3.27E-18	9.08E-18	down
CXCL6	-1.711618298	0.00252225	0.00385772	down
ZNF608	-1.711618298	0.00252225	0.00385772	down
BET1	-1.710114707	3.59E-47	1.87E-46	down
METTL15	-1.70988741	3.39E-41	1.57E-40	down
SLC25A43	-1.709869789	1.48E-50	8.17E-50	down
TEX9	-1.709304432	6.20E-09	1.29E-08	down
FBXL17	-1.708451019	5.02E-34	2.02E-33	down
ASAH2B	-1.707734864	2.33E-10	5.10E-10	down
SCOC	-1.70703244	1.36E-116	1.89E-115	down
ZNF724	-1.706600223	2.52E-08	5.10E-08	down
ZNF117	-1.705386344	9.40E-10	2.01E-09	down
NHS	-1.705192029	7.62E-23	2.37E-22	down
PUS10	-1.703397791	5.23E-14	1.29E-13	down
GTPBP10	-1.702860375	5.27E-52	2.97E-51	down
PLK4	-1.70282518	5.45E-96	5.83E-95	down
CCDC66	-1.702685173	7.88E-17	2.11E-16	down
GOLGA8A	-1.701626526	1.98E-47	1.04E-46	down
ZNF267	-1.701258881	1.83E-29	6.64E-29	down
QSER1	-1.700437326	0	0	down
IFIH1	-1.700338373	1.14E-31	4.37E-31	down
CACNB4	-1.699545466	0.01141868	0.01646253	down
EBLN2	-1.699545466	0.000347	0.00056495	down
MBLAC2	-1.699545466	1.81E-25	5.96E-25	down
SSPN	-1.699545466	0.01141868	0.01646253	down
SYTL2	-1.699545466	0.000347	0.00056495	down
ZNF625	-1.699545466	0.01141868	0.01646253	down
PRKD3	-1.697955865	5.31E-180	1.42E-178	down
C4orf33	-1.697310461	3.36E-12	7.85E-12	down
KIF20B	-1.696834482	2.68E-132	4.38E-131	down
CALD1	-1.696812092	5.74E-226	2.13E-224	down

PCMTD2	-1.696319162	1.13E-45	5.73E-45	down
INVS	-1.695850982	1.09E-33	4.34E-33	down
ATP7A	-1.695711907	1.09E-38	4.85E-38	down
MED7	-1.695399788	1.75E-24	5.66E-24	down
PDGFC	-1.694361504	3.12E-115	4.25E-114	down
RWDD3	-1.694059927	3.55E-10	7.73E-10	down
TMF1	-1.694029188	2.16E-125	3.29E-124	down
SMC4	-1.693281984	0	0	down
STYX	-1.693110473	8.86E-86	8.24E-85	down
MDFIC	-1.693073898	8.53E-129	1.34E-127	down
CETN3	-1.69300262	6.75E-27	2.30E-26	down
ICE2	-1.692730866	1.01E-101	1.17E-100	down
COBLL1	-1.692456041	6.58E-35	2.70E-34	down
SLFN13	-1.692456041	2.89E-18	8.04E-18	down
SLC25A46	-1.69224493	2.16E-85	2.00E-84	down
SEC23A	-1.691201342	2.72E-282	1.41E-280	down
MMP16	-1.691033966	1.90E-15	4.92E-15	down
TMX3	-1.690635984	0	0	down
GCC2	-1.690556683	2.12E-90	2.10E-89	down
UBR1	-1.690291457	4.43E-65	3.11E-64	down
ZNF322	-1.690244647	1.61E-34	6.54E-34	down
DPY19L1	-1.689875013	6.22E-287	3.29E-285	down
ATRX	-1.689209933	8.40E-237	3.37E-235	down
RIMKLB	-1.68916943	6.53E-56	3.93E-55	down
ZSCAN16	-1.688898222	7.17E-06	1.29E-05	down
BTN3A2	-1.687850132	1.17E-15	3.05E-15	down
APOOL	-1.687606988	1.60E-58	1.01E-57	down
NECTIN3	-1.687413203	1.13E-80	9.89E-80	down
GEN1	-1.686373419	1.60E-65	1.13E-64	down
DZIP3	-1.685944904	7.22E-91	7.21E-90	down
TMED7	-1.685666214	5.30E-223	1.92E-221	down
SECISBP2L	-1.685656209	6.50E-74	5.23E-73	down
NRIP1	-1.685416125	5.94E-48	3.14E-47	down
TOGARAM1	-1.685242883	9.37E-46	4.76E-45	down
GAB1	-1.685190173	2.44E-29	8.84E-29	down
ZBTB37	-1.684983172	4.03E-24	1.29E-23	down
LANCL1	-1.684605761	1.65E-205	5.30E-204	down
PCMTD1	-1.684559286	6.53E-22	1.99E-21	down
PLEKHF2	-1.684310952	5.84E-40	2.64E-39	down
CAP2	-1.683495783	3.69E-61	2.43E-60	down
PBK	-1.683495783	1.98E-120	2.87E-119	down
SCYL2	-1.682948955	8.70E-139	1.52E-137	down
CPNE3	-1.681632495	0	0	down
CIP2A	-1.681609544	5.18E-207	1.69E-205	down
GRB14	-1.681244649	6.94E-17	1.86E-16	down
MYB	-1.681244649	2.99E-05	5.20E-05	down
DAAM1	-1.68072429	8.98E-34	3.59E-33	down
PLGRKT	-1.680562362	7.46E-14	1.84E-13	down
NEMP1	-1.680483399	3.89E-100	4.41E-99	down
TMEM135	-1.680336208	2.37E-93	2.44E-92	down
REL	-1.67986441	5.34E-50	2.93E-49	down
GDPD1	-1.679437892	4.37E-06	7.95E-06	down
LSM5	-1.679150245	3.38E-73	2.69E-72	down
FASTKD2	-1.679119487	8.83E-88	8.42E-87	down

MITD1	-1.67830896	3.30E-44	1.63E-43	down
RUFY2	-1.67830896	3.30E-44	1.63E-43	down
TTC3	-1.678214285	3.75E-275	1.88E-273	down
TBC1D19	-1.678171815	6.45E-07	1.23E-06	down
ZDHHC2	-1.677290088	5.31E-94	5.53E-93	down
AFG1L	-1.676514497	1.45E-08	2.96E-08	down
COG6	-1.676424858	1.25E-51	7.01E-51	down
ZNF43	-1.675477706	3.33E-10	7.25E-10	down
ZNF440	-1.675092422	1.55E-20	4.57E-20	down
YAE1	-1.674923498	2.42E-21	7.28E-21	down
ATG2B	-1.674709032	1.20E-53	6.92E-53	down
FANCL	-1.674427739	1.21E-46	6.28E-46	down
KRAS	-1.674380236	4.83E-104	5.74E-103	down
ARL5A	-1.673447388	2.03E-142	3.69E-141	down
ANKRD49	-1.672494141	3.44E-27	1.18E-26	down
DTWD2	-1.670976314	1.29E-30	4.81E-30	down
EFCAB13	-1.670976314	0.00670762	0.00987709	down
NPEPL1	-1.670976314	0.00089917	0.00142438	down
TMEM17	-1.670976314	1.82E-05	3.20E-05	down
ZNF222	-1.670976314	2.66E-06	4.89E-06	down
PHF10	-1.670508589	2.82E-166	6.65E-165	down
KCTD12	-1.670208718	4.25E-152	8.57E-151	down
MTBP	-1.670090953	2.83E-45	1.42E-44	down
SKIL	-1.669561213	1.05E-164	2.42E-163	down
PATJ	-1.669051437	1.02E-61	6.79E-61	down
POLQ	-1.668883935	6.53E-57	3.99E-56	down
MSMO1	-1.668521422	9.83E-174	2.49E-172	down
ALCAM	-1.667739057	9.10E-156	1.92E-154	down
CRYZL1	-1.666990966	2.77E-40	1.27E-39	down
RBAK	-1.666222784	4.27E-42	2.02E-41	down
ANKRD36C	-1.665814608	5.44E-09	1.13E-08	down
FOXO1	-1.664901799	2.89E-33	1.14E-32	down
CDK6	-1.663921556	1.71E-250	7.45E-249	down
TMEM182	-1.662708698	7.72E-11	1.72E-10	down
SLC35B4	-1.662438302	1.19E-86	1.12E-85	down
GABPA	-1.661757784	1.86E-84	1.71E-83	down
DNAH14	-1.661681422	1.82E-30	6.76E-30	down
ETNK1	-1.660662805	2.04E-193	6.06E-192	down
NUDT13	-1.660634369	1.11E-12	2.65E-12	down
SLC36A4	-1.660105578	1.02E-99	1.15E-98	down
STK38L	-1.660100957	3.04E-68	2.24E-67	down
LYSMD3	-1.660038467	1.07E-88	1.04E-87	down
HOXB5	-1.659835756	7.61E-05	0.00012927	down
FBXO48	-1.659835756	7.26E-12	1.68E-11	down
WDR11	-1.659274905	6.33E-96	6.77E-95	down
INHBA	-1.657493084	6.86E-13	1.64E-12	down
MIB1	-1.656894372	1.02E-165	2.40E-164	down
RAB30	-1.655635767	6.87E-29	2.46E-28	down
RAB3IP	-1.655355333	1.22E-52	6.96E-52	down
GORAB	-1.654325073	1.42E-17	3.89E-17	down
IKZF2	-1.6539028	6.69E-06	1.21E-05	down
ZNF33B	-1.653622747	1.14E-25	3.76E-25	down
MRE11	-1.653458552	2.56E-122	3.76E-121	down
HAVCR1	-1.653165037	9.59E-129	1.51E-127	down

RASA1	-1.653109893	3.29E-86	3.08E-85	down
NUP107	-1.652260705	1.06E-176	2.72E-175	down
IKBIP	-1.650838668	1.68E-158	3.64E-157	down
FBXL3	-1.650594814	8.06E-131	1.29E-129	down
CA11	-1.650217753	4.60E-05	7.91E-05	down
IRAK4	-1.649853022	2.94E-46	1.51E-45	down
SLC25A40	-1.649642172	3.92E-58	2.45E-57	down
ZNF141	-1.649281242	5.19E-19	1.47E-18	down
RECQL	-1.648015165	1.39E-104	1.66E-103	down
WRB	-1.6478032	1.08E-48	5.77E-48	down
HACE1	-1.647706534	1.61E-27	5.57E-27	down
MBTD1	-1.647706534	5.55E-34	2.23E-33	down
ZFP30	-1.647215786	2.07E-66	1.48E-65	down
RNPC3	-1.64719893	1.56E-28	5.54E-28	down
CCDC186	-1.646314259	2.67E-47	1.40E-46	down
SMIM8	-1.64528967	9.34E-15	2.37E-14	down
HELB	-1.644504102	8.91E-16	2.32E-15	down
SPATA6	-1.644504102	1.27E-18	3.56E-18	down
CMPK1	-1.644268867	0	0	down
ARL15	-1.644209404	1.75E-71	1.35E-70	down
DCBLD2	-1.644106913	0	0	down
ULBP1	-1.642355636	5.49E-16	1.44E-15	down
ZNF180	-1.642121451	1.49E-27	5.18E-27	down
CGGBP1	-1.642083762	2.25E-238	9.10E-237	down
MAN1A1	-1.641829968	4.41E-40	2.00E-39	down
PRKCI	-1.641254157	3.72E-184	1.03E-182	down
CCNG1	-1.64113058	0	0	down
GMCL1	-1.640718492	7.98E-56	4.79E-55	down
CASC4	-1.640032606	1.01E-97	1.11E-96	down
HIBCH	-1.638361949	5.87E-45	2.92E-44	down
PLCB1	-1.638272149	2.08E-16	5.51E-16	down
EMSY	-1.637293187	4.14E-61	2.72E-60	down
ZNF514	-1.637028982	1.57E-12	3.70E-12	down
HOOK3	-1.636760598	5.32E-99	5.95E-98	down
ANKRD26	-1.636713489	1.43E-26	4.84E-26	down
GPD2	-1.636091862	2.28E-121	3.34E-120	down
SLC38A9	-1.635487229	8.31E-29	2.97E-28	down
C11orf45	-1.634450438	0.00234488	0.00360177	down
IFIT3	-1.634450438	0.00019416	0.00032167	down
PPM1N	-1.634450438	0.0314373	0.04305767	down
RAG1	-1.634450438	0.0314373	0.04305767	down
TAC4	-1.634450438	0.0314373	0.04305767	down
THAP2	-1.634450438	1.69E-05	2.97E-05	down
TMEM139	-1.634450438	0.0314373	0.04305767	down
MLH3	-1.633589898	2.75E-34	1.11E-33	down
LRRC8B	-1.633542987	3.10E-94	3.25E-93	down
ARV1	-1.63174115	2.46E-22	7.57E-22	down
DNAJC18	-1.631447949	6.66E-11	1.49E-10	down
FAM83B	-1.631447949	6.66E-11	1.49E-10	down
SGO2	-1.631286835	1.57E-151	3.14E-150	down
ZDHHC17	-1.630787223	1.85E-47	9.67E-47	down
ADAMTS5	-1.630004533	1.64E-20	4.82E-20	down
IPMK	-1.629753651	2.35E-37	1.01E-36	down
ATP11C	-1.629700038	6.32E-119	8.98E-118	down

MOB1B	-1.629518142	3.76E-75	3.09E-74	down
INTS2	-1.629307134	3.98E-72	3.12E-71	down
DDX58	-1.629111845	1.45E-121	2.13E-120	down
SLFN11	-1.628033696	1.17E-105	1.42E-104	down
IDH1	-1.626733476	2.69E-224	9.81E-223	down
PTPN4	-1.626307264	1.19E-42	5.70E-42	down
TMEM117	-1.625888424	5.08E-08	1.01E-07	down
ZNF273	-1.625888424	2.51E-11	5.69E-11	down
ZNF559	-1.625172624	1.37E-13	3.36E-13	down
ZFYVE16	-1.622525571	6.79E-136	1.15E-134	down
CCDC122	-1.622066713	6.19E-06	1.12E-05	down
MTX3	-1.622066713	3.44E-35	1.42E-34	down
SRBD1	-1.622066713	1.43E-48	7.68E-48	down
ZNF619	-1.622066713	6.19E-06	1.12E-05	down
FAM76B	-1.621542954	8.87E-49	4.76E-48	down
SMIM15	-1.621020006	6.81E-131	1.10E-129	down
ZNF224	-1.620852834	3.74E-32	1.44E-31	down
HSPA4L	-1.620256556	1.19E-69	8.94E-69	down
PDP1	-1.619529548	3.56E-79	3.09E-78	down
ZNF567	-1.619237344	2.04E-23	6.43E-23	down
POLR3G	-1.618508894	6.39E-57	3.91E-56	down
FGF2	-1.618128687	5.12E-64	3.55E-63	down
WWP1	-1.6178446	3.29E-132	5.35E-131	down
DNAJC27	-1.617537055	1.07E-09	2.30E-09	down
ABHD13	-1.617037506	2.55E-55	1.51E-54	down
LMBRD2	-1.616489726	6.06E-72	4.74E-71	down
CD46	-1.616434479	0	0	down
VPS54	-1.616369983	4.92E-79	4.26E-78	down
WASHC4	-1.616198021	1.40E-178	3.69E-177	down
STK26	-1.616003545	7.77E-165	1.80E-163	down
DHX36	-1.61564924	1.03E-100	1.18E-99	down
TMEM41B	-1.615521709	2.32E-104	2.78E-103	down
VPS13C	-1.61543606	7.78E-94	8.07E-93	down
C7orf31	-1.615188535	1.16E-08	2.38E-08	down
MOB4	-1.614608499	3.89E-54	2.27E-53	down
NR2C1	-1.613548034	7.36E-62	4.92E-61	down
ASB3	-1.612082625	0.01811079	0.02555025	down
ZNF879	-1.612082625	0.00083094	0.00131892	down
CCDC88A	-1.611649057	1.72E-254	7.64E-253	down
PCM1	-1.611626388	5.49E-146	1.04E-144	down
RIF1	-1.610842663	0	0	down
SCML1	-1.610313532	1.58E-26	5.32E-26	down
CENPJ	-1.610222283	6.48E-49	3.49E-48	down
MYO6	-1.608970013	3.08E-142	5.58E-141	down
CSGALNACT1	-1.606436061	3.10E-13	7.51E-13	down
ANKRD6	-1.606436061	3.84E-17	1.04E-16	down
RANBP6	-1.606125169	1.07E-23	3.40E-23	down
ZNF616	-1.605778048	1.80E-22	5.55E-22	down
C9orf72	-1.605523252	1.16E-31	4.41E-31	down
FKBP14	-1.605205618	3.95E-46	2.03E-45	down
FAM117B	-1.604258408	8.97E-27	3.05E-26	down
FAM199X	-1.604146954	0	0	down
YES1	-1.603648528	4.45E-187	1.27E-185	down
ZNF93	-1.603469494	5.53E-27	1.89E-26	down

MDM2	-1.603104201	1.42E-262	6.72E-261	down
ICA1L	-1.602389228	2.86E-08	5.78E-08	down
CRIPT	-1.601773525	1.19E-39	5.37E-39	down
ZNF518B	-1.600966592	4.80E-29	1.72E-28	down
SETDB2	-1.600586986	9.36E-17	2.50E-16	down
BBX	-1.598435211	2.30E-150	4.56E-149	down
BMPR2	-1.597981095	3.09E-217	1.09E-215	down
LMBRD1	-1.59734973	4.23E-50	2.33E-49	down
MTF2	-1.597194538	2.56E-84	2.35E-83	down
BANK1	-1.596975732	0.01052914	0.0152395	down
BRSK1	-1.596975732	0.00029748	0.00048596	down
C15orf65	-1.596975732	0.01052914	0.0152395	down
CREB5	-1.596975732	5.26E-26	1.75E-25	down
IFIT2	-1.596975732	0.01052914	0.0152395	down
SLC18A2	-1.596975732	0.01052914	0.0152395	down
TRIM59	-1.596451973	3.10E-36	1.31E-35	down
PHC3	-1.596151947	3.33E-111	4.35E-110	down
METTL21A	-1.595788817	3.74E-32	1.44E-31	down
ASPM	-1.595771165	0	0	down
ANXA1	-1.594840546	5.54E-288	2.96E-286	down
TTC30A	-1.592973802	5.70E-06	1.03E-05	down
CPNE8	-1.592809101	1.31E-139	2.31E-138	down
BLOC1S6	-1.592146623	5.87E-91	5.87E-90	down
AGPS	-1.592057249	8.52E-206	2.75E-204	down
GPATCH2	-1.591668448	3.44E-32	1.33E-31	down
USP37	-1.591511768	1.84E-44	9.13E-44	down
ATP2B1	-1.591287425	1.35E-102	1.57E-101	down
GBP3	-1.590357853	1.85E-12	4.37E-12	down
TRIM5	-1.589794557	1.13E-26	3.85E-26	down
PLEKHA1	-1.589694393	7.28E-83	6.58E-82	down
JMY	-1.589565188	2.58E-28	9.13E-28	down
SCYL3	-1.589020718	2.00E-17	5.48E-17	down
CYP51A1	-1.588282818	0	0	down
GOLGB1	-1.586997921	1.18E-119	1.69E-118	down
TBC1D15	-1.586717827	9.08E-75	7.40E-74	down
FER	-1.586087416	1.10E-60	7.18E-60	down
NEK7	-1.586087416	7.48E-180	2.00E-178	down
SLC10A7	-1.586087416	4.31E-08	8.62E-08	down
TINAG	-1.586087416	2.74E-26	9.20E-26	down
ATF2	-1.585468632	2.02E-125	3.07E-124	down
PHTF2	-1.584894613	1.48E-91	1.49E-90	down
TTBK2	-1.584775279	9.20E-37	3.91E-36	down
ORC2	-1.582953846	3.61E-99	4.04E-98	down
RWDD4	-1.582749131	3.44E-29	1.24E-28	down
UBE2W	-1.581055729	2.85E-56	1.73E-55	down
SSX2IP	-1.580621995	1.50E-132	2.47E-131	down
ARL6	-1.580612286	3.52E-21	1.05E-20	down
DCP2	-1.579684634	1.71E-82	1.54E-81	down
ZNF84	-1.579636002	1.74E-39	7.82E-39	down
DMXL2	-1.579137447	2.96E-54	1.73E-53	down
UGP2	-1.578888747	4.33E-145	8.15E-144	down
SLC35G1	-1.578294311	6.28E-33	2.46E-32	down
10-9月	-1.577989426	1.02E-218	3.61E-217	down
AMPD3	-1.577866909	0.00362963	0.00547493	down

NIPSNAP3B	-1.577866909	0.00362963	0.00547493	down
PBX1	-1.577866909	0.00362963	0.00547493	down
SOCS5	-1.577187834	2.97E-158	6.41E-157	down
CAB39L	-1.575556749	1.02E-24	3.30E-24	down
TWF1	-1.575272812	0	0	down
ADAM22	-1.574468333	3.86E-25	1.26E-24	down
UBE2Q2	-1.574349977	1.54E-54	9.07E-54	down
MAGEE1	-1.573421004	1.74E-07	3.41E-07	down
PIGN	-1.573180294	2.34E-103	2.75E-102	down
SNX14	-1.572156085	1.07E-149	2.12E-148	down
PM20D2	-1.572084557	1.27E-129	2.01E-128	down
TMEM65	-1.57183846	6.04E-61	3.96E-60	down
PCGF5	-1.571587846	1.13E-81	1.00E-80	down
TAB3	-1.571574118	7.05E-90	6.95E-89	down
APPL1	-1.571561443	3.20E-196	9.63E-195	down
AL157392.5	-1.57144064	0.00214681	0.0033083	down
RIDA	-1.57144064	1.83E-28	6.51E-28	down
ANKMY2	-1.570812152	3.53E-39	1.58E-38	down
MAP3K21	-1.5703201	3.26E-33	1.28E-32	down
PARP14	-1.568286396	6.43E-65	4.51E-64	down
MFN1	-1.567546724	6.62E-110	8.47E-109	down
ESCO2	-1.567336242	1.20E-61	7.95E-61	down
ZNF512	-1.56659124	1.61E-35	6.67E-35	down
PHF21A	-1.56650159	4.07E-49	2.20E-48	down
LOX	-1.566278935	5.21E-06	9.45E-06	down
SRP9	-1.566053002	0	0	down
LRRC8C	-1.565042869	2.37E-78	2.03E-77	down
SOX4	-1.56477691	9.92E-23	3.08E-22	down
MAP4K5	-1.564297211	9.22E-108	1.15E-106	down
ZBTB6	-1.564297211	3.31E-44	1.63E-43	down
LMAN1	-1.562812327	0	0	down
TXNDC16	-1.562600153	5.60E-36	2.35E-35	down
ANKRD36B	-1.562041942	5.47E-09	1.14E-08	down
PCNP	-1.561902126	4.40E-274	2.20E-272	down
PGGT1B	-1.561098696	4.53E-32	1.74E-31	down
EXOC5	-1.56058541	1.20E-223	4.37E-222	down
DEK	-1.560247411	0	0	down
ZNF14	-1.560195883	6.17E-12	1.43E-11	down
ZBED3	-1.559330958	9.07E-23	2.82E-22	down
ZNF248	-1.559330958	3.78E-12	8.82E-12	down
RBMS1	-1.559244737	8.77E-131	1.41E-129	down
SCAF11	-1.558975859	5.01E-277	2.55E-275	down
ZNF714	-1.558649834	1.38E-60	9.00E-60	down
FAXC	-1.558501584	0.0004534	0.00073279	down
RAB18	-1.557824149	1.01E-91	1.02E-90	down
ZNF155	-1.557705615	1.42E-12	3.37E-12	down
RND3	-1.556126219	2.34E-27	8.09E-27	down
FAM208A	-1.556082278	9.33E-167	2.23E-165	down
ARL4A	-1.555753069	2.28E-33	9.04E-33	down
PPP1CB	-1.555729016	1.24E-245	5.25E-244	down
ANK3	-1.555499096	0.00027143	0.00044523	down
ZNF720	-1.555499096	2.85E-10	6.23E-10	down
BROX	-1.554487507	3.48E-144	6.47E-143	down
ARL17B	-1.554162649	1.59E-07	3.11E-07	down

APC	-1.553578266	6.39E-81	5.63E-80	down
FGD4	-1.553406717	5.55E-17	1.49E-16	down
TBC1D31	-1.552249375	4.88E-46	2.49E-45	down
CSNK1G3	-1.552070908	1.14E-71	8.86E-71	down
ZNF638	-1.551495512	6.99E-189	2.01E-187	down
ZNF354A	-1.551105841	1.86E-34	7.57E-34	down
DSC3	-1.550967169	4.15E-123	6.15E-122	down
ST3GAL6	-1.55068208	3.60E-08	7.23E-08	down
CARD8	-1.550422859	1.90E-28	6.75E-28	down
RBM45	-1.549475309	6.98E-19	1.97E-18	down
PLEKHA5	-1.549058946	4.43E-40	2.01E-39	down
KLHL23	-1.549048884	5.43E-61	3.56E-60	down
ZKSCAN3	-1.547828083	8.17E-09	1.69E-08	down
BBIP1	-1.547542372	1.30E-12	3.08E-12	down
TP53INP1	-1.547542372	1.30E-12	3.08E-12	down
ALKBH8	-1.546693978	3.18E-43	1.54E-42	down
RB1	-1.546474912	2.76E-191	8.02E-190	down
ZNF493	-1.546453957	4.88E-13	1.17E-12	down
TRMT5	-1.545761431	2.68E-56	1.63E-55	down
KIAA1841	-1.545183099	1.58E-45	7.96E-45	down
EIF2AK3	-1.545125508	1.21E-37	5.25E-37	down
SOS1	-1.54500451	8.66E-106	1.05E-104	down
CCNT2	-1.544993105	7.47E-78	6.37E-77	down
RALGAPA1	-1.544284309	2.00E-26	6.74E-26	down
ESRP1	-1.544064201	1.30E-05	2.30E-05	down
MSH2	-1.543515404	3.32E-268	1.61E-266	down
LPGAT1	-1.54343846	3.57E-218	1.26E-216	down
ZNF532	-1.543288881	9.93E-79	8.55E-78	down
PHYH	-1.542819962	3.92E-19	1.11E-18	down
RARG	-1.542819962	9.81E-15	2.48E-14	down
ARID4A	-1.542527948	4.70E-37	2.01E-36	down
DGKH	-1.542486815	1.70E-94	1.79E-93	down
ARL13B	-1.541287806	3.38E-62	2.27E-61	down
FAM122B	-1.540357091	9.50E-152	1.91E-150	down
ACADM	-1.540147243	1.40E-55	8.35E-55	down
SLF1	-1.540034836	5.00E-46	2.55E-45	down
ARRDC4	-1.53973178	1.74E-06	3.24E-06	down
CEP83	-1.539554098	4.62E-52	2.61E-51	down
UBA5	-1.539254251	6.97E-58	4.33E-57	down
TAOK1	-1.538720747	2.00E-226	7.44E-225	down
ST3GAL5	-1.538082043	6.43E-07	1.22E-06	down
FNDC3A	-1.537956324	3.44E-132	5.59E-131	down
GK	-1.537529039	5.57E-24	1.78E-23	down
DHX40	-1.536340352	2.97E-126	4.54E-125	down
ANAPC4	-1.536133771	8.37E-44	4.10E-43	down
SKIDA1	-1.536046734	1.45E-07	2.84E-07	down
MRPS36	-1.535748933	2.70E-26	9.05E-26	down
C17orf75	-1.535092886	7.32E-21	2.17E-20	down
ODR4	-1.535021161	8.10E-34	3.24E-33	down
ALDH6A1	-1.534403261	5.48E-15	1.40E-14	down
ZNF195	-1.534403261	4.46E-35	1.84E-34	down
ACVR2B	-1.533694726	1.27E-15	3.30E-15	down
TMEM218	-1.53347279	5.72E-23	1.79E-22	down
MED23	-1.533167102	2.16E-52	1.22E-51	down

KIF14	-1.533125111	3.20E-197	9.72E-196	down
ITGAV	-1.533078509	0	0	down
RCN1	-1.531940187	5.67E-199	1.74E-197	down
SGMS2	-1.530655025	2.56E-54	1.50E-53	down
COMMD2	-1.529422169	3.50E-70	2.65E-69	down
BBS7	-1.528821006	7.52E-33	2.95E-32	down
ANK2	-1.528371918	1.75E-12	4.14E-12	down
TRMT61B	-1.52819175	1.07E-12	2.56E-12	down
PDCD4	-1.52738546	9.32E-14	2.29E-13	down
MFSD8	-1.527101101	8.48E-27	2.88E-26	down
MAP3K2	-1.526122368	7.84E-144	1.45E-142	down
LARP4	-1.525730586	5.84E-197	1.76E-195	down
TBC1D23	-1.525225429	9.48E-67	6.83E-66	down
CENPE	-1.524502913	5.66E-185	1.59E-183	down
TRIM38	-1.524386716	5.31E-58	3.30E-57	down
MFAP3L	-1.524248162	6.05E-21	1.80E-20	down
TMED5	-1.52408999	2.20E-219	7.81E-218	down
USP15	-1.523348341	2.36E-94	2.48E-93	down
MIGA1	-1.522504911	4.34E-59	2.75E-58	down
ZNF585B	-1.52226329	1.63E-32	6.37E-32	down
GNB4	-1.52137571	7.90E-128	1.22E-126	down
MIA2	-1.52137571	7.67E-44	3.77E-43	down
NCOA2	-1.520342651	1.89E-75	1.56E-74	down
MIER1	-1.519678177	1.27E-144	2.37E-143	down
DEPDC1	-1.519434661	8.46E-220	3.00E-218	down
ARPIN	-1.51897322	0.00194286	0.00300507	down
BST1	-1.51897322	0.02843703	0.03921162	down
C2orf15	-1.51897322	0.0055768	0.00827982	down
C8orf48	-1.51897322	0.00194286	0.00300507	down
CBLB	-1.51897322	4.52E-15	1.16E-14	down
CFAP69	-1.51897322	7.10E-06	1.28E-05	down
EDN1	-1.51897322	0.00014748	0.00024629	down
FAM131C	-1.51897322	0.00952347	0.01383496	down
GLDN	-1.51897322	0.02843703	0.03921162	down
HOXA7	-1.51897322	3.01E-11	6.79E-11	down
IFIT1	-1.51897322	0.00952347	0.01383496	down
MGST2	-1.51897322	0.02843703	0.03921162	down
MTERF1	-1.51897322	7.88E-22	2.39E-21	down
NIPAL1	-1.51897322	2.78E-15	7.15E-15	down
P2RX5-TAX1	-1.51897322	0.01637968	0.02319658	down
PRSS35	-1.51897322	0.00952347	0.01383496	down
RASGRF2	-1.51897322	0.01637968	0.02319658	down
RYR3	-1.51897322	0.02843703	0.03921162	down
VN1R1	-1.51897322	0.02843703	0.03921162	down
ZNF92	-1.51897322	1.13E-22	3.51E-22	down
ZNF99	-1.51897322	5.34E-05	9.15E-05	down
THUMPD1	-1.517929679	4.09E-98	4.51E-97	down
RAB3GAP2	-1.516347751	2.22E-116	3.07E-115	down
PI4K2B	-1.516037929	5.89E-36	2.47E-35	down
CAMK2D	-1.515458736	2.75E-30	1.02E-29	down
MMS22L	-1.515036806	3.96E-78	3.39E-77	down
MBTPS2	-1.513902231	1.89E-21	5.69E-21	down
CEP57L1	-1.513337685	2.94E-37	1.26E-36	down
TMEM64	-1.512474583	6.17E-94	6.42E-93	down

STXBP3	-1.512415504	4.23E-93	4.35E-92	down
COMMD8	-1.511777719	2.21E-43	1.08E-42	down
ZMYM4	-1.511233421	1.83E-130	2.93E-129	down
RAPGEF6	-1.511132467	3.54E-27	1.21E-26	down
DDIAS	-1.510958227	2.25E-76	1.89E-75	down
PPM1B	-1.510771407	1.60E-62	1.08E-61	down
C4orf46	-1.510633919	1.91E-73	1.53E-72	down
MTMR6	-1.509531465	4.60E-107	5.70E-106	down
ATR	-1.50932305	1.76E-94	1.85E-93	down
FBXL4	-1.508166465	2.54E-29	9.18E-29	down
CDC40	-1.508002102	5.68E-38	2.48E-37	down
TATDN3	-1.507745965	2.20E-19	6.29E-19	down
ZCCHC4	-1.506427967	1.75E-17	4.78E-17	down
SIKE1	-1.506345612	3.61E-127	5.57E-126	down
RBL1	-1.505862305	7.06E-100	7.97E-99	down
SNX16	-1.503866328	2.52E-21	7.56E-21	down
CNOT6L	-1.503590187	1.07E-46	5.55E-46	down
ACAP2	-1.503175878	3.19E-145	6.00E-144	down
FAM160B1	-1.502671408	4.66E-38	2.04E-37	down
PHF20L1	-1.502066444	1.43E-106	1.76E-105	down
CDK17	-1.501354368	2.26E-74	1.83E-73	down
IFT88	-1.500535972	4.70E-23	1.47E-22	down
PPIG	-1.499722741	2.44E-139	4.29E-138	down
RB1CC1	-1.499107914	8.93E-81	7.85E-80	down
HAT1	-1.499010725	1.63E-173	4.12E-172	down
ARPP19	-1.498381075	0	0	down
ZNF24	-1.49832891	1.24E-186	3.52E-185	down
TMTC4	-1.497911605	2.58E-20	7.56E-20	down
GNAQ	-1.497845509	1.12E-66	8.07E-66	down
ARL14EP	-1.497570743	4.18E-52	2.36E-51	down
C21orf91	-1.497328226	1.45E-60	9.43E-60	down
B3GALNT1	-1.497113786	1.69E-55	1.01E-54	down
TRDMT1	-1.496946914	1.75E-10	3.86E-10	down
SETX	-1.495574568	0	0	down
VPS41	-1.49542917	1.20E-113	1.61E-112	down
RLIM	-1.494999369	4.29E-217	1.50E-215	down
SLC9A2	-1.494725674	1.75E-05	3.09E-05	down
SHOC2	-1.494478658	1.73E-61	1.14E-60	down
ZDHHC13	-1.494098552	5.87E-41	2.71E-40	down
DNAJB9	-1.4936065	1.77E-32	6.88E-32	down
NFXL1	-1.493573003	4.05E-40	1.84E-39	down
U2SURP	-1.493438128	2.45E-264	1.18E-262	down
TMEM87B	-1.493055846	2.32E-69	1.74E-68	down
PDCD10	-1.49257553	1.63E-97	1.78E-96	down
CYYR1	-1.492501009	7.84E-35	3.21E-34	down
MYSM1	-1.492431433	2.74E-104	3.27E-103	down
ARHGAP12	-1.49230527	3.12E-38	1.37E-37	down
KANSL1L	-1.491622732	7.02E-16	1.84E-15	down
NEMF	-1.491155276	1.58E-78	1.36E-77	down
MTMR2	-1.489463338	3.44E-136	5.85E-135	down
ZNF558	-1.488790215	7.93E-34	3.18E-33	down
TNFSF18	-1.488599571	3.63E-11	8.17E-11	down
ZNF354B	-1.488599571	2.13E-14	5.34E-14	down
BLZF1	-1.488041361	1.55E-45	7.85E-45	down

TEFM	-1.487812289	3.42E-17	9.25E-17	down
TTK	-1.487508451	3.89E-158	8.37E-157	down
KIAA2026	-1.487089159	9.22E-14	2.26E-13	down
FAM227A	-1.486551742	0.00022122	0.0003649	down
MPHOSPH9	-1.486551742	6.07E-97	6.58E-96	down
SENP6	-1.486191949	3.91E-128	6.10E-127	down
ORC3	-1.486033546	1.53E-89	1.50E-88	down
DICER1	-1.485901399	1.02E-71	7.97E-71	down
ABCC4	-1.485856282	4.90E-179	1.30E-177	down
MOB1A	-1.485838772	0	0	down
BRMS1L	-1.485614623	1.96E-36	8.32E-36	down
CD58	-1.485313909	3.05E-33	1.20E-32	down
NUBPL	-1.485252602	1.03E-21	3.13E-21	down
EOGT	-1.485217621	1.76E-84	1.62E-83	down
TCEAL1	-1.48512545	1.71E-24	5.51E-24	down
SPOPL	-1.484645756	1.94E-49	1.06E-48	down
ZBTB1	-1.484415998	1.40E-68	1.04E-67	down
TIMM9	-1.483926273	1.97E-26	6.64E-26	down
ECHDC1	-1.483697745	1.76E-83	1.60E-82	down
AADAT	-1.482709676	3.08E-20	9.01E-20	down
CROT	-1.482709676	3.08E-20	9.01E-20	down
ZNF568	-1.482631373	7.25E-15	1.84E-14	down
C12orf4	-1.482501663	1.66E-46	8.57E-46	down
TMEM126B	-1.482354629	3.55E-54	2.07E-53	down
TTC37	-1.481808827	4.85E-146	9.22E-145	down
ACTR6	-1.481705962	8.83E-38	3.84E-37	down
TSPAN31	-1.481498515	7.52E-12	1.74E-11	down
TLK1	-1.480370381	2.10E-140	3.73E-139	down
YOD1	-1.480175721	3.18E-41	1.48E-40	down
HOXD10	-1.479979089	2.12E-06	3.92E-06	down
SLC22A15	-1.479979089	2.12E-06	3.92E-06	down
PCGF6	-1.479660296	4.68E-26	1.56E-25	down
TWSG1	-1.479660296	4.54E-208	1.49E-206	down
ELOVL7	-1.47926351	2.41E-88	2.32E-87	down
SCFD1	-1.478833829	5.38E-66	3.84E-65	down
MON2	-1.47872005	9.51E-85	8.76E-84	down
NCKAP1	-1.478654674	0	0	down
FCHO2	-1.478544794	1.51E-39	6.79E-39	down
TRERF1	-1.478544794	1.51E-39	6.79E-39	down
EHD3	-1.478331236	0.00103591	0.00163499	down
GSDMB	-1.478331236	0.00103591	0.00163499	down
METTL18	-1.478331236	3.49E-06	6.40E-06	down
EHBP1	-1.478268685	1.51E-130	2.42E-129	down
ZNF286A	-1.477750557	1.51E-15	3.93E-15	down
USP48	-1.477532418	1.00E-132	1.65E-131	down
ESCO1	-1.477346125	5.28E-59	3.35E-58	down
DOCK10	-1.477066268	1.38E-33	5.49E-33	down
RMDN1	-1.47704254	9.37E-52	5.27E-51	down
LIG4	-1.476647811	5.19E-40	2.35E-39	down
GDAP1	-1.476537954	3.66E-24	1.17E-23	down
UACA	-1.476537954	5.68E-87	5.36E-86	down
CAPRIN2	-1.476481138	1.13E-75	9.39E-75	down
TMOD2	-1.476328883	8.40E-22	2.55E-21	down
RANBP17	-1.475720022	4.51E-17	1.22E-16	down

MAN1A2	-1.475675153	9.46E-138	1.63E-136	down
IPO11	-1.474898246	1.41E-111	1.85E-110	down
PAN3	-1.473083822	5.91E-72	4.62E-71	down
KYAT3	-1.472815774	2.82E-61	1.86E-60	down
ADGRG6	-1.471930381	0	0	down
TMEM168	-1.471372187	3.17E-75	2.60E-74	down
SENP1	-1.471174881	5.90E-71	4.55E-70	down
PHACTR2	-1.471088989	2.90E-39	1.30E-38	down
ZC3H12C	-1.470764127	6.46E-43	3.11E-42	down
TMEM263	-1.470173922	9.45E-89	9.19E-88	down
NPHP3	-1.47006362	0.00294193	0.00447786	down
SEC31B	-1.47006362	2.60E-05	4.54E-05	down
GMFB	-1.469793224	3.01E-214	1.04E-212	down
SEC24A	-1.469793224	1.02E-72	8.05E-72	down
PPFIBP1	-1.46916292	7.17E-87	6.77E-86	down
HS2ST1	-1.468902065	6.33E-109	7.98E-108	down
ZNF432	-1.468787463	8.67E-32	3.32E-31	down
RBM12B	-1.468347147	8.03E-41	3.70E-40	down
CASP8AP2	-1.467933787	1.21E-73	9.66E-73	down
SEL1L	-1.467834371	4.56E-156	9.63E-155	down
GCFC2	-1.467544981	3.82E-51	2.13E-50	down
ADGRV1	-1.46744292	4.32E-05	7.43E-05	down
SNX13	-1.467190678	1.15E-61	7.63E-61	down
PRPF40A	-1.466264076	0	0	down
PAQR3	-1.466166861	2.30E-92	2.34E-91	down
UTRN	-1.465502012	1.29E-164	2.98E-163	down
CREBL2	-1.464525436	1.31E-39	5.89E-39	down
NR3C2	-1.464525436	0.0049879	0.00742927	down
RAD21L1	-1.464525436	0.0049879	0.00742927	down
ATE1	-1.464198998	1.06E-66	7.60E-66	down
C5orf34	-1.464198998	3.11E-34	1.26E-33	down
PRKAA1	-1.46367933	0	0	down
EMC2	-1.463004408	1.62E-43	7.88E-43	down
CHM	-1.462516238	1.15E-59	7.34E-59	down
7-9月	-1.462460065	0	0	down
OPA1	-1.462316827	1.92E-202	6.08E-201	down
AASDH	-1.462110883	8.60E-15	2.18E-14	down
AGGF1	-1.461829313	2.62E-21	7.87E-21	down
USO1	-1.461305133	1.13E-119	1.61E-118	down
SPIN2B	-1.461257722	0.000119	0.00020001	down
ZFP62	-1.461257722	2.64E-11	5.99E-11	down
ERCC6L	-1.460581425	1.22E-92	1.25E-91	down
THUMPD2	-1.460447144	2.09E-22	6.43E-22	down
VEZF1	-1.460309828	2.88E-84	2.64E-83	down
LRRC37A	-1.460079531	3.13E-06	5.75E-06	down
PHKB	-1.460058055	2.04E-178	5.34E-177	down
IL6ST	-1.459476687	0	0	down
CPLANE1	-1.45941277	1.66E-98	1.84E-97	down
ZNF12	-1.459330274	2.62E-42	1.24E-41	down
RFXAP	-1.458431678	1.79E-15	4.63E-15	down
NICN1	-1.457572675	0.00850107	0.01240056	down
ZKSCAN4	-1.457572675	0.00019805	0.0003279	down
ZBTB21	-1.4573102	6.89E-47	3.57E-46	down
YAP1	-1.457080624	0	0	down

7-3月	-1.456569185	8.62E-202	2.71E-200	down
QKI	-1.45625782	3.05E-199	9.36E-198	down
INSIG1	-1.454658407	5.55E-224	2.02E-222	down
LTV1	-1.454524512	1.81E-40	8.25E-40	down
MEIS1	-1.453384879	0.00033022	0.00053842	down
RAB33B	-1.45288403	3.33E-14	8.30E-14	down
TDRD3	-1.452739522	3.43E-21	1.03E-20	down
TUT4	-1.45028564	1.25E-88	1.22E-87	down
ZNF283	-1.449367753	7.93E-19	2.24E-18	down
POLE2	-1.449222112	7.65E-44	3.75E-43	down
VCAN	-1.44918489	9.09E-69	6.76E-68	down
REEP3	-1.448937451	1.59E-115	2.17E-114	down
CBR4	-1.448583892	2.34E-13	5.69E-13	down
CCDC148	-1.448583892	0.00055175	0.0008877	down
ERCC8	-1.448583892	1.08E-31	4.12E-31	down
INPP4B	-1.448583892	2.33E-05	4.08E-05	down
PDK1	-1.448583892	1.80E-26	6.07E-26	down
SLC16A14	-1.448583892	0.01458399	0.02075615	down
MIPOL1	-1.446961975	3.40E-18	9.43E-18	down
SNX4	-1.446632986	9.01E-50	4.93E-49	down
FIG4	-1.44646072	1.32E-20	3.89E-20	down
SOCS6	-1.446146907	1.17E-45	5.93E-45	down
ZXDB	-1.445763373	2.02E-25	6.65E-25	down
LMLN	-1.445350357	4.86E-14	1.21E-13	down
SMCHD1	-1.445097315	4.91E-189	1.41E-187	down
TMEM123	-1.445043535	0	0	down
CFAP36	-1.444886252	6.61E-56	3.97E-55	down
TMEM62	-1.444495004	2.76E-21	8.28E-21	down
SC5D	-1.444479494	2.17E-115	2.96E-114	down
ALKBH3	-1.443871765	4.49E-10	9.75E-10	down
MFAP3	-1.443871765	1.06E-35	4.42E-35	down
UBE2V1	-1.443024367	0.00092412	0.00146335	down
RBM7	-1.44180536	4.73E-37	2.02E-36	down
TTC21B	-1.441494467	1.30E-48	6.97E-48	down
KNL1	-1.441368951	1.18E-161	2.62E-160	down
GLCCI1	-1.440970708	1.57E-08	3.21E-08	down
TGFBR1	-1.439796634	1.26E-72	9.92E-72	down
ARMT1	-1.439368826	1.23E-50	6.84E-50	down
HIGD1A	-1.439321506	4.03E-200	1.24E-198	down
IKZF4	-1.439004915	4.35E-14	1.08E-13	down
AGO4	-1.43835476	1.68E-18	4.70E-18	down
NEBL	-1.438053225	1.17E-11	2.67E-11	down
RICTOR	-1.437154804	8.91E-122	1.31E-120	down
CTSF	-1.43651106	0.02523237	0.03494387	down
ZNF383	-1.43651106	1.15E-13	2.82E-13	down
ABCA5	-1.435588326	3.24E-35	1.34E-34	down
KBTBD6	-1.434862265	3.57E-39	1.60E-38	down
UTP14C	-1.433364505	2.41E-16	6.37E-16	down
XPO1	-1.43322601	0	0	down
TMEM68	-1.433112483	3.06E-15	7.86E-15	down
PLAG1	-1.432816576	1.26E-05	2.24E-05	down
ZNF451	-1.432667856	1.17E-109	1.49E-108	down
CWF19L2	-1.43264842	1.30E-25	4.30E-25	down
GTPBP8	-1.432558468	3.09E-17	8.37E-17	down

CLOCK	-1.432211908	3.18E-48	1.70E-47	down
FAM114A2	-1.431510379	2.28E-29	8.25E-29	down
SLC30A9	-1.431444028	1.69E-83	1.54E-82	down
OCIAD1	-1.431344313	2.87E-132	4.68E-131	down
NAA16	-1.431244371	1.50E-42	7.16E-42	down
USP1	-1.431114375	2.69E-302	1.53E-300	down
NRAS	-1.429286143	0	0	down
IQCG	-1.428775411	2.08E-05	3.65E-05	down
ZNF670	-1.428775411	1.68E-13	4.09E-13	down
ZNF782	-1.428775411	1.86E-07	3.63E-07	down
RNF138	-1.428468531	1.96E-83	1.78E-82	down
NSL1	-1.427257051	9.87E-35	4.03E-34	down
SLC19A2	-1.427180744	6.67E-28	2.34E-27	down
ZNF658	-1.427050731	2.77E-11	6.27E-11	down
ELK4	-1.426838107	1.43E-113	1.92E-112	down
PANK3	-1.426697847	4.98E-194	1.48E-192	down
ZNF90	-1.426609202	2.86E-09	6.03E-09	down
FBXO30	-1.426407101	4.24E-65	2.99E-64	down
COG3	-1.426216079	2.59E-34	1.05E-33	down
OCLN	-1.425863816	0.00029394	0.00048049	down
ZNF529	-1.4256695	3.08E-31	1.16E-30	down
DEPDC7	-1.424997072	7.22E-15	1.84E-14	down
PTER	-1.424609532	1.10E-33	4.37E-33	down
ZBTB18	-1.424469234	1.43E-34	5.82E-34	down
CEP135	-1.42423273	7.76E-44	3.81E-43	down
ADAM10	-1.423534403	2.43E-229	9.26E-228	down
ZNF846	-1.42339556	4.12E-06	7.51E-06	down
PPARG	-1.423289872	4.87E-35	2.00E-34	down
ZNF816	-1.422946332	2.47E-17	6.72E-17	down
IFIT5	-1.422897249	6.96E-23	2.17E-22	down
TAB2	-1.422757905	2.19E-117	3.08E-116	down
SEC62	-1.421307837	9.28E-158	1.99E-156	down
RAP2C	-1.420905441	2.39E-76	2.00E-75	down
REV3L	-1.420077465	9.59E-59	6.05E-58	down
FEM1B	-1.419753615	2.03E-59	1.29E-58	down
ABLIM1	-1.419437547	2.47E-11	5.61E-11	down
COX16	-1.419437547	6.79E-06	1.22E-05	down
DCLRE1C	-1.419437547	2.47E-11	5.61E-11	down
GCNT7	-1.419437547	0.04415638	0.0592999	down
ITGA2B	-1.419437547	0.04415638	0.0592999	down
SEMA4G	-1.419437547	0.00049042	0.00079103	down
VCPIP1	-1.419437547	4.47E-45	2.24E-44	down
USF3	-1.41897743	2.49E-41	1.16E-40	down
SPIN4	-1.418317007	7.07E-83	6.39E-82	down
C3orf58	-1.416572213	2.05E-33	8.14E-33	down
PEX11A	-1.415879727	1.35E-06	2.53E-06	down
SLC41A2	-1.415615836	1.95E-25	6.42E-25	down
SYT11	-1.415137409	5.22E-10	1.13E-09	down
MAPK6	-1.415022256	2.37E-67	1.72E-66	down
TTC14	-1.414950156	4.20E-42	1.98E-41	down
ZCCHC10	-1.414864817	1.88E-29	6.83E-29	down
CDK14	-1.414003661	3.35E-08	6.74E-08	down
SEMA3C	-1.413648201	4.33E-229	1.64E-227	down
GIN1	-1.412844869	8.52E-10	1.83E-09	down

ATL1	-1.412058016	0.00082004	0.00130209	down
PRRG1	-1.411696484	2.68E-55	1.59E-54	down
HSD17B12	-1.41102306	2.56E-281	1.32E-279	down
PALLD	-1.411013972	2.54E-57	1.56E-56	down
PLOD2	-1.410924759	2.14E-166	5.05E-165	down
TRMT1L	-1.410109744	5.06E-48	2.68E-47	down
PIK3R1	-1.410038849	1.95E-30	7.23E-30	down
GOLGA8B	-1.409972443	2.01E-26	6.77E-26	down
TYW5	-1.409880363	2.11E-22	6.50E-22	down
TERF1	-1.409522078	1.73E-52	9.82E-52	down
ZNF675	-1.409000378	1.27E-20	3.74E-20	down
USP16	-1.408747658	7.38E-64	5.10E-63	down
PAFAH2	-1.408612773	1.37E-16	3.63E-16	down
MTDH	-1.408312863	1.33E-297	7.42E-296	down
ABRAXAS1	-1.407941908	2.27E-09	4.81E-09	down
TYW3	-1.407689887	2.04E-41	9.51E-41	down
SPRED1	-1.407635733	5.06E-67	3.66E-66	down
ZYG11B	-1.407112194	5.92E-86	5.53E-85	down
TENT5A	-1.40708034	3.16E-13	7.63E-13	down
C2orf49	-1.406430441	9.34E-35	3.82E-34	down
PTBP2	-1.405316438	1.73E-24	5.59E-24	down
GOPC	-1.404461339	1.23E-44	6.13E-44	down
LNPEP	-1.404231134	2.70E-170	6.67E-169	down
ADAMTS9	-1.40395656	1.22E-16	3.24E-16	down
CCDC171	-1.403496003	2.98E-08	6.02E-08	down
KLHL9	-1.403363736	7.89E-54	4.59E-53	down
TXNDC9	-1.403351321	2.31E-49	1.26E-48	down
C12orf29	-1.402979925	8.94E-42	4.20E-41	down
PBRM1	-1.402894481	1.87E-127	2.90E-126	down
RCOR3	-1.402780203	3.25E-40	1.48E-39	down
CYBRD1	-1.402563728	6.07E-09	1.26E-08	down
UPRT	-1.402563728	1.98E-16	5.24E-16	down
DACH1	-1.402159555	1.20E-06	2.26E-06	down
ZNF566	-1.402032675	3.24E-25	1.06E-24	down
CUL5	-1.401469135	1.45E-96	1.56E-95	down
HMBOX1	-1.40113673	2.42E-07	4.69E-07	down
ZNF397	-1.40113673	3.73E-19	1.06E-18	down
PAXBP1	-1.400113179	7.72E-89	7.52E-88	down
SLC35A5	-1.399878163	1.96E-43	9.52E-43	down
NFAT5	-1.399154545	0	0	down
TDG	-1.39902171	9.03E-54	5.24E-53	down
RAB11FIP2	-1.398773592	1.98E-39	8.87E-39	down
RBBP9	-1.398615433	8.07E-58	5.00E-57	down
ZFAND6	-1.398118825	5.83E-59	3.69E-58	down
KLF11	-1.397957819	1.76E-16	4.67E-16	down
PHF14	-1.397957819	5.52E-61	3.62E-60	down
SIAE	-1.397957819	1.76E-16	4.67E-16	down
ADD3	-1.39741124	1.18E-46	6.12E-46	down
C11orf58	-1.397346064	6.75E-167	1.62E-165	down
RNF219	-1.397095393	2.03E-44	1.01E-43	down
TWISTNB	-1.397090115	7.20E-69	5.36E-68	down
ARMCX5	-1.39688964	1.47E-27	5.10E-27	down
APLF	-1.396116472	6.75E-10	1.45E-09	down
PXYLP1	-1.396116472	6.75E-10	1.45E-09	down

CENPU	-1.395914824	1.09E-55	6.54E-55	down
KIF5C	-1.395914824	1.12E-19	3.24E-19	down
ALDH1L2	-1.395824989	2.35E-20	6.89E-20	down
TRNT1	-1.395626585	1.92E-42	9.15E-42	down
TMEM19	-1.395614527	4.15E-63	2.83E-62	down
IREB2	-1.395494745	1.78E-77	1.52E-76	down
SCAI	-1.394882872	1.47E-32	5.73E-32	down
FEM1C	-1.393968965	7.63E-44	3.74E-43	down
ZNF664	-1.393795176	9.12E-190	2.64E-188	down
AK7	-1.393442338	0.01284963	0.01841521	down
CDC26	-1.393442338	0.00231095	0.00355137	down
MCEE	-1.393442338	0.0004341	0.00070249	down
MOXD1	-1.393442338	0.01284963	0.01841521	down
SYTL3	-1.393442338	0.01284963	0.01841521	down
ZNF626	-1.393442338	2.25E-10	4.94E-10	down
SLC5A3	-1.393057219	2.29E-288	1.23E-286	down
PPP2R5C	-1.392116941	1.00E-101	1.16E-100	down
TRANK1	-1.391593914	1.37E-13	3.34E-13	down
NKTR	-1.391396169	2.92E-77	2.48E-76	down
DENND4C	-1.391276454	1.01E-52	5.75E-52	down
RBMXL1	-1.391217673	1.89E-41	8.80E-41	down
ZUP1	-1.390649123	1.79E-17	4.88E-17	down
MYNN	-1.390600708	1.44E-40	6.62E-40	down
ARHGEF9	-1.390075487	9.54E-15	2.41E-14	down
EPM2AIP1	-1.389272696	5.38E-44	2.64E-43	down
LIMS1	-1.388059732	2.35E-140	4.17E-139	down
RCHY1	-1.387967208	7.42E-34	2.97E-33	down
UBXN2B	-1.387237189	1.14E-40	5.24E-40	down
CUL4B	-1.387183348	8.39E-142	1.51E-140	down
C15orf41	-1.386977674	8.46E-12	1.95E-11	down
RNF2	-1.386916404	7.97E-49	4.28E-48	down
CPEB2	-1.386015882	5.67E-102	6.58E-101	down
BPGM	-1.385390066	3.37E-26	1.13E-25	down
RECK	-1.385390066	5.36E-18	1.48E-17	down
FANCD2	-1.385320077	4.18E-155	8.72E-154	down
PIBF1	-1.385294785	1.65E-20	4.87E-20	down
ZNF570	-1.384955872	2.45E-22	7.54E-22	down
KLHL24	-1.384900602	2.41E-27	8.31E-27	down
EIF5A2	-1.384836547	2.44E-37	1.05E-36	down
GPR155	-1.384672128	1.75E-06	3.26E-06	down
ZBED8	-1.384672128	4.78E-09	9.99E-09	down
CCNC	-1.384070504	3.50E-118	4.94E-117	down
TM2D1	-1.383982009	3.89E-27	1.33E-26	down
WASHC3	-1.383813637	2.86E-15	7.35E-15	down
DPY30	-1.383318121	2.82E-23	8.87E-23	down
ZFP1	-1.382995551	4.62E-12	1.07E-11	down
CFAP44	-1.382768837	1.16E-07	2.29E-07	down
PARP9	-1.382768837	6.64E-14	1.64E-13	down
SUCLA2	-1.382532064	1.08E-61	7.20E-61	down
RUFY3	-1.380963399	2.29E-17	6.23E-17	down
NMD3	-1.380459378	8.87E-104	1.05E-102	down
C1orf112	-1.380266949	3.99E-28	1.41E-27	down
SPA17	-1.37995426	1.20E-22	3.71E-22	down
ABI2	-1.379168207	1.21E-118	1.72E-117	down

ZNF215	-1.378574171	1.38E-23	4.35E-23	down
UHMK1	-1.377974119	0	0	down
DPH3	-1.377617371	6.74E-47	3.50E-46	down
KIAA1324L	-1.377043849	4.41E-32	1.70E-31	down
GALM	-1.376954215	9.44E-07	1.78E-06	down
MRPL1	-1.376528955	7.15E-21	2.12E-20	down
PRIMPOL	-1.376368825	1.39E-15	3.60E-15	down
SPATA5	-1.376116462	6.85E-18	1.89E-17	down
NUF2	-1.37574654	4.31E-87	4.08E-86	down
CCSER2	-1.375716169	1.49E-56	9.09E-56	down
ZNF426	-1.375469396	8.82E-44	4.32E-43	down
AASDHPPT	-1.375397358	1.47E-123	2.21E-122	down
EXOC1	-1.375302859	3.04E-36	1.28E-35	down
TMEM167A	-1.375137448	7.00E-136	1.18E-134	down
HOXA1	-1.374583311	7.39E-05	0.00012556	down
LIN7A	-1.374583311	0.00121192	0.00190301	down
APAF1	-1.373411658	4.12E-65	2.91E-64	down
POLR2K	-1.373411658	4.12E-65	2.91E-64	down
PCLAF	-1.373407424	2.26E-54	1.33E-53	down
C10orf88	-1.37321453	8.96E-20	2.59E-19	down
ATAD2B	-1.372452823	4.29E-19	1.22E-18	down
EIF3E	-1.372250633	0	0	down
PRELID3B	-1.37198503	2.36E-229	8.99E-228	down
CPSF2	-1.3712196	1.21E-111	1.58E-110	down
LCORL	-1.3712196	4.91E-20	1.43E-19	down
SEC24D	-1.371074525	6.18E-44	3.03E-43	down
ROCK2	-1.371026588	6.50E-85	6.00E-84	down
RRM2B	-1.370688133	8.07E-81	7.11E-80	down
HDAC8	-1.370109834	7.67E-10	1.65E-09	down
HSDL1	-1.369754202	4.27E-73	3.40E-72	down
TRAM1	-1.369742701	1.80E-222	6.49E-221	down
MRTFB	-1.369671891	2.14E-94	2.25E-93	down
DCTN6	-1.368518083	5.27E-24	1.69E-23	down
DTWD1	-1.368413544	1.38E-13	3.36E-13	down
DSCC1	-1.367928251	1.15E-73	9.20E-73	down
SEPSECS	-1.367907246	8.56E-11	1.91E-10	down
TMEM154	-1.367907246	8.56E-11	1.91E-10	down
NCR3LG1	-1.367663897	4.63E-14	1.15E-13	down
ZNF121	-1.367613756	2.32E-135	3.91E-134	down
C8orf37	-1.36758417	1.09E-15	2.84E-15	down
PSIP1	-1.367268605	1.03E-145	1.94E-144	down
ADAL	-1.366970127	2.86E-11	6.48E-11	down
SACS	-1.366753782	4.09E-200	1.26E-198	down
FRS2	-1.366730496	1.20E-37	5.20E-37	down
WRN	-1.366362804	4.74E-30	1.75E-29	down
PSD3	-1.366310307	7.12E-67	5.13E-66	down
TCEA1	-1.365538727	1.67E-139	2.94E-138	down
TTC39B	-1.365060537	1.39E-10	3.07E-10	down
PLS3	-1.364505172	3.74E-241	1.53E-239	down
RAD18	-1.363479374	4.35E-107	5.39E-106	down
TMEM128	-1.36332235	9.66E-16	2.52E-15	down
GFM2	-1.362960526	1.32E-63	9.06E-63	down
KCNIP4	-1.362854018	0.00203238	0.00313898	down
SALL2	-1.362854018	0.00203238	0.00313898	down

ARFGEF1	-1.360859417	4.92E-84	4.48E-83	down
AHR	-1.360711136	3.01E-26	1.01E-25	down
CACNA2D1	-1.360469398	2.86E-29	1.03E-28	down
NCAPG	-1.359713627	4.13E-210	1.37E-208	down
RASSF4	-1.359316554	4.50E-07	8.62E-07	down
ABCA12	-1.358508548	0.00020284	0.00033567	down
TMEM69	-1.358314115	6.94E-48	3.66E-47	down
ADGRL2	-1.357981343	3.07E-27	1.06E-26	down
TAF1B	-1.357981343	3.07E-27	1.06E-26	down
FMR1	-1.357651077	6.28E-101	7.19E-100	down
IDE	-1.35759926	9.39E-125	1.42E-123	down
SVIP	-1.357569774	3.74E-35	1.54E-34	down
SRSF11	-1.356881876	1.95E-262	9.21E-261	down
UBN2	-1.356837021	6.52E-42	3.07E-41	down
SOX9	-1.356651718	4.78E-110	6.16E-109	down
MCM9	-1.356420207	5.61E-21	1.67E-20	down
DNAH5	-1.35624372	2.11E-05	3.70E-05	down
UEVLD	-1.35624372	2.05E-25	6.74E-25	down
MTA3	-1.355120409	3.94E-53	2.26E-52	down
LIPA	-1.355091632	2.80E-128	4.38E-127	down
CERS6	-1.35506698	7.01E-143	1.28E-141	down
PRKACB	-1.35485384	1.05E-45	5.32E-45	down
STRN	-1.354486201	7.46E-106	9.07E-105	down
C5orf15	-1.353938715	2.66E-94	2.79E-93	down
OTUD4	-1.353935954	6.61E-106	8.05E-105	down
METTL5	-1.353688606	3.06E-32	1.19E-31	down
SLC30A1	-1.352816032	9.84E-77	8.27E-76	down
AKAP9	-1.352407832	6.21E-111	8.08E-110	down
TCF12	-1.351907873	1.60E-90	1.59E-89	down
FAM114A1	-1.351863234	7.30E-42	3.43E-41	down
NEMP2	-1.351168271	5.89E-15	1.50E-14	down
RPS6KB1	-1.350798001	3.13E-82	2.81E-81	down
LARP7	-1.350537837	1.41E-39	6.35E-39	down
PGM2L1	-1.350519606	4.81E-40	2.18E-39	down
RWDD1	-1.349921257	3.28E-66	2.35E-65	down
CLASP2	-1.349326061	7.39E-103	8.63E-102	down
FAXDC2	-1.349048219	0.03845577	0.0520002	down
LIFR	-1.349048219	6.64E-43	3.19E-42	down
SPDYE2	-1.349048219	0.03845577	0.0520002	down
TIMD4	-1.349048219	0.01123903	0.01621819	down
YAF2	-1.349048219	3.05E-45	1.53E-44	down
ZNF17	-1.349048219	3.47E-05	6.02E-05	down
ZNF443	-1.349048219	3.97E-07	7.63E-07	down
SPDL1	-1.348754481	2.58E-97	2.81E-96	down
PTPN13	-1.348118348	6.24E-32	2.39E-31	down
NFYB	-1.348088661	5.40E-31	2.03E-30	down
ZNF813	-1.347701793	1.51E-22	4.67E-22	down
RNF13	-1.347392803	1.45E-52	8.23E-52	down
BLM	-1.347043083	1.10E-43	5.37E-43	down
DMTF1	-1.346854005	7.94E-53	4.54E-52	down
EVI5	-1.346584179	3.22E-47	1.68E-46	down
APPL2	-1.34594898	7.28E-38	3.17E-37	down
YIPF4	-1.344798746	3.42E-41	1.59E-40	down
FGFR1OP2	-1.344080411	1.60E-63	1.10E-62	down

ESD	-1.343952152	4.16E-111	5.43E-110	down
RELCH	-1.34371022	1.21E-69	9.11E-69	down
PDLIM5	-1.343155651	8.32E-87	7.85E-86	down
KIF11	-1.343083299	8.34E-207	2.71E-205	down
PFKFB2	-1.342534912	4.86E-53	2.79E-52	down
FAM76A	-1.342475565	4.68E-10	1.01E-09	down
AMOT	-1.341816649	3.59E-13	8.67E-13	down
SMC6	-1.340985231	4.68E-74	3.77E-73	down
CBX3	-1.340765726	8.11E-226	3.00E-224	down
GK5	-1.340497915	5.95E-44	2.92E-43	down
NEK1	-1.340224368	1.45E-20	4.27E-20	down
POLR3F	-1.340115094	5.16E-39	2.30E-38	down
PGBD4	-1.340003079	0.00017825	0.00029612	down
SOS2	-1.339930874	1.56E-59	9.99E-59	down
TPR	-1.339688442	2.11E-279	1.08E-277	down
LDAH	-1.339525445	3.74E-19	1.06E-18	down
BRCA1	-1.338494441	1.64E-106	2.01E-105	down
USP33	-1.338444652	8.09E-114	1.09E-112	down
TOPBP1	-1.338431305	5.01E-163	1.13E-161	down
TRUB1	-1.337932187	5.57E-54	3.24E-53	down
MEIOC	-1.336109163	0.00178058	0.00276396	down
PTPDC1	-1.336109163	2.65E-27	9.13E-27	down
TAF1A	-1.335109028	1.73E-13	4.21E-13	down
TRIM23	-1.334664301	1.97E-30	7.32E-30	down
FBXO43	-1.334548649	3.71E-09	7.80E-09	down
PEX3	-1.334548649	1.73E-24	5.60E-24	down
HEATR5A	-1.334055504	4.34E-42	2.05E-41	down
PTBP3	-1.333632378	0	0	down
IDI1	-1.332813116	3.28E-150	6.46E-149	down
CYP24A1	-1.332560096	0.0057697	0.00855227	down
PPM1A	-1.332336492	5.42E-69	4.04E-68	down
LRIF1	-1.332281368	1.05E-55	6.29E-55	down
VTI1B	-1.332230601	2.15E-47	1.12E-46	down
RSRC1	-1.332069884	2.05E-32	7.99E-32	down
MEX3C	-1.331069175	3.53E-67	2.55E-66	down
CEP295	-1.330848545	2.34E-54	1.37E-53	down
IKZF5	-1.330792809	3.33E-27	1.14E-26	down
ZNF548	-1.330528131	6.04E-09	1.26E-08	down
FOXP1	-1.329939396	0.00029534	0.00048263	down
PLSCR4	-1.329939396	0.00029534	0.00048263	down
VAV3	-1.329419412	4.00E-35	1.65E-34	down
EID1	-1.329363026	5.17E-107	6.40E-106	down
ZNF124	-1.329148661	1.35E-12	3.20E-12	down
MAP4K3	-1.329088225	8.67E-58	5.37E-57	down
WDR35	-1.328870337	3.45E-34	1.39E-33	down
RASA2	-1.328289659	2.98E-33	1.18E-32	down
MIA3	-1.326328142	4.38E-71	3.39E-70	down
SAXO2	-1.326328142	0.01924763	0.02704636	down
ZNF816-ZNF	-1.326328142	9.84E-09	2.03E-08	down
C16orf87	-1.325740845	3.66E-19	1.04E-18	down
FAM126A	-1.325715715	1.86E-85	1.72E-84	down
NEDD4	-1.324651564	2.17E-306	1.25E-304	down
TMTC2	-1.324325789	5.15E-17	1.38E-16	down
THOC1	-1.32419303	1.07E-49	5.86E-49	down

SPIN3	-1.323577531	5.32E-09	1.11E-08	down
TRIP11	-1.3233381	8.77E-43	4.21E-42	down
KDELR3	-1.32305301	1.48E-48	7.94E-48	down
C3orf38	-1.322672803	1.77E-32	6.90E-32	down
ZNF562	-1.322081171	8.50E-74	6.82E-73	down
ZNF699	-1.322081171	1.18E-13	2.89E-13	down
CHD1	-1.321727219	2.14E-101	2.46E-100	down
SMC3	-1.321677498	3.85E-216	1.35E-214	down
PLBD1	-1.321033843	0.00298592	0.00454156	down
TPD52	-1.321033843	7.12E-98	7.84E-97	down
TRPM7	-1.32093305	4.84E-64	3.35E-63	down
SESTD1	-1.320522521	2.82E-26	9.45E-26	down
ZNF226	-1.320522521	6.43E-14	1.59E-13	down
SPAST	-1.319408728	1.38E-32	5.37E-32	down
USP45	-1.319300875	2.10E-23	6.61E-23	down
SEC24B	-1.318523352	1.48E-61	9.81E-61	down
UFM1	-1.318431896	2.99E-76	2.50E-75	down
THOC2	-1.318168906	4.46E-189	1.29E-187	down
LUZP2	-1.317715012	5.11E-11	1.15E-10	down
KIF18A	-1.317378392	3.08E-92	3.14E-91	down
PRR16	-1.316867813	4.68E-09	9.78E-09	down
PPP1R12A	-1.316226311	9.66E-116	1.33E-114	down
FAM13B	-1.316059807	4.12E-43	1.99E-42	down
E2F7	-1.314041769	2.76E-108	3.45E-107	down
RP2	-1.313631681	1.03E-74	8.40E-74	down
BBS12	-1.312522343	4.41E-05	7.59E-05	down
MSR1	-1.312522343	0.00025849	0.00042464	down
NIPAL2	-1.312522343	0.00025849	0.00042464	down
RCAN1	-1.312522343	5.16E-52	2.91E-51	down
SHISA3	-1.312522343	0.00977882	0.01419297	down
NMI	-1.311648247	3.59E-20	1.05E-19	down
ITFG1	-1.311433927	4.63E-61	3.04E-60	down
ORC4	-1.311116892	3.86E-59	2.46E-58	down
CENPC	-1.310800749	1.96E-20	5.76E-20	down
HEATR5B	-1.310759734	1.71E-47	8.94E-47	down
CNOT6	-1.309914184	1.34E-106	1.65E-105	down
MAPK9	-1.309268317	1.83E-105	2.22E-104	down
FGD6	-1.309222238	7.76E-75	6.35E-74	down
PKN2	-1.30892139	9.35E-158	2.00E-156	down
EAF2	-1.308406234	2.35E-05	4.11E-05	down
JMJD1C	-1.308406234	6.43E-111	8.37E-110	down
VPS13B	-1.308357967	1.83E-63	1.26E-62	down
NOL8	-1.30831255	4.61E-97	5.01E-96	down
PIGM	-1.307689189	2.74E-18	7.63E-18	down
MUT	-1.307379039	2.67E-42	1.27E-41	down
WDR36	-1.30720994	2.32E-128	3.63E-127	down
HCAR1	-1.307164138	1.14E-25	3.78E-25	down
REST	-1.306989216	1.38E-62	9.35E-62	down
ZNF169	-1.306819983	3.88E-07	7.45E-07	down
C5orf24	-1.306743185	2.73E-162	6.12E-161	down
INTS8	-1.306649635	2.83E-86	2.65E-85	down
CCAR1	-1.306596077	3.24E-185	9.09E-184	down
PIK3C3	-1.306187004	3.01E-47	1.57E-46	down
MAMDC2	-1.305979497	0.00081411	0.00129316	down

CENPI	-1.305929665	2.37E-96	2.56E-95	down
GPALPP1	-1.305780765	1.84E-25	6.05E-25	down
FAM98B	-1.305569582	2.90E-59	1.84E-58	down
TTC8	-1.30545895	1.18E-10	2.61E-10	down
RPS6KA5	-1.305266527	7.07E-15	1.80E-14	down
ZDHHC20	-1.305215155	3.37E-137	5.79E-136	down
RALGPS2	-1.304536688	9.70E-64	6.70E-63	down
ANKRD18A	-1.304379169	2.11E-13	5.12E-13	down
RRAGB	-1.304122348	3.61E-09	7.59E-09	down
LPCAT2	-1.303719334	6.40E-58	3.97E-57	down
CRYGS	-1.303244529	0.00502195	0.00747868	down
CRBN	-1.302161831	6.64E-51	3.70E-50	down
KIZ	-1.302161831	6.23E-14	1.54E-13	down
RAB39B	-1.302161831	1.12E-07	2.21E-07	down
XPO4	-1.302004471	1.39E-119	1.99E-118	down
CFL2	-1.301914243	1.19E-91	1.20E-90	down
KIF2A	-1.301616389	1.06E-103	1.25E-102	down
FZD3	-1.301519849	3.40E-13	8.22E-13	down
ZIC5	-1.30079305	6.32E-07	1.20E-06	down
TAX1BP1	-1.300677129	3.37E-110	4.35E-109	down
MATR3	-1.300663191	0	0	down
SUGT1	-1.300408963	3.06E-67	2.22E-66	down
IL1RAP	-1.300332934	4.47E-29	1.61E-28	down
WASHC5	-1.299852681	5.21E-113	6.92E-112	down
C17orf80	-1.299818512	1.90E-40	8.69E-40	down
NUDCD1	-1.299579542	1.22E-77	1.04E-76	down
SNCA	-1.299007536	3.59E-06	6.56E-06	down
TRAPPC8	-1.298855305	1.17E-96	1.26E-95	down
MAP3K20	-1.298830909	5.46E-138	9.46E-137	down
RBBP8	-1.298478359	2.07E-115	2.84E-114	down
ZNF585A	-1.297649066	3.01E-12	7.05E-12	down
RNF19A	-1.297537493	3.33E-49	1.80E-48	down
HDAC2	-1.297520877	2.10E-158	4.56E-157	down
RC3H2	-1.296855258	1.15E-166	2.75E-165	down
ANKRD61	-1.296580799	0.03322021	0.04537047	down
ATP6V1C2	-1.296580799	0.03322021	0.04537047	down
BCL2	-1.296580799	1.62E-14	4.07E-14	down
FSIP1	-1.296580799	0.00260008	0.00397223	down
GNAL	-1.296580799	0.03322021	0.04537047	down
HPGD	-1.296580799	0.03322021	0.04537047	down
SDSL	-1.296580799	0.03322021	0.04537047	down
ME1	-1.296066927	8.01E-90	7.89E-89	down
DOCK7	-1.296004896	5.92E-100	6.70E-99	down
GPRASP2	-1.293553106	5.14E-09	1.07E-08	down
TMEM30A	-1.293176254	9.69E-286	5.11E-284	down
RAB23	-1.292567736	2.38E-21	7.15E-21	down
CCDC90B	-1.292337575	1.38E-33	5.50E-33	down
HSP90B1	-1.292315864	0	0	down
FBXL5	-1.292286569	6.94E-70	5.23E-69	down
NIPBL	-1.292194371	5.23E-181	1.42E-179	down
MTR	-1.291649178	9.37E-59	5.91E-58	down
ZNF277	-1.291562724	2.32E-35	9.60E-35	down
CHUK	-1.291324413	9.42E-64	6.51E-63	down
EFR3A	-1.291178807	2.01E-155	4.22E-154	down

TMPO	-1.291112506	0	0	down
AIDA	-1.291026352	6.18E-121	8.98E-120	down
TARBP1	-1.290402224	3.65E-27	1.25E-26	down
CDC27	-1.290221618	7.19E-236	2.86E-234	down
FAM171B	-1.289947706	3.50E-32	1.35E-31	down
UBXN4	-1.289886146	2.64E-177	6.84E-176	down
ABRACL	-1.289056556	5.72E-39	2.55E-38	down
WDR41	-1.288919443	2.00E-70	1.52E-69	down
SLC30A7	-1.288853437	1.05E-59	6.71E-59	down
KLHDC2	-1.288605003	6.10E-20	1.77E-19	down
KMT2E	-1.288265531	5.42E-30	1.99E-29	down
CCDC146	-1.287647674	0.01665749	0.02357301	down
PPIL4	-1.287647674	2.81E-34	1.14E-33	down
ZNF429	-1.287647674	3.37E-05	5.84E-05	down
ZNF607	-1.287647674	2.38E-10	5.22E-10	down
LIN9	-1.287381862	1.54E-34	6.27E-34	down
DYNC1I2	-1.286934704	1.42E-111	1.87E-110	down
ZNF614	-1.286268424	1.32E-39	5.96E-39	down
ZNF644	-1.286201653	6.30E-92	6.39E-91	down
ZNF343	-1.285996048	9.02E-07	1.70E-06	down
TOPORS	-1.285659512	5.96E-63	4.05E-62	down
TRIB2	-1.285609681	4.86E-23	1.52E-22	down
KDELC2	-1.284893079	4.40E-93	4.52E-92	down
ZCCHC9	-1.284385502	1.16E-33	4.63E-33	down
SUMO1	-1.282644912	2.85E-131	4.61E-130	down
NR5A2	-1.281934023	1.13E-10	2.51E-10	down
ZCRB1	-1.28183878	4.28E-76	3.57E-75	down
VTA1	-1.281727496	2.56E-70	1.95E-69	down
LIN54	-1.28170191	2.94E-32	1.14E-31	down
BORA	-1.281603698	3.74E-23	1.17E-22	down
INTS6	-1.281595746	1.54E-64	1.08E-63	down
SCLT1	-1.281495447	4.91E-34	1.97E-33	down
CREBRF	-1.281361411	5.23E-14	1.30E-13	down
ATP1B1	-1.281205573	1.98E-79	1.71E-78	down
ACSL3	-1.279986127	4.38E-179	1.16E-177	down
ARHGAP42	-1.279785556	1.80E-11	4.11E-11	down
HPRT1	-1.279555919	2.07E-114	2.81E-113	down
STRN3	-1.278560611	6.79E-52	3.83E-51	down
KIAA0753	-1.278456251	4.83E-26	1.61E-25	down
HEXA	-1.277965121	5.53E-05	9.47E-05	down
LIMA1	-1.277965121	4.59E-14	1.14E-13	down
SATB2	-1.277633885	9.30E-38	4.04E-37	down
RCBTB1	-1.276898433	2.00E-46	1.03E-45	down
MBNL2	-1.276847804	1.47E-113	1.97E-112	down
KIF3A	-1.275815524	8.85E-49	4.75E-48	down
RBM27	-1.275519183	1.71E-45	8.60E-45	down
PPP4R2	-1.275127061	2.54E-131	4.10E-130	down
RTN4IP1	-1.27473641	3.12E-18	8.67E-18	down
EEF1AKMT3	-1.274435158	5.46E-10	1.18E-09	down
SACM1L	-1.274404008	1.13E-81	1.00E-80	down
ECT2	-1.273729473	0	0	down
HIPK3	-1.273633923	1.01E-139	1.79E-138	down
ZC3H13	-1.273107946	1.65E-78	1.42E-77	down
ZNF417	-1.27205848	1.19E-13	2.92E-13	down

TNFAIP8	-1.272028517	1.29E-30	4.81E-30	down
MBD5	-1.271880358	2.55E-11	5.78E-11	down
UBTD2	-1.271807297	2.81E-60	1.82E-59	down
CLK4	-1.271663167	2.16E-21	6.50E-21	down
CAMK4	-1.271535669	1.56E-26	5.27E-26	down
BMPR1B	-1.271045707	1.28E-06	2.41E-06	down
CNTRL	-1.270689266	8.45E-36	3.53E-35	down
MMD	-1.270396867	2.14E-20	6.27E-20	down
IL17RB	-1.269613751	0.00032476	0.00052982	down
SHLD3	-1.269613751	0.00032476	0.00052982	down
SLC25A16	-1.269253168	5.64E-22	1.72E-21	down
HELQ	-1.269194911	2.59E-10	5.66E-10	down
NPAT	-1.268674802	3.54E-56	2.14E-55	down
TRIM33	-1.268429758	6.20E-128	9.63E-127	down
SRFBP1	-1.267911456	5.57E-21	1.66E-20	down
C5orf22	-1.267514661	4.33E-78	3.70E-77	down
KIFAP3	-1.267434453	7.77E-32	2.97E-31	down
POU2F1	-1.267434453	6.62E-52	3.73E-51	down
PTPN12	-1.267434453	2.76E-155	5.78E-154	down
FAM91A1	-1.267293709	8.87E-154	1.80E-152	down
POLG2	-1.266586059	1.93E-12	4.56E-12	down
TTC23	-1.265854283	3.09E-13	7.48E-13	down
SUDS3	-1.265854283	9.57E-60	6.14E-59	down
LYPLA1	-1.265673458	3.40E-147	6.55E-146	down
ZNF594	-1.265216628	7.72E-10	1.66E-09	down
PGM2	-1.26480209	2.94E-49	1.59E-48	down
SLC17A5	-1.263887567	9.06E-26	3.00E-25	down
GOLGA4	-1.263668087	4.30E-130	6.85E-129	down
FP565260.6	-1.263318345	1.12E-06	2.10E-06	down
LCOR	-1.262959242	1.53E-61	1.01E-60	down
DNAJB4	-1.26248166	2.71E-13	6.56E-13	down
WDR44	-1.262336586	1.80E-43	8.74E-43	down
ARID2	-1.261420345	5.69E-92	5.78E-91	down
CAND1	-1.261017454	2.70E-263	1.28E-261	down
BTBD1	-1.260810731	1.71E-126	2.63E-125	down
TOP2A	-1.260637919	0	0	down
GTF2A1	-1.260576881	1.70E-75	1.40E-74	down
TUBD1	-1.260371061	3.65E-10	7.94E-10	down
OSBPL9	-1.260168905	1.52E-82	1.37E-81	down
ZNF800	-1.259681216	1.30E-62	8.81E-62	down
SYNE2	-1.259586591	6.80E-251	2.99E-249	down
ZMYND11	-1.259016018	1.14E-75	9.46E-75	down
NOC3L	-1.259011648	1.88E-63	1.29E-62	down
GPBP1	-1.258577489	1.61E-116	2.24E-115	down
PNISR	-1.258402854	3.00E-63	2.05E-62	down
NBN	-1.258168883	1.73E-120	2.51E-119	down
UBE4A	-1.257675955	2.79E-132	4.56E-131	down
CLDND1	-1.257203782	1.55E-61	1.03E-60	down
PPWD1	-1.257187361	4.93E-32	1.90E-31	down
DCUN1D1	-1.256575625	4.53E-61	2.98E-60	down
DZIP1	-1.256458611	2.17E-74	1.76E-73	down
AGPAT4	-1.255938814	3.32E-14	8.29E-14	down
C2orf88	-1.255938814	0.00028227	0.000462	down
CDKL2	-1.255938814	0.00028227	0.000462	down

ENOX1	-1.255938814	0.01436302	0.02045992	down
HOXA5	-1.255938814	0.00014913	0.00024895	down
KDM7A	-1.255938814	3.32E-14	8.29E-14	down
MBOAT2	-1.255938814	1.55E-47	8.12E-47	down
METTL14	-1.255938814	7.57E-17	2.03E-16	down
RAB29	-1.255938814	6.32E-34	2.53E-33	down
RBM11	-1.255938814	0.00028227	0.000462	down
SHROOM4	-1.255938814	0.02855284	0.03935428	down
SLC25A12	-1.255938814	1.61E-30	6.02E-30	down
STOX2	-1.255938814	0.00732471	0.01074297	down
TIGAR	-1.255938814	0.00377258	0.00568349	down
TRAF3IP2	-1.255938814	4.20E-05	7.23E-05	down
UTP23	-1.255938814	3.09E-35	1.28E-34	down
ZCWPW1	-1.255938814	0.00377258	0.00568349	down
ZNF221	-1.255938814	0.00732471	0.01074297	down
SUCO	-1.255315485	3.13E-62	2.11E-61	down
MICU2	-1.2548892	1.19E-37	5.16E-37	down
LEPROT	-1.254877619	1.52E-143	2.79E-142	down
ENAH	-1.254805363	9.46E-201	2.95E-199	down
SGO1	-1.25307916	3.61E-41	1.67E-40	down
OSGIN2	-1.252258468	1.55E-42	7.40E-42	down
CDK19	-1.252086489	5.92E-31	2.22E-30	down
ZNF234	-1.25186915	8.18E-11	1.82E-10	down
MOB3B	-1.251625821	2.79E-10	6.09E-10	down
ABCD3	-1.251172435	3.93E-88	3.78E-87	down
APPBP2	-1.250458485	2.31E-56	1.40E-55	down
CIR1	-1.250379289	4.09E-15	1.05E-14	down
SMAD5	-1.250256109	6.06E-107	7.48E-106	down
TCEANC2	-1.250038213	2.54E-14	6.36E-14	down
TSEN15	-1.249932939	2.52E-45	1.26E-44	down
CAAP1	-1.249553179	1.04E-42	4.98E-42	down
PFKM	-1.248997205	3.79E-50	2.09E-49	down
GLMN	-1.248575426	3.27E-37	1.40E-36	down
FAM111A	-1.248521343	2.35E-157	5.01E-156	down
ATAD2	-1.248140235	0	0	down
HELLS	-1.247804387	7.45E-66	5.30E-65	down
YPEL5	-1.247804387	8.30E-34	3.32E-33	down
UBA3	-1.24775909	1.38E-74	1.12E-73	down
EIF2A	-1.247060663	2.02E-106	2.48E-105	down
HIF1A	-1.247001185	0	0	down
NBEA	-1.245954726	5.26E-09	1.10E-08	down
SP100	-1.245827498	3.72E-42	1.76E-41	down
NR1D2	-1.245425588	1.38E-40	6.34E-40	down
INSYN2B	-1.245212408	6.87E-05	0.00011698	down
MINDY1	-1.245212408	5.39E-12	1.25E-11	down
DNA2	-1.244689673	1.78E-54	1.05E-53	down
CYB5R4	-1.244653007	5.10E-51	2.84E-50	down
WDR12	-1.244216329	3.70E-90	3.65E-89	down
MYO9A	-1.244179101	2.13E-36	9.03E-36	down
BTAF1	-1.243926297	9.20E-79	7.93E-78	down
ASPH	-1.243839337	7.23E-118	1.02E-116	down
UBR2	-1.243442718	6.56E-64	4.54E-63	down
LTA4H	-1.241254769	4.85E-82	4.35E-81	down
ARHGEF40	-1.241192229	4.59E-09	9.59E-09	down

RAB38	-1.240672058	0.0008855	0.00140289	down
UGT8	-1.239336847	4.34E-57	2.66E-56	down
IFT57	-1.239224941	6.55E-26	2.18E-25	down
PPP2R5E	-1.239123271	1.41E-119	2.01E-118	down
DIAPH3	-1.239055592	4.65E-93	4.77E-92	down
DENND5B	-1.239008418	4.05E-32	1.56E-31	down
NPR2	-1.238865301	9.02E-06	1.62E-05	down
SIX4	-1.238865301	1.48E-14	3.72E-14	down
USP53	-1.238865301	3.64E-36	1.53E-35	down
ATXN3	-1.238451388	4.10E-12	9.56E-12	down
UHRF1BP1L	-1.237965381	8.53E-55	5.03E-54	down
PIKFYVE	-1.237883542	7.52E-77	6.33E-76	down
LYPLAL1	-1.237560285	1.39E-11	3.19E-11	down
RAD21	-1.236761309	0	0	down
TCTN1	-1.236573489	4.00E-09	8.38E-09	down
ARFIP1	-1.23644891	3.61E-41	1.68E-40	down
SPRTN	-1.236398628	8.11E-30	2.97E-29	down
PGBD1	-1.236243013	1.59E-16	4.23E-16	down
USP25	-1.235844487	3.99E-136	6.77E-135	down
BOD1L1	-1.235823453	3.92E-67	2.84E-66	down
DFFB	-1.235666419	6.46E-07	1.23E-06	down
11-9月	-1.235381398	2.82E-223	1.02E-221	down
KATNAL1	-1.235373244	1.36E-42	6.48E-42	down
BCAT1	-1.234609257	1.48E-148	2.88E-147	down
PCF11	-1.234519831	2.01E-49	1.09E-48	down
CCP110	-1.234494563	5.91E-63	4.02E-62	down
DENR	-1.234004438	7.34E-171	1.82E-169	down
CUL2	-1.233462098	2.69E-105	3.25E-104	down
IDS	-1.233187581	2.75E-75	2.26E-74	down
SLF2	-1.233092321	1.16E-60	7.55E-60	down
CKAP2	-1.232416551	5.13E-216	1.79E-214	down
IL15	-1.232351994	4.19E-06	7.64E-06	down
VPS36	-1.232248708	4.28E-40	1.95E-39	down
DYRK2	-1.232092072	1.44E-24	4.64E-24	down
DOCK11	-1.232026194	6.36E-20	1.85E-19	down
ESR2	-1.231691268	0.00021248	0.00035086	down
RNF144B	-1.231691268	0.00021248	0.00035086	down
ITPRID2	-1.23167469	2.23E-185	6.30E-184	down
FUNDC1	-1.231533502	4.92E-21	1.47E-20	down
TGDS	-1.231462722	5.34E-15	1.36E-14	down
TIFA	-1.231462722	2.02E-28	7.17E-28	down
ANKIB1	-1.23139663	3.70E-74	2.99E-73	down
DEPDC1B	-1.23139663	8.32E-95	8.82E-94	down
FBXO38	-1.23127676	8.81E-24	2.80E-23	down
DLGAP5	-1.231269612	6.37E-175	1.62E-173	down
ITGA6	-1.230929783	2.29E-201	7.19E-200	down
MAP3K7	-1.230907587	1.40E-75	1.16E-74	down
NFIB	-1.230378588	9.10E-53	5.19E-52	down
VBP1	-1.230306502	1.02E-159	2.23E-158	down
ELMOD1	-1.229466603	1.47E-05	2.60E-05	down
MEX3A	-1.229466603	3.60E-11	8.12E-11	down
SFR1	-1.229466603	2.21E-08	4.49E-08	down
MAP3K1	-1.229017515	4.67E-46	2.39E-45	down
TMX1	-1.228717346	1.05E-160	2.31E-159	down

ATP5S	-1.228631468	1.56E-25	5.14E-25	down
SUV39H2	-1.228123436	2.22E-26	7.45E-26	down
SH3D19	-1.227907944	3.16E-44	1.56E-43	down
RFC3	-1.227892401	5.86E-111	7.64E-110	down
PGM3	-1.227777206	2.49E-68	1.83E-67	down
SLC26A2	-1.227702782	9.02E-62	6.02E-61	down
CCPG1	-1.226138037	1.68E-29	6.10E-29	down
ZNF627	-1.225242017	3.64E-06	6.66E-06	down
LIN7C	-1.225019753	1.26E-87	1.20E-86	down
NADK2	-1.224967427	1.47E-29	5.34E-29	down
RAB2B	-1.224600848	1.63E-31	6.22E-31	down
GOLGA5	-1.224481383	8.07E-27	2.74E-26	down
SUZ12	-1.22396921	1.14E-179	3.04E-178	down
RPS6KC1	-1.223831068	2.10E-27	7.28E-27	down
RAP2A	-1.223204286	3.75E-53	2.15E-52	down
TASP1	-1.22277195	2.07E-12	4.87E-12	down
ARNTL2	-1.222015672	3.05E-71	2.36E-70	down
LRRC58	-1.221927455	7.41E-133	1.22E-131	down
MTREX	-1.22126479	2.71E-133	4.49E-132	down
NFE2L3	-1.220724122	6.66E-19	1.88E-18	down
ZFHX3	-1.220234753	1.17E-45	5.91E-45	down
DHX32	-1.219885102	2.22E-30	8.23E-30	down
SAMD5	-1.217803685	4.25E-07	8.15E-07	down
MKLN1	-1.217647659	0	0	down
ACAT2	-1.217531957	4.31E-128	6.72E-127	down
FAM8A1	-1.217464666	1.27E-38	5.61E-38	down
KTN1	-1.217404213	0	0	down
CWC27	-1.2170295	5.64E-30	2.07E-29	down
KIF21A	-1.217012131	9.86E-73	7.80E-72	down
ONECUT2	-1.21641045	5.91E-06	1.07E-05	down
VRK2	-1.21641045	4.29E-33	1.69E-32	down
FICD	-1.216046087	7.90E-07	1.50E-06	down
FAM69A	-1.215781688	1.07E-07	2.11E-07	down
CSE1L	-1.215702997	0	0	down
VSIG10	-1.215497888	1.83E-31	6.97E-31	down
BAG4	-1.215030871	2.00E-35	8.30E-35	down
RNASEH2B	-1.214819827	6.13E-31	2.30E-30	down
SNX6	-1.214811809	3.55E-149	6.92E-148	down
SP4	-1.214118639	2.97E-20	8.68E-20	down
RPAP2	-1.213805452	2.23E-34	9.05E-34	down
BACH1	-1.213779141	7.63E-32	2.92E-31	down
MMACHC	-1.212986669	1.30E-10	2.88E-10	down
ABCE1	-1.212759262	1.68E-181	4.59E-180	down
SLC39A6	-1.212487451	3.86E-114	5.21E-113	down
BTG3	-1.212397388	1.56E-90	1.56E-89	down
RC3H1	-1.212241962	3.31E-45	1.66E-44	down
ABCB7	-1.212143696	7.11E-38	3.10E-37	down
GSTCD	-1.212055681	2.22E-31	8.43E-31	down
NDC1	-1.211544695	5.54E-207	1.81E-205	down
ZNF587B	-1.211400418	1.19E-16	3.17E-16	down
MMGT1	-1.211317288	4.17E-125	6.33E-124	down
MED13	-1.210984316	6.63E-125	1.01E-123	down
HSF2	-1.210957432	1.79E-27	6.21E-27	down
FAM3C	-1.210755505	3.45E-59	2.19E-58	down

C1orf131	-1.210341948	2.19E-12	5.15E-12	down
RNF141	-1.210135125	1.14E-24	3.68E-24	down
METAP2	-1.21008442	4.64E-155	9.64E-154	down
CCSAP	-1.209261075	5.64E-31	2.12E-30	down
WASF1	-1.208790333	1.67E-45	8.42E-45	down
CLGN	-1.208004337	6.60E-20	1.91E-19	down
ATAD1	-1.207753017	5.79E-59	3.67E-58	down
TMEFF2	-1.207029214	0.01060277	0.01533911	down
ZNF66	-1.207029214	0.01060277	0.01533911	down
ANKRD12	-1.20689725	1.59E-46	8.20E-46	down
MZT1	-1.206829408	2.22E-90	2.20E-89	down
C8orf59	-1.206588898	1.47E-28	5.25E-28	down
ARHGAP29	-1.205792865	0	0	down
BMPR1A	-1.205450364	1.19E-72	9.39E-72	down
IGF2BP3	-1.205408598	3.46E-27	1.19E-26	down
COPS2	-1.205245606	2.15E-74	1.74E-73	down
PRRC1	-1.204925648	1.98E-102	2.30E-101	down
NCOA7	-1.204231666	2.76E-33	1.09E-32	down
SCML2	-1.203471394	7.28E-15	1.85E-14	down
VEZT	-1.203457461	3.89E-92	3.96E-91	down
SERINC1	-1.203415442	3.86E-159	8.40E-158	down
CCNJ	-1.202827478	2.94E-21	8.81E-21	down
RHOT1	-1.202499555	5.23E-38	2.29E-37	down
NKAP	-1.202365457	1.29E-25	4.27E-25	down
DLG1	-1.20165174	9.78E-164	2.24E-162	down
CEP350	-1.201645693	3.52E-69	2.63E-68	down
CYP2J2	-1.20149103	0.00013782	0.00023062	down
PIGA	-1.20149103	2.46E-14	6.16E-14	down
DNAJB7	-1.200290785	8.31E-06	1.49E-05	down
FIGNL1	-1.20021527	3.58E-77	3.04E-76	down
TOMM40L	-1.20008558	7.44E-11	1.66E-10	down
GTF2F2	-1.199810802	2.42E-37	1.04E-36	down
SERAC1	-1.199795736	5.18E-17	1.39E-16	down
CRNKL1	-1.199714312	2.01E-54	1.18E-53	down
ZNF639	-1.199703581	6.43E-60	4.13E-59	down
BCAS2	-1.199380214	6.58E-56	3.96E-55	down
ZNF30	-1.198982929	8.31E-09	1.72E-08	down
TRAPPC11	-1.198944658	2.60E-31	9.85E-31	down
DST	-1.198851236	1.09E-164	2.51E-163	down
UCHL5	-1.198510534	9.34E-69	6.94E-68	down
PRMT6	-1.198426356	5.04E-22	1.54E-21	down
NUS1	-1.19738185	7.01E-85	6.47E-84	down
PDS5B	-1.197248193	1.12E-70	8.56E-70	down
MME	-1.197045125	0.02091206	0.02925784	down
SP3	-1.197045125	1.01E-144	1.89E-143	down
ZNF442	-1.197045125	0.02091206	0.02925784	down
ZNF808	-1.197045125	3.85E-06	7.04E-06	down
ZNF480	-1.195796579	2.60E-35	1.08E-34	down
MAGED2	-1.194597166	9.57E-76	7.95E-75	down
UGGT2	-1.194589474	4.01E-30	1.48E-29	down
DLD	-1.193109904	4.52E-97	4.91E-96	down
DGKE	-1.192646661	5.29E-27	1.81E-26	down
PAPOLG	-1.19227062	6.57E-28	2.30E-27	down
FAM45A	-1.191506942	1.25E-11	2.87E-11	down

CD164	-1.191240147	2.24E-261	1.04E-259	down
ASCC3	-1.190666213	2.36E-99	2.66E-98	down
CAPZA2	-1.190460906	6.37E-176	1.64E-174	down
GTF3C3	-1.190424209	5.09E-54	2.96E-53	down
CYB5RL	-1.190350473	5.90E-12	1.37E-11	down
TRMT10B	-1.190350473	7.49E-10	1.61E-09	down
RAB6A	-1.189897009	2.44E-154	5.01E-153	down
ING3	-1.189849624	4.73E-17	1.27E-16	down
NF1	-1.189701713	3.83E-197	1.16E-195	down
RPS6KA3	-1.189428446	1.74E-43	8.47E-43	down
CIAO2A	-1.189267775	1.01E-33	4.02E-33	down
SHLD2	-1.188984376	1.64E-80	1.43E-79	down
FAM204A	-1.187951977	1.52E-30	5.65E-30	down
C8orf76	-1.187767312	0.00908852	0.0132393	down
ZNF845	-1.187767312	5.33E-24	1.70E-23	down
ZNF561	-1.187621871	2.64E-38	1.16E-37	down
USP9X	-1.184831224	0	0	down
SLU7	-1.183753629	3.98E-49	2.15E-48	down
PPP1R3C	-1.183224631	1.88E-28	6.66E-28	down
KLHL5	-1.183082472	8.46E-47	4.38E-46	down
ATP11B	-1.183038267	8.58E-80	7.46E-79	down
NSA2	-1.182817478	2.45E-60	1.59E-59	down
KIAA0895	-1.182587073	6.97E-15	1.77E-14	down
HOXD8	-1.181938233	0.00401489	0.00603855	down
PROX2	-1.181938233	0.00401489	0.00603855	down
ZFP69B	-1.181938233	1.25E-10	2.76E-10	down
FYCO1	-1.181358488	2.73E-31	1.03E-30	down
XIAP	-1.180781455	1.90E-90	1.88E-89	down
TMEM107	-1.180448615	2.92E-07	5.64E-07	down
GDAP2	-1.180400342	1.53E-29	5.58E-29	down
CDK1	-1.180114729	3.40E-198	1.04E-196	down
ZNF430	-1.179989961	2.30E-10	5.05E-10	down
CIPC	-1.179267249	6.69E-37	2.85E-36	down
CD47	-1.178862591	5.59E-60	3.60E-59	down
CUZD1	-1.177936302	0.00179416	0.00278402	down
ZSCAN30	-1.177124431	2.00E-10	4.39E-10	down
SREK1	-1.17672165	8.97E-111	1.16E-109	down
CDCA7	-1.176720524	1.25E-63	8.62E-63	down
NDUFS4	-1.176643933	3.08E-32	1.19E-31	down
METTL8	-1.176448221	2.23E-23	7.05E-23	down
MSI2	-1.176394616	9.93E-74	7.96E-73	down
WDFY1	-1.175928176	1.48E-76	1.24E-75	down
IMPA1	-1.175507189	4.93E-65	3.46E-64	down
AP3B1	-1.175207542	6.92E-100	7.81E-99	down
C19orf44	-1.175018819	4.08E-05	7.04E-05	down
NRP2	-1.175018819	0.01785236	0.02520101	down
STON1	-1.175018819	0.01785236	0.02520101	down
TFAM	-1.174828256	1.74E-163	3.97E-162	down
KDELC1	-1.174474714	5.64E-13	1.35E-12	down
HNRNPH2	-1.174111015	2.59E-49	1.40E-48	down
FAM173B	-1.174096792	3.39E-35	1.40E-34	down
LEPROTL1	-1.174030559	3.40E-39	1.52E-38	down
ANGEL2	-1.173882093	2.72E-34	1.10E-33	down
SLC4A7	-1.173702941	4.65E-226	1.73E-224	down

MTIF2	-1.173476654	1.07E-49	5.86E-49	down
EML4	-1.173270097	1.90E-156	4.03E-155	down
GNPTAB	-1.172913463	1.56E-48	8.36E-48	down
1-3月	-1.172797579	0.00036695	0.0005964	down
FBXO3	-1.172744879	3.63E-53	2.08E-52	down
LBR	-1.172643782	1.48E-177	3.85E-176	down
RAI14	-1.172170457	6.89E-243	2.84E-241	down
ZEB1	-1.170617	5.26E-24	1.68E-23	down
GSKIP	-1.170584756	1.88E-22	5.80E-22	down
TM9SF3	-1.169545503	3.63E-257	1.65E-255	down
NAPB	-1.169227181	3.50E-12	8.17E-12	down
PRKAR1A	-1.168102169	3.26E-234	1.28E-232	down
FAM172A	-1.168066058	5.09E-21	1.52E-20	down
HSD17B7	-1.167750818	1.53E-23	4.82E-23	down
BAG2	-1.167683066	1.20E-51	6.72E-51	down
CCDC180	-1.16750085	1.63E-05	2.87E-05	down
VSIG1	-1.16750085	1.63E-05	2.87E-05	down
STX7	-1.167291457	3.80E-45	1.90E-44	down
HDHD2	-1.16703831	3.16E-29	1.14E-28	down
SRSF10	-1.166978839	1.43E-154	2.95E-153	down
PAPOLA	-1.166428859	1.82E-236	7.29E-235	down
RIT1	-1.165794191	8.08E-21	2.40E-20	down
EIF1AX	-1.165453955	4.70E-76	3.91E-75	down
LCLAT1	-1.165434125	1.53E-31	5.81E-31	down
RPAP3	-1.164835047	1.15E-94	1.21E-93	down
DNAJC13	-1.164683699	6.09E-91	6.09E-90	down
MRPL39	-1.164678661	1.81E-50	9.99E-50	down
KBTBD8	-1.164623648	9.40E-10	2.01E-09	down
UBE2E1	-1.163936813	7.81E-85	7.20E-84	down
ZNF765	-1.163797801	8.54E-17	2.29E-16	down
FAM133B	-1.163372696	6.09E-21	1.81E-20	down
GNA13	-1.163232905	6.88E-126	1.05E-124	down
PNPT1	-1.162983289	3.11E-110	4.03E-109	down
MEGF9	-1.16282941	5.70E-40	2.58E-39	down
PDCL	-1.162434483	9.57E-39	4.25E-38	down
BBS9	-1.162408124	1.56E-16	4.16E-16	down
CTBS	-1.162279707	3.69E-09	7.75E-09	down
VPS45	-1.161746462	9.39E-27	3.19E-26	down
DERA	-1.160896122	1.71E-26	5.78E-26	down
MPP6	-1.160876617	1.38E-31	5.24E-31	down
KATNBL1	-1.160286575	1.28E-24	4.16E-24	down
DECR1	-1.160243162	1.11E-30	4.13E-30	down
SFT2D2	-1.160078799	1.91E-110	2.47E-109	down
ZNF331	-1.159892127	8.38E-21	2.48E-20	down
VMA21	-1.159881087	7.74E-104	9.17E-103	down
SMC5	-1.159738152	5.74E-75	4.71E-74	down
MGA	-1.159365555	1.12E-61	7.48E-61	down
PRKAB2	-1.159185581	1.20E-25	3.96E-25	down
OSGEPL1	-1.158977084	3.32E-10	7.23E-10	down
AMIGO2	-1.158906734	3.50E-37	1.50E-36	down
VIRMA	-1.15875217	1.24E-86	1.16E-85	down
RSBN1L	-1.158328018	2.80E-36	1.18E-35	down
RSF1	-1.158292722	4.58E-72	3.59E-71	down
NAMPT	-1.158108253	1.13E-66	8.13E-66	down

JKAMP	-1.158021873	1.83E-56	1.11E-55	down
SSR1	-1.157388925	3.02E-203	9.60E-202	down
DENND4A	-1.157348241	3.99E-25	1.30E-24	down
TCAIM	-1.157265223	2.20E-27	7.62E-27	down
AC073111.5	-1.156403141	0.01522617	0.0216363	down
C2orf69	-1.156403141	4.17E-37	1.79E-36	down
DDHD1	-1.156403141	7.46E-35	3.06E-34	down
DNAH11	-1.156403141	0.03557046	0.04841441	down
LRRC69	-1.156403141	0.03557046	0.04841441	down
PRPF39	-1.156403141	8.75E-37	3.72E-36	down
SCART1	-1.156403141	0.03557046	0.04841441	down
ZSCAN20	-1.156403141	0.00027218	0.00044641	down
PDS5A	-1.156173996	5.44E-194	1.62E-192	down
GXYLT1	-1.154609859	4.11E-63	2.81E-62	down
RAB27A	-1.154609859	6.42E-14	1.58E-13	down
GRPEL2	-1.154340674	9.46E-34	3.78E-33	down
GOLIM4	-1.154136534	5.78E-60	3.72E-59	down
RNF146	-1.153323748	6.26E-16	1.64E-15	down
GPN3	-1.153148695	1.75E-35	7.28E-35	down
ZFP69	-1.152651006	2.27E-07	4.42E-07	down
POLR3GL	-1.152434239	4.87E-07	9.31E-07	down
ZNF654	-1.152434239	8.07E-24	2.57E-23	down
ZNF22	-1.152275255	1.30E-17	3.56E-17	down
COPS4	-1.151781583	2.07E-44	1.02E-43	down
TGFBR3	-1.151317852	3.10E-23	9.73E-23	down
LAMTOR3	-1.151241436	6.52E-23	2.03E-22	down
CCDC112	-1.151039956	9.74E-10	2.09E-09	down
TVP23B	-1.150089367	6.10E-26	2.03E-25	down
FIGN	-1.149701171	4.15E-57	2.55E-56	down
NUP35	-1.149233645	3.30E-35	1.36E-34	down
ALG6	-1.1492166	5.29E-26	1.76E-25	down
ZNF8	-1.148454388	4.01E-18	1.11E-17	down
RFC1	-1.148392598	2.99E-83	2.71E-82	down
CDC14A	-1.147931625	1.71E-14	4.31E-14	down
CCDC126	-1.14779001	3.82E-09	8.02E-09	down
HMGCR	-1.147172815	3.78E-213	1.29E-211	down
MTCP1	-1.147004443	0.00113752	0.00179004	down
RGPD8	-1.147004443	4.18E-06	7.63E-06	down
WDSUB1	-1.147004443	3.43E-13	8.28E-13	down
ZNF69	-1.147004443	0.00113752	0.00179004	down
ANTXR1	-1.146439234	5.58E-91	5.59E-90	down
NFE2L2	-1.146393074	5.49E-123	8.14E-122	down
GLO1	-1.14606448	0	0	down
PXMP4	-1.145819802	2.98E-24	9.58E-24	down
TPP2	-1.145703252	1.22E-74	9.91E-74	down
AHI1	-1.145355648	2.77E-23	8.73E-23	down
COL4A3BP	-1.144978307	6.59E-53	3.78E-52	down
PPTC7	-1.144443445	2.41E-43	1.17E-42	down
CTTNBP2NL	-1.144003041	4.59E-47	2.39E-46	down
WIPI1	-1.143464085	1.68E-06	3.12E-06	down
PAWR	-1.142882001	2.84E-113	3.77E-112	down
FAM102B	-1.142689206	2.12E-17	5.79E-17	down
PURB	-1.142507841	2.33E-82	2.09E-81	down
ZFX	-1.142118915	6.11E-38	2.67E-37	down

ZMPSTE24	-1.142033056	6.12E-132	9.94E-131	down
MED21	-1.141973743	5.14E-48	2.73E-47	down
PPP6R3	-1.1418878	4.38E-166	1.03E-164	down
ZNF41	-1.141815605	1.99E-16	5.28E-16	down
MAP1B	-1.13960912	1.29E-42	6.19E-42	down
LATS1	-1.139167647	5.04E-44	2.48E-43	down
ZNF793	-1.138830511	1.27E-07	2.49E-07	down
ZNF281	-1.13879176	1.09E-114	1.48E-113	down
HPF1	-1.138549476	2.91E-17	7.90E-17	down
ZCCHC8	-1.138471548	3.46E-29	1.25E-28	down
DYM	-1.138369218	1.39E-51	7.78E-51	down
ING2	-1.138369218	1.67E-13	4.07E-13	down
ROCK1	-1.138231617	5.12E-124	7.70E-123	down
ENTPD1	-1.137102585	6.72E-06	1.21E-05	down
XRCC2	-1.136997742	2.44E-30	9.03E-30	down
TAPT1	-1.136116127	2.57E-15	6.63E-15	down
NIPSNAP2	-1.135889264	1.24E-80	1.09E-79	down
ZBED5	-1.135433367	3.38E-32	1.31E-31	down
XPA	-1.135244997	8.97E-18	2.47E-17	down
EIF4E	-1.135231851	6.65E-33	2.61E-32	down
SUB1	-1.134972094	3.80E-259	1.74E-257	down
ERO1A	-1.134807993	8.23E-117	1.15E-115	down
USP34	-1.134758644	1.04E-247	4.48E-246	down
DDX1	-1.134370984	1.60E-112	2.12E-111	down
ZNF83	-1.134260258	1.47E-29	5.35E-29	down
PIK3R3	-1.133780232	6.15E-12	1.42E-11	down
UFSP2	-1.133683064	4.42E-08	8.85E-08	down
RFK	-1.133487831	3.23E-58	2.02E-57	down
DARS2	-1.133285901	2.33E-72	1.84E-71	down
BNIP2	-1.133238448	2.51E-60	1.62E-59	down
ZNF510	-1.132556399	1.35E-26	4.56E-26	down
KIAA0040	-1.131950097	6.84E-05	0.00011648	down
ELF1	-1.131853001	1.58E-40	7.25E-40	down
MYBL1	-1.131592064	2.82E-33	1.11E-32	down
COPB1	-1.131509371	1.18E-163	2.70E-162	down
ACTR2	-1.131478782	0	0	down
ZSCAN29	-1.131243067	1.23E-17	3.37E-17	down
SLC30A6	-1.131183783	4.25E-36	1.78E-35	down
EIF2AK2	-1.130526619	2.18E-91	2.19E-90	down
HSDL2	-1.130006091	2.53E-94	2.65E-93	down
ITGB8	-1.129963274	1.24E-37	5.36E-37	down
ZNF613	-1.129930929	4.33E-07	8.31E-07	down
AK3	-1.129181672	2.27E-23	7.14E-23	down
GNAI3	-1.128952115	1.07E-203	3.40E-202	down
USP12	-1.128869957	3.04E-55	1.80E-54	down
ZNF525	-1.12879091	4.76E-23	1.49E-22	down
MFF	-1.128090471	1.74E-71	1.35E-70	down
USP8	-1.127981195	8.48E-50	4.64E-49	down
MAGI3	-1.127439658	6.53E-25	2.13E-24	down
SUPT7L	-1.127354608	9.70E-28	3.38E-27	down
FSD2	-1.126655797	0.00185894	0.00287999	down
SPRYD7	-1.126655797	3.54E-17	9.59E-17	down
SRGAP2C	-1.126345907	5.18E-31	1.95E-30	down
ATP6AP2	-1.126114144	1.52E-235	6.01E-234	down

TBK1	-1.126076441	1.06E-48	5.70E-48	down
CAPN7	-1.125988963	4.01E-56	2.42E-55	down
AEBP2	-1.125878274	1.01E-36	4.31E-36	down
ERLEC1	-1.125828327	1.92E-67	1.40E-66	down
RCN2	-1.125640887	3.94E-73	3.13E-72	down
DCDC2	-1.125025326	4.75E-35	1.95E-34	down
MFSD14A	-1.124532625	1.01E-18	2.84E-18	down
KRCC1	-1.124341932	2.33E-05	4.08E-05	down
TBCK	-1.124341932	2.33E-05	4.08E-05	down
WDR19	-1.124062421	4.15E-19	1.18E-18	down
LRPPRC	-1.123588086	0	0	down
ZNF75D	-1.123044544	3.93E-14	9.77E-14	down
YTHDF3	-1.122455809	6.38E-79	5.51E-78	down
DNM1L	-1.121665106	1.53E-129	2.42E-128	down
KIAA1328	-1.121637723	6.92E-07	1.31E-06	down
RNGTT	-1.12157588	2.11E-58	1.33E-57	down
TMEM14A	-1.121362793	3.28E-27	1.13E-26	down
CNEP1R1	-1.120606753	1.79E-25	5.90E-25	down
CTDSPL2	-1.120162035	1.74E-51	9.73E-51	down
FANCI	-1.119802126	2.73E-106	3.34E-105	down
MBNL1	-1.119001636	7.18E-163	1.62E-161	down
SPCS3	-1.118911228	1.64E-157	3.50E-156	down
SLC35A1	-1.11879368	1.29E-17	3.53E-17	down
C9orf64	-1.118010906	4.27E-15	1.09E-14	down
TGFBR2	-1.117979288	3.13E-89	3.06E-88	down
FAM200A	-1.117456861	2.40E-07	4.67E-07	down
TRIM24	-1.117433506	2.98E-47	1.55E-46	down
GPD1L	-1.116759761	6.61E-46	3.37E-45	down
SPIN1	-1.116214051	1.06E-162	2.39E-161	down
ZNF655	-1.116144779	3.27E-71	2.53E-70	down
NUP54	-1.116045403	8.09E-59	5.11E-58	down
CHD6	-1.115996679	4.96E-46	2.53E-45	down
PGRMC2	-1.115535553	3.99E-76	3.33E-75	down
STT3B	-1.115355624	2.02E-222	7.25E-221	down
USP24	-1.115328014	2.07E-180	5.55E-179	down
PDE7A	-1.114873567	5.37E-16	1.41E-15	down
ATP2C1	-1.114861313	2.28E-106	2.79E-105	down
TRAPPC6B	-1.114260251	1.63E-27	5.66E-27	down
MSRB3	-1.114028189	1.00E-46	5.17E-46	down
HMGB1	-1.113773288	1.41E-293	7.70E-292	down
LEMD3	-1.113159955	2.88E-54	1.69E-53	down
AMMECR1	-1.112884678	3.66E-26	1.23E-25	down
ATF7IP	-1.112646493	1.66E-89	1.62E-88	down
NSUN3	-1.112347961	5.53E-09	1.15E-08	down
PIGF	-1.112347961	1.23E-14	3.10E-14	down
ZNF34	-1.112347961	0.00355495	0.00537054	down
DHRS7	-1.111837588	2.22E-27	7.68E-27	down
ATL2	-1.11096984	4.08E-68	3.00E-67	down
BIRC6	-1.110920371	2.28E-86	2.14E-85	down
ZFPM2	-1.110888482	1.51E-10	3.32E-10	down
HMMR	-1.110837527	1.32E-107	1.63E-106	down
WASHC2C	-1.110666066	2.71E-50	1.50E-49	down
NBPF11	-1.110508375	1.18E-08	2.42E-08	down
LEPR	-1.110380258	2.83E-28	1.00E-27	down

GNG12	-1.110352822	6.13E-258	2.79E-256	down
MAGOHB	-1.109860555	2.07E-17	5.66E-17	down
MARK1	-1.109860555	9.51E-07	1.79E-06	down
PPP3CA	-1.10947816	9.10E-44	4.45E-43	down
TSPAN12	-1.108936402	4.74E-14	1.18E-13	down
TPP1	-1.108016898	5.16E-70	3.91E-69	down
CCDC138	-1.107658807	2.61E-18	7.28E-18	down
CTNND1	-1.10764671	7.79E-239	3.16E-237	down
NAP1L1	-1.107196787	0	0	down
RBM26	-1.106785208	5.81E-56	3.50E-55	down
GRAMD1C	-1.106650106	5.18E-13	1.24E-12	down
HELZ	-1.106425273	5.84E-39	2.60E-38	down
SP140L	-1.105963402	3.56E-09	7.47E-09	down
CNOT2	-1.105897237	6.23E-49	3.35E-48	down
CISD2	-1.105865748	1.04E-33	4.14E-33	down
FAM184A	-1.105491187	1.61E-11	3.68E-11	down
NSMAF	-1.105451953	3.09E-32	1.20E-31	down
SNRNP48	-1.105006367	6.67E-45	3.32E-44	down
CETN2	-1.104617685	6.98E-47	3.62E-46	down
CCL28	-1.103935721	6.99E-05	0.00011903	down
PLEKHA3	-1.103935721	8.22E-28	2.87E-27	down
SMIM13	-1.103935721	1.05E-44	5.20E-44	down
TEC	-1.103935721	0.00116688	0.00183455	down
ZNF431	-1.103935721	3.56E-18	9.87E-18	down
MIOS	-1.102914344	8.80E-32	3.36E-31	down
KCTD3	-1.102722658	6.06E-65	4.25E-64	down
TDP2	-1.102322148	3.08E-49	1.67E-48	down
PBX3	-1.101276374	2.94E-36	1.24E-35	down
TSHZ1	-1.100908028	1.52E-06	2.83E-06	down
AGTPBP1	-1.100865859	1.64E-56	9.98E-56	down
HAUS3	-1.10030447	9.78E-23	3.04E-22	down
ZNF235	-1.100020671	2.38E-05	4.16E-05	down
LRR1	-1.099819612	2.17E-31	8.22E-31	down
CCDC198	-1.099596785	6.01E-05	0.00010258	down
EXOSC8	-1.099596785	1.93E-47	1.01E-46	down
RDH11	-1.099476073	1.92E-56	1.17E-55	down
FEZ2	-1.099301647	1.18E-57	7.30E-57	down
CEP192	-1.099197802	1.24E-59	7.95E-59	down
RPF2	-1.098802874	2.77E-43	1.34E-42	down
HMGXB4	-1.098641421	7.00E-45	3.48E-44	down
STX2	-1.098504757	1.38E-60	9.00E-60	down
ANKRD46	-1.097835521	4.86E-09	1.01E-08	down
KIN	-1.096282148	1.64E-11	3.74E-11	down
SENP8	-1.096282148	0.00259447	0.00396509	down
UNC50	-1.095761789	2.33E-18	6.51E-18	down
2-9月	-1.095569492	0	0	down
SAMD8	-1.095474142	2.95E-48	1.57E-47	down
CSTF2T	-1.094806931	3.06E-23	9.60E-23	down
RNF170	-1.094475392	3.46E-11	7.81E-11	down
MAGED1	-1.094451317	1.26E-149	2.48E-148	down
C1RL	-1.093667385	7.01E-06	1.26E-05	down
SYF2	-1.093304169	5.55E-23	1.73E-22	down
CLK1	-1.092895914	8.83E-47	4.57E-46	down
BICRAL	-1.092769803	1.19E-24	3.87E-24	down

EDEM1	-1.092565528	1.42E-70	1.09E-69	down
ACP6	-1.092440082	9.67E-07	1.82E-06	down
GPATCH11	-1.092440082	6.43E-28	2.26E-27	down
COX11	-1.091654566	4.95E-47	2.57E-46	down
FAM221A	-1.091551996	0.01838619	0.02592444	down
GKAP1	-1.091551996	4.43E-05	7.63E-05	down
SBF2	-1.091551996	2.71E-33	1.07E-32	down
GUF1	-1.091272486	5.90E-35	2.42E-34	down
SIX1	-1.090879568	1.90E-08	3.86E-08	down
YIPF5	-1.09037327	3.97E-37	1.70E-36	down
GNPNAT1	-1.090179287	3.10E-106	3.79E-105	down
PLPP6	-1.090129921	0.00011216	0.00018881	down
ZNF146	-1.090041279	4.46E-118	6.30E-117	down
RNLS	-1.089288945	0.00221628	0.00341082	down
CPOX	-1.089207384	2.85E-35	1.18E-34	down
ESF1	-1.089052438	5.55E-48	2.94E-47	down
BCKDHB	-1.088587757	2.95E-17	8.01E-17	down
BOLA2-SMG1	-1.088338866	3.30E-10	7.18E-10	down
NUCKS1	-1.088196523	0	0	down
OSTC	-1.086568589	9.28E-38	4.03E-37	down
ZBED6CL	-1.086013813	0.00073176	0.00116627	down
FZD6	-1.085774936	3.38E-85	3.13E-84	down
RAB5A	-1.084677365	1.94E-46	9.97E-46	down
IFNAR1	-1.084489665	1.06E-75	8.80E-75	down
RDX	-1.084063921	3.68E-178	9.62E-177	down
C5orf30	-1.084036163	4.62E-15	1.18E-14	down
RANBP2	-1.083806566	1.47E-154	3.04E-153	down
TRMT10C	-1.083587075	1.51E-23	4.78E-23	down
TBC1D9	-1.083448369	9.25E-20	2.67E-19	down
TRIM13	-1.083236728	3.67E-23	1.15E-22	down
DGLUCY	-1.082874105	0.00189321	0.00293042	down
EHHADH	-1.082874105	1.12E-05	1.99E-05	down
HYKK	-1.082874105	0.00189321	0.00293042	down
ZNF266	-1.082874105	5.47E-46	2.79E-45	down
MIS12	-1.082591902	1.87E-38	8.27E-38	down
TPRKB	-1.08249855	2.16E-22	6.65E-22	down
ZC3H7A	-1.082463158	1.37E-83	1.24E-82	down
DNAJC24	-1.081909415	9.14E-18	2.51E-17	down
ZNF652	-1.08176434	6.40E-11	1.43E-10	down
MPDZ	-1.081724442	5.66E-31	2.12E-30	down
TTLL7	-1.081468381	2.61E-29	9.43E-29	down
ANKRA2	-1.080852108	1.32E-06	2.47E-06	down
USP46	-1.080648868	1.39E-22	4.32E-22	down
TCERG1	-1.08019027	7.19E-117	1.01E-115	down
PIAS1	-1.079798815	8.22E-22	2.50E-21	down
ANKRD44	-1.079061052	4.76E-11	1.07E-10	down
GLIS3	-1.079061052	3.30E-06	6.04E-06	down
COX20	-1.078615748	3.26E-45	1.63E-44	down
ZNF611	-1.078607541	9.84E-17	2.63E-16	down
KIF5B	-1.078576	6.68E-250	2.90E-248	down
ZXDA	-1.078400629	0.00495991	0.0073906	down
N4BP1	-1.078329065	2.82E-45	1.41E-44	down
DNAJC21	-1.077164492	1.11E-89	1.09E-88	down
CKAP2L	-1.077050909	1.35E-61	8.94E-61	down

OXTR	-1.076522364	3.27E-18	9.08E-18	down
RABL3	-1.076522364	3.27E-18	9.08E-18	down
SBNO1	-1.075653644	2.59E-104	3.09E-103	down
CEP78	-1.074844822	7.77E-55	4.59E-54	down
IGSF10	-1.074332008	6.08E-10	1.31E-09	down
BTBD10	-1.074134943	3.53E-24	1.13E-23	down
PARG	-1.073998437	1.18E-39	5.34E-39	down
AMD1	-1.073849574	1.50E-132	2.47E-131	down
TM9SF2	-1.073750633	4.66E-133	7.70E-132	down
ZNF860	-1.073735483	8.41E-07	1.59E-06	down
SLC38A2	-1.073291765	0	0	down
CAMSAP2	-1.07325833	2.76E-89	2.69E-88	down
ARID4B	-1.073056408	7.64E-40	3.45E-39	down
HIPK2	-1.072409215	2.26E-80	1.97E-79	down
SOWAHC	-1.071827226	3.34E-39	1.49E-38	down
BORCS7	-1.071514243	1.78E-05	3.14E-05	down
MCF2L	-1.071514243	0.04308505	0.05789774	down
TCEANC	-1.071514243	0.04308505	0.05789774	down
ZNF44	-1.071514243	0.00015403	0.0002569	down
HMGA2	-1.07120268	2.49E-39	1.12E-38	down
CPD	-1.071153389	2.50E-131	4.05E-130	down
LRRC1	-1.070844934	3.58E-19	1.02E-18	down
ZNF776	-1.070789453	8.02E-18	2.21E-17	down
CEP55	-1.070352329	2.42E-132	3.97E-131	down
ENOX2	-1.069574702	9.48E-20	2.74E-19	down
GALNT1	-1.06952569	2.71E-66	1.94E-65	down
CMAS	-1.069458913	1.69E-41	7.90E-41	down
FRYL	-1.069456353	8.71E-53	4.98E-52	down
OSTM1	-1.069337059	2.90E-39	1.30E-38	down
COCH	-1.068841966	1.20E-36	5.11E-36	down
CWC15	-1.068698541	7.40E-35	3.03E-34	down
OSBPL1A	-1.068589531	1.33E-37	5.75E-37	down
SOAT1	-1.068394608	1.49E-87	1.41E-86	down
MAD2L1	-1.067715533	8.42E-109	1.06E-107	down
MEF2A	-1.067567059	3.74E-34	1.51E-33	down
TNKS2	-1.067154786	4.85E-63	3.30E-62	down
INIP	-1.066883577	2.10E-49	1.14E-48	down
USP38	-1.066822548	1.77E-26	5.96E-26	down
NT5C3A	-1.065607602	5.54E-41	2.56E-40	down
RAD52	-1.065255253	7.54E-11	1.68E-10	down
ZNF527	-1.064858199	1.66E-08	3.39E-08	down
DHX29	-1.06383521	6.87E-62	4.59E-61	down
RLF	-1.063778594	1.36E-31	5.17E-31	down
SUPT20H	-1.063427128	7.62E-29	2.73E-28	down
CBWD3	-1.063293736	0.00033608	0.00054765	down
NEK11	-1.063293736	0.00033608	0.00054765	down
ARMC8	-1.063180179	1.42E-33	5.64E-33	down
MAN2A1	-1.062870472	2.00E-44	9.91E-44	down
PEX13	-1.062766532	5.31E-36	2.23E-35	down
LZIC	-1.062390829	1.04E-25	3.45E-25	down
USPL1	-1.062390829	1.04E-25	3.45E-25	down
SLMAP	-1.06223643	1.35E-83	1.23E-82	down
PQLC2L	-1.061766267	0.0010085	0.00159282	down
ATG5	-1.061707485	4.16E-38	1.82E-37	down

PRPF4B	-1.061262681	3.51E-99	3.93E-98	down
ZNF318	-1.06117515	1.24E-30	4.62E-30	down
BLOC1S2	-1.061122637	1.32E-21	3.98E-21	down
PURA	-1.0596915	1.23E-36	5.20E-36	down
ABCA10	-1.059541602	0.00306891	0.00466361	down
C2orf74	-1.059541602	0.00306891	0.00466361	down
PKD2	-1.059541602	5.55E-17	1.49E-16	down
STK17B	-1.059541602	1.21E-25	4.00E-25	down
TMEM169	-1.059541602	0.03629873	0.04934243	down
AFF4	-1.059228319	3.19E-168	7.74E-167	down
DPP8	-1.05917834	1.67E-30	6.20E-30	down
MYCBP	-1.058847831	2.68E-24	8.62E-24	down
URI1	-1.058506689	1.85E-97	2.02E-96	down
MCCC1	-1.058101067	3.82E-16	1.00E-15	down
SMARCA5	-1.058092884	1.55E-127	2.39E-126	down
C4orf3	-1.058068712	5.95E-23	1.86E-22	down
MAP3K13	-1.057999437	3.53E-15	9.06E-15	down
TTC26	-1.057393135	4.31E-21	1.29E-20	down
SELENOF	-1.057015457	9.44E-148	1.83E-146	down
INTS13	-1.055903024	3.19E-70	2.42E-69	down
SLC44A1	-1.055869062	4.73E-78	4.04E-77	down
PPAT	-1.055837633	2.07E-63	1.42E-62	down
CMTM6	-1.055308024	4.23E-113	5.62E-112	down
LCA5	-1.05502612	1.94E-08	3.94E-08	down
ASAH1	-1.054692202	5.01E-53	2.87E-52	down
GOLGA1	-1.054506012	2.07E-26	6.97E-26	down
MDM4	-1.054390065	6.91E-31	2.59E-30	down
LYST	-1.054304953	2.04E-29	7.41E-29	down
MFSD1	-1.053121931	1.37E-43	6.67E-43	down
SNX7	-1.053042569	1.53E-60	9.92E-60	down
ATP6V1A	-1.052489417	1.11E-99	1.25E-98	down
UBXN7	-1.052073992	2.05E-60	1.33E-59	down
TSGA10	-1.05184721	1.78E-05	3.15E-05	down
MPP5	-1.051622938	3.70E-77	3.13E-76	down
ZNF674	-1.051468301	3.59E-11	8.08E-11	down
ZIK1	-1.05136767	1.55E-06	2.89E-06	down
ZNF45	-1.051143353	7.61E-19	2.15E-18	down
ZBTB11	-1.050797352	2.52E-52	1.43E-51	down
SELENOI	-1.050384076	1.72E-54	1.01E-53	down
BNIP3L	-1.049888519	1.90E-72	1.49E-71	down
ATG12	-1.049713941	1.58E-43	7.72E-43	down
PAIP2	-1.049682962	3.71E-50	2.04E-49	down
IL18R1	-1.049487937	0.00223022	0.00343102	down
MCOLN2	-1.049487937	1.06E-08	2.19E-08	down
MT1F	-1.049487937	0.00809529	0.01182893	down
PPP1R3D	-1.049487937	3.96E-07	7.62E-07	down
LRP1	-1.048930158	1.38E-18	3.85E-18	down
SMAD1	-1.048831121	5.14E-16	1.35E-15	down
PTPRE	-1.048570484	6.95E-12	1.61E-11	down
DCK	-1.048181739	3.38E-52	1.92E-51	down
MRPL19	-1.048171409	4.40E-66	3.14E-65	down
SKP2	-1.048160914	1.42E-65	1.01E-64	down
TRMT11	-1.047468769	1.25E-15	3.25E-15	down
KIF16B	-1.047352192	2.70E-19	7.71E-19	down

ZSCAN9	-1.047352192	2.15E-10	4.73E-10	down
TMED8	-1.046848055	5.44E-48	2.88E-47	down
PAPLN	-1.045903599	2.04E-09	4.32E-09	down
ZNF684	-1.045903599	9.84E-07	1.85E-06	down
ZNF326	-1.045808529	5.91E-40	2.67E-39	down
PARD6B	-1.045640446	3.82E-43	1.84E-42	down
CCNE2	-1.045371828	2.63E-15	6.77E-15	down
UBL3	-1.042945091	1.81E-14	4.54E-14	down
ADSL	-1.042787065	1.67E-08	3.40E-08	down
CNOT7	-1.042710868	2.00E-81	1.77E-80	down
DNAAF2	-1.042634266	2.33E-19	6.66E-19	down
NSUN6	-1.042535176	0.00045979	0.00074294	down
FAM217B	-1.04249535	1.07E-45	5.42E-45	down
TAF2	-1.041582739	2.18E-93	2.25E-92	down
EXOC6	-1.041243943	3.53E-15	9.06E-15	down
PPP1R2	-1.041237689	6.47E-57	3.96E-56	down
HSPE1	-1.041199966	2.29E-17	6.23E-17	down
ADSS	-1.040291911	3.85E-49	2.08E-48	down
PSMD10	-1.040012073	1.07E-48	5.76E-48	down
SRSF1	-1.039805383	6.97E-86	6.50E-85	down
OXSR1	-1.039730265	2.80E-66	2.01E-65	down
RPRD1A	-1.039684343	8.69E-102	1.01E-100	down
UTP20	-1.039533584	7.55E-131	1.21E-129	down
EREG	-1.039421841	8.33E-147	1.60E-145	down
AASS	-1.03936372	2.26E-15	5.83E-15	down
STX17	-1.03936372	2.26E-15	5.83E-15	down
USP49	-1.03936372	8.27E-10	1.78E-09	down
HNRNPLL	-1.039165465	7.71E-53	4.41E-52	down
THAP12	-1.039119619	1.13E-84	1.04E-83	down
GFPT1	-1.038892189	2.77E-73	2.22E-72	down
GPATCH2L	-1.038286041	4.20E-47	2.19E-46	down
EBAG9	-1.038167951	3.56E-17	9.62E-17	down
SMARCA2	-1.038119123	7.97E-14	1.96E-13	down
SSBP2	-1.03763335	2.42E-05	4.23E-05	down
MCFD2	-1.037477073	1.53E-205	4.91E-204	down
FAM169A	-1.036645632	1.28E-19	3.69E-19	down
PLA2G12A	-1.036234228	1.94E-13	4.73E-13	down
SLC11A2	-1.035862116	1.18E-49	6.42E-49	down
PPP4R3B	-1.035813	1.68E-106	2.06E-105	down
PLEKHA8	-1.035351454	3.11E-19	8.87E-19	down
CREB1	-1.035222971	4.64E-30	1.71E-29	down
MRPL50	-1.034537595	1.20E-25	3.98E-25	down
AP4S1	-1.033546393	7.11E-05	0.00012099	down
CRYBG3	-1.033546393	7.19E-30	2.64E-29	down
PLXNC1	-1.033546393	0.02181586	0.03046885	down
SLC24A1	-1.033546393	0.00496917	0.00740354	down
VASH2	-1.033546393	0.00028736	0.00047006	down
ZNF547	-1.033546393	0.02181586	0.03046885	down
ZNF717	-1.033546393	0.00028736	0.00047006	down
UTP15	-1.033355926	3.74E-43	1.80E-42	down
FAM200B	-1.033227882	1.77E-26	5.96E-26	down
CDC42BPA	-1.032584276	3.52E-116	4.86E-115	down
KLF3	-1.032390619	5.64E-29	2.02E-28	down
CA10	-1.032042802	9.81E-07	1.85E-06	down

HDGFL3	-1.031858043	2.40E-34	9.74E-34	down
SIAH1	-1.031621516	2.07E-34	8.43E-34	down
SYPL1	-1.031542563	4.65E-127	7.16E-126	down
ARHGEF12	-1.031466739	5.95E-243	2.47E-241	down
NAA15	-1.031419308	4.72E-105	5.69E-104	down
SORL1	-1.031307921	3.80E-12	8.86E-12	down
ZNF506	-1.031307921	1.43E-08	2.92E-08	down
MINDY3	-1.030872259	2.42E-16	6.41E-16	down
DENND2C	-1.030583979	8.42E-07	1.59E-06	down
OTUD1	-1.030226035	0.00100723	0.0015914	down
CPSF3	-1.029949463	1.15E-49	6.29E-49	down
CCDC117	-1.029794258	1.39E-45	7.03E-45	down
ACTRT3	-1.029167952	5.22E-05	8.95E-05	down
UST	-1.029167952	0.00422603	0.00633855	down
AK4	-1.028620364	6.27E-70	4.73E-69	down
UPF2	-1.027382218	1.48E-29	5.39E-29	down
LCA5L	-1.027120124	0.01843953	0.02599678	down
WSB1	-1.026594125	7.86E-78	6.69E-77	down
PDSS2	-1.026039858	4.04E-15	1.03E-14	down
PHTF1	-1.025054932	1.45E-24	4.69E-24	down
MND1	-1.024613268	2.87E-10	6.27E-10	down
CSPP1	-1.024208528	4.34E-14	1.08E-13	down
TIGD2	-1.024208528	1.79E-06	3.33E-06	down
KCNJ2	-1.023647932	8.67E-08	1.72E-07	down
OMA1	-1.023647932	5.54E-11	1.24E-10	down
C12orf73	-1.023015725	7.07E-06	1.27E-05	down
USP28	-1.022935999	6.81E-39	3.03E-38	down
PSMC6	-1.022557368	1.39E-70	1.06E-69	down
FNIP1	-1.022297252	6.45E-43	3.10E-42	down
SSB	-1.022192571	3.80E-77	3.22E-76	down
FAM151B	-1.021473561	0.01559696	0.0221336	down
RNF6	-1.021473561	5.83E-42	2.75E-41	down
ZNF821	-1.021473561	0.00013155	0.00022044	down
SGK3	-1.020519713	1.22E-08	2.50E-08	down
TADA1	-1.020310566	4.08E-19	1.16E-18	down
DYNC1LI2	-1.019766734	4.44E-128	6.92E-127	down
PICK1	-1.019740593	2.41E-05	4.20E-05	down
KCTD20	-1.019601588	5.60E-81	4.95E-80	down
RIC1	-1.019402211	2.25E-11	5.12E-11	down
ZADH2	-1.019342773	7.24E-24	2.31E-23	down
BIRC2	-1.01907029	1.45E-116	2.01E-115	down
CEPT1	-1.019059375	1.91E-32	7.43E-32	down
IQCK	-1.018899617	0.00053498	0.00086132	down
P2RX5	-1.018899617	0.00053498	0.00086132	down
LSM11	-1.018402907	4.06E-08	8.14E-08	down
ABHD10	-1.018119754	6.98E-50	3.83E-49	down
ABCB10	-1.018013398	9.90E-37	4.21E-36	down
NCBP1	-1.018009705	1.45E-133	2.42E-132	down
MAPK8	-1.017689215	8.06E-41	3.71E-40	down
ARHGAP26	-1.017668648	1.42E-17	3.89E-17	down
NT5DC1	-1.017463154	6.35E-37	2.71E-36	down
ZNF367	-1.017240393	5.42E-43	2.61E-42	down
CREB3L2	-1.017092461	2.19E-32	8.51E-32	down
RNF168	-1.016698002	7.93E-30	2.91E-29	down

C18orf32	-1.01647288	0.01320156	0.01888772	down
PARD3B	-1.01647288	0.01320156	0.01888772	down
TRAM1L1	-1.01647288	0.01320156	0.01888772	down
CDC73	-1.016402571	1.45E-91	1.46E-90	down
GNPAT	-1.016103722	1.27E-61	8.43E-61	down
OBSL1	-1.015952333	1.36E-07	2.66E-07	down
TUT7	-1.015735117	2.14E-27	7.40E-27	down
MED4	-1.015384016	1.33E-33	5.27E-33	down
CPEB4	-1.015365414	6.68E-16	1.75E-15	down
TP53BP1	-1.015268411	2.13E-38	9.39E-38	down
BRCC3	-1.014991293	8.86E-36	3.70E-35	down
SMAD2	-1.014930715	1.10E-97	1.20E-96	down
CYLD	-1.014698565	3.00E-37	1.29E-36	down
ZFP82	-1.014010041	1.56E-10	3.43E-10	down
ARMC1	-1.013979024	3.76E-47	1.96E-46	down
AKTIP	-1.013934264	4.95E-14	1.23E-13	down
RPGR	-1.013934264	4.95E-14	1.23E-13	down
ARAP2	-1.013237411	2.59E-11	5.87E-11	down
CDADC1	-1.01308229	4.54E-07	8.69E-07	down
ZNF250	-1.012962061	8.59E-08	1.70E-07	down
BCLAF1	-1.012692763	8.91E-185	2.49E-183	down
CCNL1	-1.012133944	7.38E-51	4.10E-50	down
ADPRH	-1.012013231	0.01118118	0.01613927	down
DPYD	-1.012013231	7.74E-18	2.13E-17	down
GNGT1	-1.012013231	0.01118118	0.01613927	down
NEK8	-1.012013231	0.00188894	0.00292433	down
SASH1	-1.012013231	2.97E-19	8.47E-19	down
ZNF791	-1.012013231	9.92E-11	2.20E-10	down
BTRC	-1.011690083	5.03E-20	1.46E-19	down
FADS1	-1.011422832	6.53E-112	8.62E-111	down
HNRNPA3	-1.011273063	5.31E-249	2.30E-247	down
FAM206A	-1.011051755	3.73E-20	1.09E-19	down
CDKL5	-1.010972701	3.34E-07	6.45E-07	down
RAB21	-1.010696663	1.19E-46	6.16E-46	down
ZW10	-1.010552274	2.73E-50	1.51E-49	down
RIC8B	-1.010438666	1.11E-16	2.97E-16	down
ERBIN	-1.010409184	3.98E-123	5.91E-122	down
GRIP1	-1.010361606	5.17E-05	8.87E-05	down
SGTB	-1.010263753	2.35E-25	7.73E-25	down
DHX57	-1.010102669	6.31E-42	2.97E-41	down
CREBZF	-1.010099335	1.96E-63	1.35E-62	down
C12orf75	-1.01009571	1.31E-75	1.08E-74	down
RBPJ	-1.010016746	5.15E-52	2.90E-51	down
NXPE3	-1.009867397	1.67E-48	8.90E-48	down
TMEM184C	-1.009462948	7.64E-25	2.48E-24	down
TOM1L1	-1.009024074	3.83E-43	1.84E-42	down
ZBTB44	-1.008845952	1.14E-68	8.47E-68	down
PCNX4	-1.008435561	4.33E-74	3.49E-73	down
MALT1	-1.008415486	6.89E-64	4.77E-63	down
LRBA	-1.007667761	3.38E-34	1.36E-33	down
HTATSF1	-1.007370673	2.73E-71	2.11E-70	down
ASF1A	-1.007347331	3.12E-14	7.79E-14	down
KIF1BP	-1.006925252	7.07E-48	3.73E-47	down
ANKEF1	-1.006767062	2.96E-08	5.98E-08	down

IPP	-1.006767062	1.39E-19	4.01E-19	down
ZC3H6	-1.006160505	0.00020809	0.00034413	down
NUFIP2	-1.006133582	2.84E-102	3.30E-101	down
ANLN	-1.005117985	1.42E-287	7.53E-286	down
TRIM52	-1.004832057	7.58E-14	1.87E-13	down
RNF182	-1.004629794	1.53E-70	1.17E-69	down
TMED10	-1.004211817	2.09E-141	3.76E-140	down
PELI1	-1.004136806	9.67E-62	6.45E-61	down
TMEM246	-1.003998238	9.42E-15	2.38E-14	down
ADAM17	-1.003912733	2.01E-12	4.74E-12	down
WDR75	-1.003774737	1.96E-77	1.66E-76	down
CLTC	-1.003367968	0	0	down
RALGAPB	-1.003334541	2.53E-100	2.88E-99	down
GRTP1	-1.003052364	2.38E-05	4.16E-05	down
HOXB6	-1.00272347	0.00015208	0.00025374	down
JAK2	-1.00272347	8.48E-08	1.68E-07	down
DCAF16	-1.002563389	9.26E-42	4.34E-41	down
ABHD18	-1.002182222	1.19E-08	2.45E-08	down
ZNF460	-1.002182222	0.00099683	0.00157535	down
SNAPC3	-1.002066315	6.00E-31	2.25E-30	down
CDC42SE2	-1.001755326	2.65E-49	1.43E-48	down
ZEB2	-1.001755326	2.04E-05	3.58E-05	down
STPG1	-1.001473687	1.02E-08	2.11E-08	down
ZNF641	-1.001273946	1.97E-18	5.50E-18	down
MBD4	-1.001124915	1.67E-30	6.23E-30	down
PPIP5K1	-1.001124915	3.41E-11	7.71E-11	down
HIBADH	-1.000865694	6.08E-21	1.81E-20	down
NDFIP2	-1.000657646	2.02E-60	1.31E-59	down
EIF5B	-1.000506131	1.49E-166	3.52E-165	down
PLCXD2	-1.000389559	1.26E-18	3.53E-18	down
CEP128	-1.00009991	8.69E-14	2.14E-13	down
NPNT	-1.00009991	3.46E-38	1.52E-37	down

AccID	log2FC	Pvalue	FDR	Style
FOXP4	1.00121243	2.53E-42	1.20E-41	up
NOP56	1.00160488	8.43E-283	4.41E-281	up
RABEP2	1.0016683	3.64E-14	9.06E-14	up
PRR19	1.00185894	0.00560676	0.00832043	up
RPL38	1.00203392	8.07E-111	1.05E-109	up
BLVRB	1.0021428	1.62E-18	4.52E-18	up
PHPT1	1.00274268	1.64E-31	6.23E-31	up
PXDN	1.00305802	1.29E-13	3.16E-13	up
PTK2B	1.00325353	7.31E-05	0.0001242	up
TPPP	1.00325353	2.04E-08	4.14E-08	up
RBM10	1.00440148	2.48E-75	2.05E-74	up
RAPGEF1	1.00458874	2.59E-120	3.74E-119	up
MBD6	1.0053265	2.03E-20	5.97E-20	up
NUDT14	1.00544774	7.21E-07	1.37E-06	up
THEM6	1.0055417	6.87E-26	2.28E-25	up
TMEM129	1.00561666	1.28E-19	3.69E-19	up
CCS	1.00572876	1.16E-09	2.49E-09	up
ACTN1	1.0059548	0	0	up
SGF29	1.0066921	1.34E-12	3.18E-12	up
LAMA2	1.00709559	0.00366566	0.00552731	up
CDK9	1.0079096	1.95E-64	1.36E-63	up
C1orf122	1.00925227	1.36E-29	4.96E-29	up
CFAP45	1.00940575	0.00296721	0.00451391	up
DTX4	1.00940575	0.00296721	0.00451391	up
SPOUT1	1.00940575	3.23E-42	1.53E-41	up
CCDC71	1.00989472	2.40E-10	5.26E-10	up
PIGQ	1.01001845	1.10E-43	5.37E-43	up
TNIP2	1.01027985	4.66E-36	1.96E-35	up
TAOK2	1.01038503	6.95E-50	3.81E-49	up
RPS27	1.01068758	3.08E-45	1.55E-44	up
CSTB	1.01098251	1.91E-53	1.10E-52	up
BSG	1.01123176	0	0	up
ANK1	1.0115415	0.00240343	0.00368726	up
CIRBP	1.01159158	5.09E-43	2.45E-42	up
MRPL2	1.01395858	3.18E-29	1.15E-28	up
BCL2L12	1.01427424	1.20E-49	6.55E-49	up
BYSL	1.01466098	4.73E-65	3.33E-64	up
NSFL1C	1.01549951	2.91E-115	3.97E-114	up
TP53I3	1.0159486	9.84E-05	0.0001661	up
SEMA4B	1.01602082	4.15E-90	4.10E-89	up
RANBP3	1.01620664	4.18E-66	2.99E-65	up
GPAA1	1.01626391	1.27E-70	9.71E-70	up
MRPL38	1.01664082	4.42E-70	3.35E-69	up
C2CD2L	1.01773411	4.06E-13	9.78E-13	up
PEPD	1.01816076	8.94E-75	7.29E-74	up
TIMM29	1.01919507	6.45E-16	1.69E-15	up
PES1	1.02128366	7.13E-134	1.19E-132	up
ZNF524	1.02159516	1.58E-06	2.94E-06	up
TRAF2	1.0222858	4.32E-49	2.33E-48	up
DEAF1	1.02292056	1.07E-10	2.37E-10	up
DNER	1.02292056	0.01467347	0.02087417	up
PLXNA3	1.02304876	8.16E-28	2.85E-27	up
COQ8A	1.02305548	1.64E-26	5.55E-26	up

RNF19B	1.02403396	7.38E-38	3.21E-37	up
SMG5	1.02523796	4.67E-233	1.81E-231	up
CCDC85C	1.0253473	1.21E-26	4.12E-26	up
ASPSCR1	1.02572541	1.83E-18	5.12E-18	up
SSR4	1.02586295	1.09E-81	9.73E-81	up
SHANK1	1.02599121	1.82E-11	4.15E-11	up
EIF5A	1.02654498	0	0	up
PCSK7	1.02670015	1.43E-27	4.97E-27	up
POLR3H	1.02694238	1.51E-59	9.67E-59	up
CLIP2	1.02734043	6.82E-88	6.53E-87	up
CBX8	1.02751513	7.05E-06	1.27E-05	up
SLC26A1	1.02751513	0.00949906	0.01380421	up
MDH2	1.02795493	7.60E-214	2.61E-212	up
ZC3H3	1.02923073	5.58E-14	1.38E-13	up
NSUN5	1.02961689	3.53E-39	1.58E-38	up
WNT7A	1.02976909	5.31E-11	1.19E-10	up
LMAN2	1.03066798	1.49E-80	1.31E-79	up
MRPL57	1.03071381	5.66E-34	2.27E-33	up
MED15	1.03113988	4.99E-67	3.61E-66	up
ABCC2	1.03122386	0.00617691	0.00913251	up
REEP6	1.03122386	4.34E-08	8.68E-08	up
CHPF2	1.03129354	4.07E-41	1.89E-40	up
TMEM222	1.03137553	6.32E-38	2.76E-37	up
RIMKLA	1.03163778	3.56E-08	7.15E-08	up
RPL10	1.03217203	0	0	up
TAGLN	1.03241069	3.31E-27	1.14E-26	up
BX255925.3	1.03301196	1.64E-15	4.25E-15	up
PISD	1.03303288	4.16E-64	2.89E-63	up
GPX4	1.03380168	0	0	up
GCAT	1.03415319	1.78E-21	5.35E-21	up
ASS1	1.03428042	0.00403153	0.00606108	up
EVI5L	1.03473835	1.26E-12	2.99E-12	up
CDKN2D	1.03509527	7.05E-11	1.57E-10	up
DND1	1.03561563	3.17E-05	5.51E-05	up
RNPS1	1.03598171	4.56E-206	1.47E-204	up
CYB5D2	1.036445	2.33E-07	4.53E-07	up
PPP1R37	1.03694075	1.72E-25	5.67E-25	up
EFHD2	1.03714007	2.17E-103	2.56E-102	up
MAPK8IP1	1.03718525	1.02E-35	4.24E-35	up
TRAPPC6A	1.0379749	0.00213795	0.00329503	up
TGM2	1.03837593	0	0	up
DGCR6L	1.0388083	4.10E-42	1.94E-41	up
ANKRD54	1.0389031	5.37E-38	2.35E-37	up
RTL8A	1.03913379	1.78E-40	8.12E-40	up
PTOV1	1.03935415	2.61E-99	2.93E-98	up
GPI	1.03991789	0	0	up
SLC10A3	1.04051056	3.96E-38	1.74E-37	up
PGAM5	1.04073005	1.84E-109	2.34E-108	up
LAMC2	1.04161721	1.07E-298	5.96E-297	up
STIP1	1.04207109	0	0	up
RBM19	1.04249848	3.76E-61	2.48E-60	up
ABCD1	1.04308888	8.77E-12	2.02E-11	up
MARK2	1.04357803	2.36E-43	1.14E-42	up
SERPINE1	1.04378611	2.32E-122	3.42E-121	up

MAF1	1.04399421	1.28E-115	1.76E-114	up
LSR	1.04604629	1.60E-57	9.86E-57	up
SDHA	1.04638558	3.85E-255	1.72E-253	up
AUP1	1.04709939	4.95E-101	5.68E-100	up
AXIN1	1.04732983	2.39E-36	1.01E-35	up
CCDC183	1.0473736	7.66E-08	1.52E-07	up
MAP1LC3A	1.04950116	2.40E-12	5.63E-12	up
SAPCD2	1.05060859	1.67E-78	1.43E-77	up
SNU13	1.05094338	2.40E-123	3.58E-122	up
PSMA7	1.05133856	0	0	up
RPS3	1.05181332	0	0	up
PLIN3	1.05349124	7.97E-291	4.31E-289	up
FAM129B	1.05510164	0	0	up
IRAK1	1.05519457	0	0	up
NAPA	1.05526287	2.19E-68	1.61E-67	up
GIT1	1.05556514	5.43E-116	7.48E-115	up
POLE4	1.05654134	2.36E-33	9.33E-33	up
KLC2	1.0569214	1.05E-34	4.29E-34	up
H1F0	1.05838455	3.05E-151	6.08E-150	up
HSD17B10	1.0585463	2.54E-98	2.81E-97	up
SDF2L1	1.05872128	9.37E-35	3.83E-34	up
OSBPL5	1.05875771	7.08E-10	1.53E-09	up
PRPF3	1.05991477	3.15E-51	1.76E-50	up
SMPD2	1.06063605	7.11E-13	1.70E-12	up
RTL8C	1.06148789	2.55E-55	1.51E-54	up
FP565260.1	1.06168914	8.66E-30	3.17E-29	up
AGFG2	1.06173981	7.38E-16	1.93E-15	up
UPF1	1.06226235	1.88E-163	4.26E-162	up
PTX3	1.06276538	1.91E-20	5.62E-20	up
TPI1	1.06291435	0	0	up
WDR62	1.06348242	3.88E-74	3.14E-73	up
EIF4A3	1.06464097	2.61E-181	7.11E-180	up
ENG	1.06470745	2.65E-96	2.85E-95	up
DCHS1	1.06598928	0.03134813	0.04294707	up
DTX2	1.06598928	1.11E-05	1.98E-05	up
ELFN1	1.06598928	4.60E-05	7.92E-05	up
HRCT1	1.06598928	0.016097	0.0228178	up
KNDC1	1.06598928	0.04940625	0.0658337	up
MINK1	1.06598928	2.41E-71	1.87E-70	up
NANS	1.06598928	1.15E-97	1.26E-96	up
NR4A3	1.06598928	0.03930477	0.05307788	up
PSG1	1.06598928	0.02505694	0.03471224	up
PSG9	1.06598928	8.71E-11	1.94E-10	up
SECTM1	1.06598928	1.36E-05	2.42E-05	up
SLC29A3	1.06598928	0.02006653	0.02812425	up
SOCS3	1.06598928	3.30E-23	1.04E-22	up
SPACA6	1.06598928	0.02006653	0.02812425	up
SSBP4	1.06598928	8.12E-25	2.64E-24	up
CD70	1.06695591	9.50E-65	6.64E-64	up
PLK1	1.06943368	5.73E-303	3.28E-301	up
SSSCA1	1.06963706	2.59E-35	1.07E-34	up
NOCT	1.07066577	5.78E-28	2.03E-27	up
RPS26	1.07069628	1.10E-14	2.79E-14	up
CENPX	1.07304404	7.23E-63	4.91E-62	up

DPP9	1.07490401	2.11E-126	3.23E-125	up
ELOB	1.07525518	4.48E-135	7.55E-134	up
CLK3	1.07528703	1.14E-21	3.46E-21	up
ESRRA	1.07529702	2.54E-28	8.99E-28	up
NFKBID	1.07557533	5.36E-08	1.07E-07	up
ACOT7	1.07609249	5.09E-167	1.22E-165	up
TFE3	1.07668256	9.65E-141	1.72E-139	up
RARA	1.07721654	2.83E-18	7.87E-18	up
RPS14	1.07741663	0	0	up
CIB1	1.07823288	1.77E-47	9.28E-47	up
CDKN2A	1.07916467	1.38E-39	6.19E-39	up
DMAC2	1.07962115	1.20E-56	7.35E-56	up
INF2	1.08032872	4.30E-80	3.74E-79	up
C12orf10	1.08059898	5.83E-36	2.44E-35	up
PLEKHM2	1.08093962	5.03E-127	7.74E-126	up
CCDC12	1.08114909	5.07E-14	1.26E-13	up
SLC41A3	1.08122022	1.03E-38	4.58E-38	up
TMUB1	1.08309654	8.80E-46	4.47E-45	up
TUBG1	1.08395303	1.33E-113	1.78E-112	up
RPS29	1.08409109	1.57E-88	1.52E-87	up
RPS19	1.08467451	0	0	up
NECTIN1	1.08499024	6.30E-25	2.05E-24	up
RGS19	1.08513433	9.31E-25	3.02E-24	up
KRI1	1.08696922	5.96E-47	3.09E-46	up
UBE2J2	1.08702019	7.31E-62	4.88E-61	up
PKM	1.09034602	0	0	up
INTS11	1.0908878	9.82E-94	1.02E-92	up
SLC12A7	1.09108026	1.41E-06	2.64E-06	up
MTG2	1.09110325	6.08E-50	3.33E-49	up
C1QBP	1.09152437	0	0	up
S100A1	1.09152437	0.00071989	0.00114836	up
CXCL1	1.09201207	1.96E-43	9.51E-43	up
ST6GALNAC4	1.09333413	1.52E-55	9.08E-55	up
MRPL36	1.09373427	6.84E-48	3.61E-47	up
ELFN2	1.09421446	1.28E-119	1.83E-118	up
NOL6	1.09504929	1.26E-151	2.54E-150	up
ACD	1.09548495	7.25E-65	5.08E-64	up
NUDT16L1	1.09665642	8.39E-25	2.73E-24	up
G6PD	1.09708752	8.73E-157	1.85E-155	up
BUD31	1.09755936	2.28E-32	8.87E-32	up
CLDN15	1.09769814	1.59E-05	2.81E-05	up
ZFAND2B	1.09816969	8.70E-26	2.88E-25	up
RRP7A	1.09940055	7.45E-87	7.03E-86	up
YARS	1.10017466	0	0	up
TAF6L	1.10148796	2.48E-27	8.54E-27	up
WIZ	1.10152083	3.74E-71	2.90E-70	up
DOT1L	1.10185473	1.34E-41	6.27E-41	up
MLLT1	1.1036115	1.79E-93	1.85E-92	up
OGDH	1.10454252	1.29E-133	2.14E-132	up
ADGRB1	1.10551764	0.00590568	0.00874774	up
SPNS1	1.10551764	4.95E-53	2.84E-52	up
SCARA3	1.10625315	2.27E-06	4.19E-06	up
ARMC6	1.10651316	1.24E-48	6.65E-48	up
9-9月	1.10706901	0	0	up

MEPCE	1.10780946	4.84E-88	4.64E-87	up
RACK1	1.10825473	0	0	up
JUNB	1.10880236	2.98E-09	6.29E-09	up
CDKN2C	1.10886109	1.19E-41	5.58E-41	up
FBXO44	1.10917521	2.79E-35	1.15E-34	up
IMPDH1	1.11140533	7.12E-137	1.22E-135	up
YJU2	1.1120857	5.08E-16	1.33E-15	up
ZNF385A	1.11228293	9.29E-06	1.66E-05	up
MCRIP2	1.11246371	2.38E-17	6.49E-17	up
MAPK8IP2	1.11265734	8.19E-36	3.43E-35	up
JMJD8	1.11291033	6.26E-13	1.50E-12	up
TARBP2	1.11405152	2.73E-36	1.15E-35	up
GNA11	1.11458505	1.69E-116	2.35E-115	up
SHARPIN	1.11510705	2.18E-23	6.89E-23	up
SLC6A9	1.11537731	1.44E-20	4.24E-20	up
HIST1H3E	1.11574232	7.31E-07	1.39E-06	up
GATD1	1.11583783	7.33E-50	4.01E-49	up
ARG2	1.11661535	2.08E-05	3.66E-05	up
VWA1	1.11661535	8.92E-07	1.68E-06	up
TMEM201	1.11690512	2.97E-50	1.64E-49	up
ADGRG1	1.11729051	1.53E-22	4.73E-22	up
POP7	1.11890081	1.16E-34	4.74E-34	up
SOCS1	1.12043706	3.34E-20	9.76E-20	up
SYT14	1.12043706	0.01744047	0.02463729	up
AURKB	1.12063539	1.94E-129	3.07E-128	up
HIP1R	1.12076788	2.05E-32	7.97E-32	up
SSBP1	1.12103064	8.62E-19	2.43E-18	up
MICALL1	1.1221545	1.99E-96	2.15E-95	up
ATXN7L2	1.12232726	2.17E-12	5.11E-12	up
TRABD	1.12341756	3.51E-58	2.19E-57	up
SPHK2	1.12347478	2.20E-13	5.34E-13	up
TCTA	1.12375981	2.75E-26	9.21E-26	up
MBD3	1.12461099	3.19E-103	3.74E-102	up
ABCF3	1.12541037	3.31E-54	1.94E-53	up
MON1A	1.1256522	2.65E-23	8.34E-23	up
NXN	1.12642223	5.05E-124	7.62E-123	up
SLC29A4	1.12653082	2.82E-08	5.69E-08	up
DMPK	1.12782853	7.35E-15	1.87E-14	up
PEA15	1.12837618	1.98E-192	5.82E-191	up
RAB40C	1.13057908	3.34E-28	1.18E-27	up
BANF1	1.13215277	1.76E-142	3.20E-141	up
ZC3H18	1.13232465	2.88E-112	3.82E-111	up
SNTA1	1.13280739	6.58E-45	3.28E-44	up
FKBP8	1.13320019	8.35E-101	9.55E-100	up
GRINA	1.13333275	8.82E-169	2.15E-167	up
RABL6	1.13363244	4.52E-243	1.88E-241	up
MRPL41	1.13365911	1.44E-53	8.30E-53	up
TOM1	1.13373389	4.16E-27	1.42E-26	up
PRMT1	1.13419553	0	0	up
KIF9	1.13541068	1.66E-08	3.38E-08	up
DMWD	1.13614251	2.75E-30	1.02E-29	up
RRP12	1.13644878	9.16E-98	1.01E-96	up
COTL1	1.13716452	0	0	up
FLYWCH2	1.13800053	3.73E-14	9.29E-14	up

S100A11	1.13806218	0	0	up
PRKCD	1.13819066	2.40E-35	9.96E-35	up
PDF	1.13839778	3.00E-08	6.05E-08	up
BIN1	1.13853349	1.08E-67	7.92E-67	up
GIPC1	1.13981769	3.54E-43	1.71E-42	up
OCEL1	1.13998986	3.33E-05	5.78E-05	up
KLF16	1.14029898	5.35E-71	4.12E-70	up
ATF4	1.14043683	0	0	up
PFDN2	1.14127741	4.55E-35	1.87E-34	up
PTGES2	1.14155716	2.08E-88	2.01E-87	up
CORO1B	1.14218277	2.78E-114	3.76E-113	up
SDHAF1	1.14339378	2.68E-14	6.71E-14	up
RBM15B	1.14387795	3.10E-68	2.28E-67	up
FAM181B	1.14399179	0.00413287	0.00620393	up
PBX4	1.14399179	0.00044413	0.00071845	up
CSRNP1	1.14431764	5.62E-25	1.83E-24	up
ATP6V1F	1.145108	6.44E-63	4.38E-62	up
ATG4D	1.14655476	1.64E-16	4.37E-16	up
OTUB1	1.14719856	7.30E-111	9.48E-110	up
SUGP1	1.14806041	2.49E-22	7.65E-22	up
SBF1	1.14852187	2.79E-173	7.04E-172	up
RELB	1.14857251	4.45E-35	1.83E-34	up
ARHGDIB	1.14913052	1.79E-07	3.49E-07	up
DAPK3	1.14919769	2.71E-88	2.61E-87	up
GPR153	1.14964721	2.61E-08	5.28E-08	up
C8orf82	1.14991176	4.32E-21	1.29E-20	up
RNF113A	1.15038147	5.66E-10	1.22E-09	up
SNED1	1.15171915	1.84E-06	3.42E-06	up
CACTIN	1.15452596	5.80E-34	2.33E-33	up
EEF1AKMT1	1.1546469	8.44E-09	1.75E-08	up
NOB1	1.1547595	4.05E-114	5.46E-113	up
NSMF	1.15572193	2.09E-150	4.14E-149	up
ATG2A	1.15599432	1.00E-59	6.44E-59	up
SH2D5	1.15725734	7.53E-27	2.56E-26	up
CENPB	1.15815089	2.32E-177	6.03E-176	up
ZNF837	1.15909868	0.00782591	0.01144319	up
MRPL4	1.16039445	1.46E-98	1.62E-97	up
CST3	1.16048147	6.32E-59	4.00E-58	up
CKB	1.1622046	1.17E-13	2.88E-13	up
MRPS2	1.16271405	6.79E-172	1.70E-170	up
GNB1L	1.16472323	3.85E-14	9.56E-14	up
CNOT3	1.16480702	1.29E-15	3.34E-15	up
JMJD4	1.16487495	4.32E-17	1.16E-16	up
GADD45GIP1	1.16514475	4.23E-28	1.49E-27	up
LAMB3	1.16633981	4.61E-51	2.57E-50	up
KCTD5	1.1691807	8.90E-82	7.93E-81	up
MARK4	1.16941887	4.60E-25	1.50E-24	up
NBL1	1.1727656	4.99E-41	2.31E-40	up
RPS21	1.17293109	0	0	up
PHGDH	1.17310583	4.02E-245	1.69E-243	up
ZNF865	1.17351774	1.66E-19	4.76E-19	up
HAP1	1.17492365	6.21E-07	1.18E-06	up
NOC4L	1.17702059	3.83E-55	2.27E-54	up
TGFBI	1.17720676	2.10E-32	8.16E-32	up

UBTD1	1.17727261	1.00E-35	4.18E-35	up
BFSP1	1.17846401	2.04E-05	3.58E-05	up
COX5B	1.17969585	6.85E-49	3.68E-48	up
GFER	1.18013683	1.20E-37	5.20E-37	up
MAP2K2	1.18093541	1.56E-136	2.67E-135	up
MCOLN1	1.18186999	2.11E-31	8.02E-31	up
FURIN	1.18214551	7.58E-90	7.46E-89	up
TMEM8A	1.18347071	2.12E-109	2.70E-108	up
NME3	1.18348284	1.51E-19	4.32E-19	up
ESS2	1.18382577	3.54E-27	1.21E-26	up
PHLDB3	1.18382577	8.56E-12	1.97E-11	up
CHST7	1.18423825	9.07E-54	5.26E-53	up
SRSF9	1.18542451	1.69E-56	1.03E-55	up
MIDN	1.18614428	7.23E-92	7.32E-91	up
TSTA3	1.1879798	0.00354155	0.00535537	up
PLCD3	1.18860857	9.46E-63	6.42E-62	up
PLK3	1.1887713	2.91E-23	9.15E-23	up
MACROD1	1.18884603	1.87E-11	4.25E-11	up
PROSER2	1.18911366	7.65E-32	2.93E-31	up
NAA10	1.19041473	3.15E-91	3.16E-90	up
EMD	1.19051933	7.62E-81	6.72E-80	up
FBXO2	1.1906587	4.51E-40	2.05E-39	up
CABLES1	1.19374483	0.00021828	0.00036019	up
TUBB6	1.19452046	2.41E-304	1.39E-302	up
KCNJ14	1.1952723	5.56E-05	9.52E-05	up
PREX1	1.1952723	5.56E-05	9.52E-05	up
TPRA1	1.1952723	1.15E-33	4.60E-33	up
SNRNP25	1.19599527	4.91E-27	1.68E-26	up
NDUFA1	1.19801767	1.76E-28	6.25E-28	up
RPS6KA4	1.198318	1.10E-117	1.55E-116	up
ID2	1.19888355	6.69E-08	1.33E-07	up
ERF	1.19909176	4.36E-45	2.18E-44	up
TEAD4	1.19996759	2.07E-68	1.53E-67	up
RAB1B	1.20080057	0	0	up
NDUFAF3	1.20150425	8.70E-39	3.87E-38	up
PKD1	1.2018343	8.30E-73	6.57E-72	up
CYB5R3	1.20225885	1.16E-180	3.12E-179	up
RPL37	1.20233585	0	0	up
SPHK1	1.20245152	7.97E-101	9.13E-100	up
NCLN	1.20335452	5.99E-215	2.08E-213	up
FNDC4	1.2034928	0.00539129	0.00801139	up
NTN1	1.2034928	0.00539129	0.00801139	up
TNFSF13	1.2034928	0.02308388	0.03212547	up
HS6ST1	1.20389754	2.74E-38	1.21E-37	up
INTS5	1.20462626	6.10E-41	2.81E-40	up
PLEKHJ1	1.20489985	6.72E-65	4.71E-64	up
QSOX1	1.20493334	1.00E-155	2.11E-154	up
RPL12	1.20525088	0	0	up
NFKBIA	1.20590559	4.80E-100	5.44E-99	up
GBX2	1.20671431	1.70E-15	4.40E-15	up
FTL	1.20726505	0	0	up
TIMM17B	1.20800829	1.66E-54	9.78E-54	up
LAT	1.21037919	0.00161337	0.00251067	up
OVGP1	1.21037919	0.00161337	0.00251067	up

PPME1	1.21077679	0	0	up
TRAP1	1.21262233	9.58E-130	1.52E-128	up
ADTRP	1.21283067	0.00665636	0.00980387	up
MSX1	1.2137429	2.17E-07	4.23E-07	up
MKNK2	1.21391904	3.91E-193	1.16E-191	up
CEP170B	1.21659472	1.98E-67	1.44E-66	up
THAP3	1.21666819	2.95E-14	7.37E-14	up
TPM2	1.2169438	6.17E-264	2.95E-262	up
AP2S1	1.21731977	3.21E-138	5.58E-137	up
ACBD7	1.21799237	0.02874937	0.03960376	up
ANXA8L1	1.21799237	0.02874937	0.03960376	up
MAP3K8	1.21799237	0.02874937	0.03960376	up
SEMA6B	1.21799237	0.00015196	0.00025358	up
SLC25A18	1.21799237	0.02874937	0.03960376	up
TMEM238	1.21799237	0.00198309	0.00306562	up
ZNF688	1.21799237	0.02874937	0.03960376	up
GATAD2A	1.21858292	4.59E-115	6.25E-114	up
EPHA10	1.22126751	0.00060289	0.00096742	up
MORN1	1.22353056	0.00822185	0.01200975	up
CLPTM1	1.22421281	1.25E-172	3.14E-171	up
SAFB2	1.2250763	7.80E-70	5.87E-69	up
TMEM259	1.22586619	2.18E-214	7.52E-213	up
MAP3K11	1.22617353	1.64E-81	1.46E-80	up
DUSP5	1.2274527	6.88E-114	9.26E-113	up
INO80E	1.22762767	4.22E-13	1.02E-12	up
LRFN4	1.22802274	1.53E-47	8.02E-47	up
FBRS	1.22888069	5.97E-37	2.55E-36	up
ZNF408	1.23068577	2.65E-27	9.12E-27	up
ZMYND19	1.23160864	6.16E-64	4.27E-63	up
PYGB	1.23284754	0	0	up
SIPA1	1.23347606	4.22E-41	1.96E-40	up
LIMD2	1.23358923	3.42E-18	9.48E-18	up
MRPL17	1.23365477	1.00E-35	4.17E-35	up
RUVBL2	1.23380354	9.05E-256	4.06E-254	up
NACC1	1.23537445	5.02E-73	3.98E-72	up
BCL6	1.23591428	0.0101596	0.01473307	up
DNASE1L2	1.23591428	0.0101596	0.01473307	up
FUT2	1.23591428	0.03584504	0.0487543	up
PSG5	1.23591428	0.03584504	0.0487543	up
SLC25A39	1.23671958	4.30E-169	1.05E-167	up
TMEM11	1.23674461	3.25E-48	1.73E-47	up
TECR	1.23750053	4.46E-26	1.49E-25	up
TSPAN4	1.23809655	4.79E-46	2.45E-45	up
STK32C	1.23955286	2.34E-44	1.16E-43	up
GRWD1	1.23968868	8.38E-212	2.82E-210	up
EPHX1	1.24017451	1.79E-65	1.26E-64	up
THOP1	1.24021619	2.55E-132	4.18E-131	up
FXYD5	1.24036969	7.77E-115	1.05E-113	up
BBC3	1.24048701	1.04E-05	1.85E-05	up
RPL11	1.24074569	0	0	up
NOP10	1.24129484	2.42E-70	1.84E-69	up
SOBP	1.24231205	1.29E-07	2.54E-07	up
SFN	1.24485281	0	0	up
SF3B5	1.24495942	5.72E-86	5.34E-85	up

PGF	1.24531298	1.26E-05	2.24E-05	up
CDC20	1.24639395	0	0	up
PLXNA1	1.24684917	1.81E-182	4.99E-181	up
IL1RL1	1.24705484	3.49E-12	8.15E-12	up
RBM14	1.24711249	3.02E-96	3.24E-95	up
UCP2	1.24721596	6.24E-137	1.07E-135	up
CDT1	1.24760805	6.29E-161	1.39E-159	up
SHROOM3	1.24819261	0.00012857	0.00021559	up
CLASRP	1.24853377	3.23E-28	1.14E-27	up
LIPE	1.24958922	3.14E-10	6.85E-10	up
MLST8	1.25019851	5.76E-68	4.22E-67	up
LYPD5	1.25041385	0.01255825	0.01802192	up
TST	1.250907	3.81E-16	1.00E-15	up
CBX4	1.2524024	6.35E-27	2.16E-26	up
TSSK3	1.25361628	0.00136041	0.0021278	up
RPP25	1.25581384	1.24E-13	3.03E-13	up
RIOX1	1.25593218	1.40E-22	4.33E-22	up
METTL27	1.25651119	2.96E-12	6.92E-12	up
ELL	1.25705713	1.44E-37	6.23E-37	up
IBA57	1.25756688	6.72E-23	2.09E-22	up
STRN4	1.25805717	2.61E-158	5.64E-157	up
POR	1.25840044	1.83E-98	2.03E-97	up
MRPS28	1.25863436	0.04473779	0.06000803	up
MYL5	1.25863436	0.04473779	0.06000803	up
NRARP	1.25863436	5.96E-05	0.00010192	up
TMPRSS9	1.25863436	0.04473779	0.06000803	up
CPTP	1.26080546	1.24E-43	6.04E-43	up
CEP131	1.26100526	4.08E-40	1.86E-39	up
TRAPPC12	1.26205442	1.33E-11	3.05E-11	up
DLX2	1.26238649	0.00019151	0.00031741	up
ATAD3A	1.26259632	1.34E-96	1.45E-95	up
FAU	1.26299788	8.99E-208	2.95E-206	up
LSM4	1.26307444	2.49E-122	3.67E-121	up
FAM234A	1.26411642	9.71E-27	3.30E-26	up
UBA52	1.2659036	0	0	up
TIMM44	1.26695032	6.90E-97	7.46E-96	up
C19orf25	1.26700196	3.35E-66	2.40E-65	up
PPL	1.26808176	1.78E-141	3.20E-140	up
SLC25A10	1.26908115	1.19E-12	2.83E-12	up
WTIP	1.27000317	5.37E-22	1.64E-21	up
BICDL1	1.27166431	1.46E-09	3.11E-09	up
TNFSF9	1.27244016	6.26E-32	2.40E-31	up
UQCC2	1.27388213	1.33E-27	4.61E-27	up
ZNF335	1.27438843	2.31E-78	1.98E-77	up
ARF1	1.27656333	0	0	up
SDF4	1.27673199	2.53E-225	9.32E-224	up
IGFBP3	1.27749339	0.00075916	0.00120799	up
EPN1	1.27758346	8.83E-144	1.63E-142	up
RELT	1.27800722	2.40E-43	1.16E-42	up
PDZD4	1.27829288	7.41E-07	1.41E-06	up
WNT5B	1.27952724	6.27E-212	2.12E-210	up
IKBKG	1.27991019	1.21E-17	3.31E-17	up
HGS	1.27999094	9.73E-134	1.63E-132	up
ADM5	1.28011409	0.00010785	0.00018178	up

CYP2S1	1.28100217	4.11E-05	7.08E-05	up
ZNF777	1.28214975	3.11E-32	1.20E-31	up
BCL9L	1.28346111	1.05E-128	1.65E-127	up
RPL18A	1.28523171	1.06E-53	6.14E-53	up
KLHL21	1.28598321	4.17E-104	4.97E-103	up
NCDN	1.28702291	3.25E-82	2.92E-81	up
HELZ2	1.28712005	2.95E-153	5.98E-152	up
HIST1H1D	1.2883817	0.00685229	0.0100808	up
TGFBR3L	1.2883817	0.01919719	0.02698144	up
MAFK	1.28949532	2.21E-26	7.41E-26	up
SLC6A8	1.29007005	1.52E-115	2.09E-114	up
TESK1	1.29015733	3.73E-17	1.01E-16	up
CITED4	1.29090558	1.06E-186	3.01E-185	up
CXorf40B	1.29120222	5.07E-31	1.91E-30	up
TRIM28	1.29219687	0	0	up
GNB2	1.29393623	0	0	up
FAM173A	1.29452084	1.31E-10	2.89E-10	up
DAGLB	1.29459019	2.07E-37	8.94E-37	up
NOTCH1	1.29574753	3.73E-43	1.80E-42	up
SIRPB1	1.2962869	3.77E-12	8.79E-12	up
GPX1	1.29699077	0	0	up
CLPP	1.29760259	4.33E-77	3.66E-76	up
GLI4	1.29947941	1.60E-06	2.98E-06	up
TIMM13	1.30054749	1.51E-54	8.87E-54	up
ZNF628	1.30302848	3.55E-11	8.01E-11	up
CTSD	1.30365889	1.10E-58	6.90E-58	up
MCAT	1.30381174	5.92E-40	2.68E-39	up
CAPN15	1.30416949	1.38E-106	1.70E-105	up
SELENOM	1.30650625	7.59E-33	2.97E-32	up
TEDC1	1.30926043	4.82E-22	1.47E-21	up
MRPS26	1.30952502	2.76E-47	1.44E-46	up
NTMT1	1.31007677	3.62E-29	1.30E-28	up
DOK7	1.31146532	3.36E-10	7.31E-10	up
TMEM115	1.31164161	3.67E-48	1.95E-47	up
NPAS1	1.31391679	0.02372844	0.03295774	up
C19orf48	1.31399483	2.49E-137	4.29E-136	up
C7orf26	1.3158483	2.89E-40	1.32E-39	up
MZT2A	1.31685687	8.53E-20	2.47E-19	up
DVL1	1.31970034	1.15E-145	2.18E-144	up
PSTK	1.31974587	5.15E-07	9.84E-07	up
DDA1	1.31987199	8.16E-54	4.74E-53	up
EEF2	1.32270545	0	0	up
FEM1A	1.32397046	9.86E-63	6.69E-62	up
MYBBP1A	1.32539667	7.23E-252	3.20E-250	up
SLC9A3R1	1.32961873	1.55E-175	3.97E-174	up
PDLIM7	1.33180971	1.47E-113	1.97E-112	up
NOP16	1.33188334	1.59E-86	1.49E-85	up
PAQR4	1.33204293	2.04E-53	1.18E-52	up
EPHA2	1.33263118	0	0	up
ARRDC1	1.33340213	2.36E-49	1.28E-48	up
OR2B6	1.33346959	2.33E-05	4.08E-05	up
PLEKHN1	1.33447812	3.57E-11	8.05E-11	up
ZDHHC12	1.33466079	5.71E-45	2.85E-44	up
MXD4	1.33607844	6.32E-29	2.26E-28	up

NR1D1	1.3372913	1.36E-18	3.82E-18	up
AKR7A2	1.33744533	3.58E-99	4.00E-98	up
CBR3	1.33900777	0.00458328	0.00684941	up
HSD11B2	1.33900777	0.00458328	0.00684941	up
WNK4	1.33900777	0.00458328	0.00684941	up
WNT7B	1.34081098	4.78E-30	1.76E-29	up
GPAT2	1.3460972	0.02930434	0.04025085	up
GPR146	1.3460972	0.02930434	0.04025085	up
NGEF	1.3460972	0.02930434	0.04025085	up
TMEM175	1.3460972	3.15E-17	8.55E-17	up
EGFL7	1.34669471	9.12E-23	2.83E-22	up
ARL2BP	1.34978225	7.38E-05	0.00012536	up
COX6A1	1.34978225	2.07E-08	4.21E-08	up
SCO2	1.34994953	1.08E-47	5.70E-47	up
PIN1	1.3519978	8.97E-75	7.31E-74	up
CASZ1	1.35287043	1.92E-20	5.64E-20	up
RRS1	1.35304692	8.30E-22	2.52E-21	up
TMEM208	1.35347673	5.16E-31	1.94E-30	up
CHST12	1.35397004	1.42E-27	4.92E-27	up
CPA4	1.35423425	2.60E-17	7.08E-17	up
EEFSEC	1.3554959	0.00042364	0.00068574	up
MMP24OS	1.35573697	9.40E-43	4.51E-42	up
ATP13A2	1.35603664	8.60E-148	1.67E-146	up
FUS	1.35662609	0	0	up
PQBP1	1.35713439	1.99E-110	2.58E-109	up
DDX54	1.35745209	3.76E-123	5.58E-122	up
ACTA2	1.35934822	2.61E-09	5.52E-09	up
YBX1	1.35940137	0	0	up
CCDC86	1.36073774	4.97E-231	1.91E-229	up
PECAM1	1.36144516	0.00559718	0.00830814	up
ARMC5	1.36325532	8.79E-29	3.14E-28	up
NOSIP	1.36342479	5.00E-154	1.02E-152	up
RPS19BP1	1.36439802	1.10E-73	8.79E-73	up
PCSK1N	1.36554956	0.00250223	0.00382963	up
AGPAT2	1.36803551	1.52E-71	1.18E-70	up
WDR18	1.36928756	1.42E-83	1.29E-82	up
CASTOR2	1.36977003	1.93E-06	3.57E-06	up
LMF2	1.37128137	8.32E-172	2.08E-170	up
TXNRD2	1.37203127	1.28E-55	7.63E-55	up
SYT7	1.3730441	8.96E-14	2.20E-13	up
KREMEN2	1.37453835	9.43E-12	2.17E-11	up
SYNPO	1.37548439	4.35E-49	2.35E-48	up
C19orf70	1.37617217	7.15E-25	2.33E-24	up
SMARCA4	1.3763294	5.92E-37	2.53E-36	up
GUK1	1.37696121	6.12E-163	1.38E-161	up
TSC22D4	1.37743453	5.13E-71	3.96E-70	up
ZNF76	1.37748192	1.48E-36	6.27E-36	up
RPL27	1.37900553	0	0	up
RRP9	1.37910056	2.62E-120	3.78E-119	up
TOR4A	1.38021901	6.40E-185	1.79E-183	up
GET4	1.38137453	1.66E-71	1.29E-70	up
RNASEH2C	1.38384771	2.77E-69	2.07E-68	up
CNN2	1.38385955	0	0	up
GADD45B	1.38508578	3.60E-34	1.45E-33	up

FOSL1	1.38556962	5.75E-236	2.29E-234	up
BAMBI	1.38791738	0.03612487	0.04912443	up
CCNK	1.38791738	1.73E-12	4.09E-12	up
ENTPD8	1.38791738	0.03612487	0.04912443	up
MXRA8	1.38791738	0.0030415	0.00462361	up
NXPH4	1.38791738	1.46E-09	3.10E-09	up
ST3GAL3	1.38791738	0.0030415	0.00462361	up
MAP7D1	1.38827891	3.29E-256	1.48E-254	up
TAF4	1.39022384	9.29E-22	2.82E-21	up
BRI3	1.39058163	1.62E-88	1.56E-87	up
NUDT1	1.39088894	2.96E-17	8.03E-17	up
E4F1	1.3927996	2.73E-49	1.48E-48	up
TAGLN2	1.39411525	0	0	up
PROB1	1.39431515	3.28E-16	8.66E-16	up
PLPPR2	1.39461203	1.52E-29	5.54E-29	up
PDAP1	1.39587343	3.69E-142	6.66E-141	up
NATD1	1.39756755	6.09E-26	2.03E-25	up
CYC1	1.398261	0	0	up
SNX8	1.39871891	6.83E-136	1.16E-134	up
C1QL4	1.39941301	1.55E-05	2.74E-05	up
LGALS1	1.399459	0	0	up
ATG101	1.39952867	1.58E-98	1.75E-97	up
HIST1H2BH	1.40159231	8.69E-26	2.88E-25	up
FBXW5	1.40166899	1.22E-233	4.76E-232	up
SMOX	1.40187291	8.24E-113	1.09E-111	up
RBM42	1.40213881	5.72E-124	8.60E-123	up
RALY	1.40285666	0	0	up
RPS5	1.40312901	0	0	up
SLC9A3R2	1.40343037	4.96E-26	1.65E-25	up
STK11	1.4040011	3.96E-148	7.71E-147	up
BRMS1	1.4041276	5.23E-118	7.37E-117	up
SGTA	1.40437977	0	0	up
CCDC97	1.40589245	5.35E-57	3.28E-56	up
BCL7C	1.40596383	2.59E-25	8.50E-25	up
D2HGDH	1.40802495	1.42E-20	4.19E-20	up
ADM	1.40828517	1.28E-71	9.96E-71	up
SLC9A1	1.40831572	5.23E-104	6.21E-103	up
BAIAP2	1.40843538	3.39E-64	2.36E-63	up
MAP3K14	1.40974174	2.57E-145	4.85E-144	up
ALG3	1.41070474	1.35E-68	9.96E-68	up
TMEM53	1.41081778	6.68E-10	1.44E-09	up
PIAS4	1.41112477	5.03E-51	2.80E-50	up
HIST1H4I	1.41141823	1.47E-149	2.88E-148	up
NDRG1	1.41142908	1.55E-60	1.01E-59	up
NACA2	1.41176412	1.86E-05	3.28E-05	up
OSR2	1.41176412	1.86E-05	3.28E-05	up
XAB2	1.41372407	1.17E-138	2.04E-137	up
RPS11	1.41641096	0	0	up
COX6B1	1.41732416	6.57E-121	9.54E-120	up
MCRIP1	1.42002956	2.45E-54	1.44E-53	up
RAD23A	1.4211647	2.35E-29	8.52E-29	up
MAP1S	1.42178758	5.05E-46	2.58E-45	up
ASPHD1	1.42507037	2.24E-05	3.92E-05	up
SAC3D1	1.42516442	4.08E-46	2.09E-45	up

AOC2	1.42855936	2.70E-06	4.97E-06	up
DISP3	1.42855936	4.83E-05	8.30E-05	up
PKMYT1	1.43159577	3.93E-88	3.78E-87	up
HIST1H2AC	1.43277161	0	0	up
PEAR1	1.43319825	1.40E-13	3.43E-13	up
PTRH1	1.43336035	3.04E-10	6.64E-10	up
NDUFB10	1.43668474	7.62E-96	8.12E-95	up
CLUH	1.43740615	0	0	up
TIMM8B	1.4374994	1.17E-26	3.96E-26	up
HYAL2	1.43830907	3.18E-83	2.88E-82	up
MT2A	1.43978435	0	0	up
IER5	1.43980412	4.93E-39	2.20E-38	up
MAFF	1.44020788	1.45E-42	6.93E-42	up
CFL1	1.44028237	0	0	up
RPS6KB2	1.44080642	2.79E-70	2.12E-69	up
TEX264	1.4412156	5.30E-67	3.83E-66	up
FAM171A2	1.44135663	2.65E-20	7.77E-20	up
LLGL2	1.44281206	5.01E-48	2.66E-47	up
EEF1A2	1.44306771	0	0	up
CITED2	1.44328087	8.05E-82	7.17E-81	up
CYTH4	1.4445009	0.04437367	0.05956034	up
GALNT9	1.4445009	0.04437367	0.05956034	up
GALR2	1.4445009	0.00446435	0.00668186	up
SLC12A5	1.4445009	0.000497	0.00080154	up
RPL18	1.44458408	0	0	up
NDUFA2	1.44586472	6.52E-45	3.25E-44	up
MYDGF	1.44907944	3.11E-109	3.93E-108	up
ATP2A1	1.44931792	1.49E-05	2.64E-05	up
HCN2	1.4504872	9.85E-35	4.03E-34	up
JPH2	1.45065313	5.84E-08	1.16E-07	up
SSBP3	1.45101421	7.65E-17	2.05E-16	up
SELENOO	1.45108238	8.53E-53	4.88E-52	up
FNDC11	1.4530124	0.00109147	0.00171992	up
P2RY11	1.4530124	0.00109147	0.00171992	up
SH3BGRL3	1.45443257	1.56E-119	2.22E-118	up
SNAPC4	1.4564881	8.02E-70	6.03E-69	up
CAMSAP3	1.45677923	4.64E-26	1.55E-25	up
MED16	1.45719941	2.40E-169	5.87E-168	up
B3GNT4	1.4583067	0.00027302	0.00044769	up
RHOF	1.4583067	0.00027302	0.00044769	up
TFAP4	1.45990349	2.30E-09	4.87E-09	up
SIRT7	1.46269496	7.98E-56	4.79E-55	up
MRPS34	1.4632312	1.06E-105	1.29E-104	up
SLC35E4	1.46340111	1.20E-33	4.76E-33	up
HIST1H3A	1.46453866	0.00241332	0.0037011	up
TNFRSF12A	1.46643206	7.96E-170	1.95E-168	up
ERFE	1.46808772	2.74E-09	5.78E-09	up
ZNF414	1.47037954	5.18E-11	1.16E-10	up
SPINDOC	1.47114527	7.14E-56	4.29E-55	up
ZNF428	1.47140236	4.60E-19	1.31E-18	up
PSMD3	1.47290198	0	0	up
POLR2E	1.47702855	0	0	up
U2AF2	1.4775027	0	0	up
BICRA	1.47771405	4.52E-22	1.38E-21	up

LRCH4	1.47851992	3.69E-42	1.74E-41	up
NDUFS7	1.47967277	1.51E-101	1.73E-100	up
ATP5F1D	1.47973808	3.29E-211	1.10E-209	up
AMH	1.48102678	6.89E-09	1.43E-08	up
ANKRD16	1.48102678	4.86E-10	1.05E-09	up
AQP3	1.48102678	0.02305815	0.03209841	up
FIBCD1	1.48102678	5.49E-06	9.96E-06	up
GP1BA	1.48102678	0.00538262	0.00799991	up
NUDT8	1.48102678	2.12E-05	3.73E-05	up
PANX2	1.48102678	0.02305815	0.03209841	up
PPM1J	1.48102678	0.02305815	0.03209841	up
TMEM255B	1.48102678	0.02305815	0.03209841	up
MRPL55	1.48193271	3.40E-39	1.52E-38	up
KRT15	1.48245732	6.65E-73	5.27E-72	up
PPP1R35	1.48521458	5.92E-18	1.64E-17	up
TGFB1	1.48872939	1.09E-267	5.24E-266	up
EIF3G	1.48934205	2.16E-133	3.59E-132	up
RPS16	1.4897211	0	0	up
GAS2L1	1.49122255	1.51E-82	1.36E-81	up
MB	1.49225404	0.00017943	0.00029801	up
ARHGAP23	1.49249197	9.62E-29	3.44E-28	up
CAVIN1	1.4932491	0	0	up
DPM3	1.49483258	4.58E-09	9.58E-09	up
C19orf71	1.49483258	1.30E-11	2.98E-11	up
UBE2M	1.49483258	3.54E-89	3.45E-88	up
C6orf226	1.49662363	6.54E-06	1.18E-05	up
DDRGK1	1.49698907	1.29E-38	5.73E-38	up
FAM43A	1.49854688	1.81E-27	6.28E-27	up
OGFR	1.49858515	9.52E-144	1.75E-142	up
MDGA1	1.49894869	0.00289679	0.0044123	up
MRPL28	1.49955404	2.24E-138	3.90E-137	up
ASB16	1.5003921	9.68E-09	2.00E-08	up
AC010422.3	1.50064971	0	0	up
NDUFS6	1.50159699	3.32E-76	2.77E-75	up
PLEC	1.50346679	0	0	up
L1CAM	1.50385555	1.59E-67	1.16E-66	up
PIEZO1	1.50523791	0	0	up
HIST2H2BE	1.51087259	8.33E-61	5.45E-60	up
RNF126	1.51401126	5.30E-171	1.32E-169	up
SLC1A5	1.51537621	0	0	up
POLR2I	1.51537828	2.61E-51	1.46E-50	up
NOC2L	1.51740145	0	0	up
TRAPPC2L	1.51916191	3.91E-136	6.65E-135	up
UBALD1	1.52040631	5.02E-28	1.76E-27	up
KRT18	1.52090553	0	0	up
RNF40	1.52508543	1.24E-159	2.71E-158	up
DLC1	1.5254209	0.00011731	0.00019729	up
KRT32	1.5254209	0.00011731	0.00019729	up
PLAUR	1.52585656	1.07E-62	7.22E-62	up
TBC1D2	1.52593114	1.24E-157	2.65E-156	up
LRWD1	1.52650441	8.79E-100	9.92E-99	up
EIF3C	1.53006427	1.70E-30	6.32E-30	up
PPP1R12C	1.53019357	2.83E-130	4.51E-129	up
SERINC2	1.53413812	3.54E-20	1.03E-19	up

TREX2	1.53547456	0.0004638	0.00074904	up
TRAF1	1.53573819	4.89E-33	1.92E-32	up
CDC34	1.53614325	1.32E-149	2.58E-148	up
CRK	1.537295	1.12E-10	2.47E-10	up
MMP15	1.53936009	4.80E-23	1.50E-22	up
HIC1	1.53992047	4.11E-07	7.89E-07	up
RPUSD1	1.54339156	4.27E-133	7.07E-132	up
ADIRF	1.54403658	0.00025353	0.00041678	up
RASSF7	1.54403658	2.53E-13	6.13E-13	up
HPCAL1	1.54723236	0	0	up
SSNA1	1.54735154	1.49E-97	1.63E-96	up
ROGDI	1.55037483	1.37E-22	4.25E-22	up
DENND6B	1.55141611	0.02782577	0.03840197	up
INHBB	1.55473647	3.97E-08	7.97E-08	up
MAP2K3	1.55555354	2.12E-165	4.92E-164	up
C7orf50	1.55772663	7.38E-61	4.83E-60	up
RHOC	1.55870302	2.57E-200	8.00E-199	up
CCDC137	1.55942369	4.34E-84	3.96E-83	up
MTLN	1.56075397	8.07E-16	2.11E-15	up
KRT8	1.56131854	0	0	up
DDX49	1.56664928	1.15E-71	8.97E-71	up
ACHE	1.56848962	0.01454857	0.02071036	up
H3F3C	1.56848962	0.01454857	0.02071036	up
ZDHHC11	1.56848962	0.01454857	0.02071036	up
NMB	1.57078143	1.21E-09	2.58E-09	up
DNLZ	1.57364606	6.46E-27	2.20E-26	up
PEX16	1.5746682	7.29E-53	4.17E-52	up
GPRC5C	1.57482608	2.00E-98	2.22E-97	up
MEA1	1.57500293	7.59E-39	3.38E-38	up
HOMER3	1.5767272	1.34E-26	4.52E-26	up
PQLC1	1.57715696	5.41E-137	9.26E-136	up
CARMIL2	1.57819544	4.08E-15	1.05E-14	up
SNRNP70	1.57871166	0	0	up
STC2	1.5788787	1.56E-143	2.86E-142	up
ARL4D	1.58056245	0.0001639	0.00027279	up
RCOR2	1.58056245	0.00769688	0.01125968	up
C15orf39	1.58233517	7.83E-29	2.80E-28	up
ARID5A	1.58326497	1.73E-19	4.95E-19	up
CORO1A	1.5833885	5.59E-13	1.34E-12	up
AKT1S1	1.584177	1.11E-196	3.35E-195	up
LAGE3	1.58482219	3.13E-43	1.52E-42	up
APRT	1.58766762	1.38E-232	5.33E-231	up
CLIC3	1.58955124	0.00410776	0.0061695	up
MISP	1.58955124	1.39E-10	3.07E-10	up
NNAT	1.58955124	0.00410776	0.0061695	up
CDC37	1.59106394	0	0	up
CARD9	1.596504	0.00220713	0.00339796	up
SART1	1.5987352	6.18E-115	8.41E-114	up
TRIR	1.60027489	5.73E-79	4.95E-78	up
CD320	1.60199188	6.91E-228	2.60E-226	up
ABTB2	1.60204218	2.31E-93	2.38E-92	up
B3GALT6	1.60204218	3.51E-52	1.99E-51	up
MMP17	1.60285749	9.67E-30	3.54E-29	up
SRM	1.60490441	0	0	up

EHD1	1.60549176	0	0	up
CCND3	1.60790731	1.39E-140	2.47E-139	up
RAB19	1.60851651	7.80E-07	1.48E-06	up
DEDD2	1.60904483	1.55E-39	6.96E-39	up
FNDC10	1.6103098	4.33E-07	8.31E-07	up
NAA38	1.6103098	2.10E-32	8.17E-32	up
TMEM132A	1.61124207	6.61E-207	2.15E-205	up
CHERP	1.6126633	5.22E-67	3.77E-66	up
PODXL	1.61347708	8.82E-14	2.17E-13	up
RABGEF1	1.61347708	0.00019244	0.00031891	up
TUBB4B	1.61356608	0	0	up
HMOX1	1.61543948	0	0	up
ROMO1	1.61618636	7.43E-38	3.23E-37	up
MAD1L1	1.61644893	1.36E-64	9.53E-64	up
HCFC1R1	1.62057813	5.79E-13	1.39E-12	up
ADRM1	1.62090868	0	0	up
SMIM29	1.62340545	3.48E-35	1.44E-34	up
FGFR1	1.62419905	3.90E-92	3.97E-91	up
AKAP8L	1.62870714	1.20E-74	9.72E-74	up
HIST1H2BD	1.63061389	4.73E-93	4.85E-92	up
CTXN1	1.63771595	2.13E-19	6.09E-19	up
AJM1	1.63972453	4.41E-12	1.03E-11	up
BOP1	1.64088468	0	0	up
FARSA	1.64280094	1.80E-88	1.74E-87	up
PUSL1	1.64845793	2.36E-49	1.28E-48	up
AC005726.1	1.65095178	0.03315848	0.04531042	up
CDC42	1.65095178	0.03315848	0.04531042	up
DUSP2	1.65095178	0.00139715	0.00218419	up
GAL	1.65095178	0.01723805	0.02436485	up
HIST1H2AI	1.65095178	9.71E-09	2.00E-08	up
LRP11	1.65095178	3.71E-05	6.41E-05	up
PET117	1.65095178	0.01723805	0.02436485	up
RTN4R	1.65095178	5.86E-31	2.20E-30	up
STARD5	1.65095178	0.03315848	0.04531042	up
TMEM151A	1.65095178	8.95E-14	2.20E-13	up
UCN	1.65095178	0.01723805	0.02436485	up
ZNF469	1.65095178	5.86E-07	1.12E-06	up
POLG	1.65165571	1.03E-170	2.56E-169	up
HMGA1	1.6519525	0	0	up
APBA3	1.65495371	1.22E-31	4.65E-31	up
SMTN	1.6559266	0	0	up
NDUFB7	1.65790454	5.83E-19	1.65E-18	up
AURKAIP1	1.65961664	4.62E-168	1.12E-166	up
TSSC4	1.659669	3.64E-43	1.76E-42	up
STX1A	1.66008279	4.19E-28	1.48E-27	up
UPP1	1.66119581	4.43E-213	1.50E-211	up
PRELID1	1.66422701	2.08E-156	4.40E-155	up
SCAF1	1.66832303	2.85E-204	9.10E-203	up
STUB1	1.67289433	2.51E-46	1.29E-45	up
THAP7	1.67588018	3.97E-41	1.84E-40	up
PAK4	1.67676427	8.97E-135	1.51E-133	up
TUBB3	1.67742399	3.98E-06	7.27E-06	up
ALYREF	1.67769439	1.71E-226	6.39E-225	up
IMP4	1.67823586	2.83E-168	6.88E-167	up

RHOD	1.67963454	3.37E-23	1.06E-22	up
REXO1	1.68075179	1.92E-146	3.68E-145	up
TOMM40	1.68093094	0	0	up
SSTR5	1.68489911	4.29E-05	7.39E-05	up
DGKZ	1.68597691	8.61E-140	1.52E-138	up
ANXA8	1.68614121	1.29E-08	2.65E-08	up
DUS3L	1.68693108	1.41E-53	8.16E-53	up
RPL13	1.69177721	0	0	up
ITPKA	1.69277196	1.42E-10	3.13E-10	up
PDLIM2	1.69326259	6.05E-61	3.97E-60	up
ADPRHL1	1.6940205	0.00026043	0.00042775	up
MEX3D	1.694607	1.52E-116	2.10E-115	up
PFN1	1.69540851	0	0	up
DNAJC30	1.69634368	4.34E-21	1.29E-20	up
CBARP	1.69696418	7.69E-21	2.28E-20	up
AHDC1	1.69734469	5.31E-56	3.20E-55	up
LRP3	1.6982575	0.00047744	0.00077038	up
POLR2F	1.69913531	2.16E-72	1.70E-71	up
SPSB1	1.6993148	3.37E-25	1.10E-24	up
KRT86	1.69986138	2.59E-09	5.47E-09	up
MPG	1.70077847	1.94E-90	1.92E-89	up
HES6	1.70271321	6.68E-41	3.08E-40	up
FABP5	1.70425001	2.38E-18	6.64E-18	up
FTH1	1.71170581	2.42E-178	6.33E-177	up
MFSD12	1.71655	3.45E-297	1.91E-295	up
PLEKHF1	1.72740384	2.68E-15	6.91E-15	up
FAM53A	1.72895429	0.0056169	0.00833354	up
ISG20	1.72895429	0.0056169	0.00833354	up
LYRM9	1.72895429	0.0056169	0.00833354	up
ZDHHC22	1.72895429	8.99E-05	0.00015214	up
SLC16A3	1.73130733	0	0	up
C11orf68	1.73187178	1.43E-74	1.16E-73	up
C10orf55	1.73341394	2.91E-06	5.34E-06	up
RPL41	1.74496409	0	0	up
PDZK1IP1	1.7487991	5.61E-40	2.54E-39	up
MNX1	1.75147866	9.96E-30	3.64E-29	up
H2AFX	1.75267036	0	0	up
CDC42EP1	1.75294056	1.31E-261	6.09E-260	up
ZGPAT	1.75404527	1.02E-06	1.92E-06	up
S100A3	1.75506018	7.03E-16	1.84E-15	up
RIN1	1.75644491	6.64E-44	3.26E-43	up
CSKMT	1.75786699	3.85E-09	8.07E-09	up
SERTAD1	1.75822852	4.01E-57	2.46E-56	up
SH3GL1	1.75842565	0	0	up
IL32	1.75901153	2.40E-10	5.26E-10	up
HIST2H2BF	1.7611347	1.11E-07	2.20E-07	up
SLC3A2	1.76205472	0	0	up
ABHD17A	1.76387822	2.67E-142	4.83E-141	up
CCDC96	1.766429	0.00100773	0.00159189	up
PELI2	1.766429	0.0200601	0.02811987	up
SLC45A1	1.766429	0.00100773	0.00159189	up
RPS2	1.76760105	0	0	up
GDF15	1.77613847	0	0	up
JOSD2	1.77736316	5.08E-29	1.82E-28	up

HIST1H3H	1.78105018	9.53E-68	6.97E-67	up
SPNS2	1.78219631	4.22E-103	4.94E-102	up
METRN	1.78310774	1.20E-105	1.45E-104	up
BTBD2	1.78403663	1.92E-166	4.55E-165	up
EIF6	1.78722904	3.49E-164	8.01E-163	up
CLTB	1.78796869	7.07E-193	2.08E-191	up
ZNF787	1.78933527	3.22E-47	1.68E-46	up
TMEM158	1.79638222	3.18E-15	8.18E-15	up
NRG2	1.79805608	6.24E-20	1.81E-19	up
FKRP	1.7987054	7.97E-44	3.91E-43	up
ZNHIT2	1.80097723	2.23E-24	7.19E-24	up
DNAH7	1.80295487	0.03860333	0.05217485	up
KLF17	1.80295487	0.00344294	0.00521121	up
RUNDC3A	1.80295487	0.03860333	0.05217485	up
TEX14	1.80295487	0.03860333	0.05217485	up
FAM110A	1.81684932	2.80E-31	1.06E-30	up
FGFR3	1.82498118	6.13E-10	1.32E-09	up
PPAN	1.82498118	1.21E-05	2.16E-05	up
NME2	1.83152403	0.00640962	0.00945629	up
WWC3	1.83152403	0.00640962	0.00945629	up
RAVER1	1.8347582	9.58E-164	2.19E-162	up
DOHH	1.83677975	3.09E-91	3.10E-90	up
HES4	1.83921842	9.77E-20	2.82E-19	up
INKA1	1.84359686	0.00113963	0.00179281	up
BCAR1	1.84805776	6.48E-51	3.61E-50	up
AKNAD1	1.85026059	0.00020988	0.0003469	up
GJD3	1.85026059	0.00020988	0.0003469	up
SOX18	1.85026059	0.00020988	0.0003469	up
CIAO2B	1.85160404	3.76E-94	3.94E-93	up
C9orf16	1.8519993	3.30E-73	2.63E-72	up
NAT8L	1.85215887	4.68E-18	1.29E-17	up
ELOF1	1.85448518	6.14E-35	2.52E-34	up
CEBPB	1.85686599	3.09E-162	6.93E-161	up
MYPN	1.85740266	7.54E-06	1.36E-05	up
C6orf132	1.86012858	1.46E-17	3.98E-17	up
ZNF598	1.86832496	3.30E-212	1.12E-210	up
LY6K	1.86981251	0	0	up
HSPA12B	1.8733442	0.00038144	0.00061923	up
C1QTNF1	1.8733442	0.00209343	0.00322896	up
HIST2H2AC	1.8733442	0.00209343	0.00322896	up
RAB5IF	1.8733442	1.34E-19	3.84E-19	up
TPRN	1.87776017	1.97E-82	1.77E-81	up
TBX2	1.87890373	1.72E-14	4.31E-14	up
LIPC	1.88167548	9.06E-105	1.09E-103	up
SLC7A5	1.88294962	0	0	up
C19orf53	1.88399145	6.08E-49	3.27E-48	up
ZFPM1	1.88701914	4.61E-28	1.62E-27	up
PLXNA2	1.88911152	4.67E-06	8.48E-06	up
FKBP2	1.89450881	1.31E-32	5.12E-32	up
RPL28	1.90198122	0	0	up
HYPK	1.90249055	9.69E-17	2.59E-16	up
FSCN1	1.9093281	0	0	up
LYL1	1.91398619	0.00385495	0.00580381	up
NHP2	1.91988679	1.36E-46	7.00E-46	up

BAIAP3	1.9310597	9.92E-07	1.87E-06	up
PNPLA2	1.93811446	2.80E-170	6.90E-169	up
C8G	1.9404584	0.02261849	0.03153285	up
FRMPD3	1.9404584	0.02261849	0.03153285	up
LRRC73	1.9404584	0.02261849	0.03153285	up
PDE2A	1.9404584	0.02261849	0.03153285	up
PROC	1.9404584	0.02261849	0.03153285	up
WNT9A	1.94569505	3.97E-11	8.94E-11	up
RPL35	1.94685932	0	0	up
DRAP1	1.95493174	2.09E-213	7.17E-212	up
RPS28	1.95528582	1.14E-18	3.20E-18	up
CAMK2N2	1.95907408	0.0004199	0.00067995	up
FSTL3	1.96110271	1.20E-33	4.79E-33	up
TRMT61A	1.96341955	5.29E-121	7.72E-120	up
PPP1R14B	1.96788414	2.87E-213	9.83E-212	up
CHAC1	1.97177394	4.33E-36	1.82E-35	up
PRR7	1.97198072	5.32E-44	2.62E-43	up
DACT3	1.97287988	0.00711164	0.01044668	up
ARHGDIA	1.97746613	0	0	up
EIF4EBP1	1.97870486	1.72E-152	3.47E-151	up
CNTNAP3C	1.98025941	1.08E-06	2.03E-06	up
EPHB2	1.9826609	2.46E-21	7.40E-21	up
YDJC	1.98309627	2.21E-159	4.81E-158	up
UBE2S	1.98644764	0	0	up
MMP25	1.9919887	0.00229992	0.00353549	up
S100A5	1.9919887	0.00229992	0.00353549	up
SEMA7A	2.00458874	1.91E-06	3.54E-06	up
PIM3	2.01435651	5.52E-162	1.23E-160	up
H1FX	2.01990351	5.81E-279	2.97E-277	up
SVEP1	2.0294634	9.83E-06	1.76E-05	up
PPP1R15A	2.03599454	3.36E-106	4.10E-105	up
UQCC3	2.05281389	6.29E-26	2.09E-25	up
ABHD16B	2.05916801	3.06E-25	1.00E-24	up
AC233723.1	2.06598928	0.04281934	0.05758315	up
AP3B2	2.06598928	0.04281934	0.05758315	up
CGREF1	2.06598928	0.00045113	0.0007294	up
CHRNA10	2.06598928	0.01311314	0.01877703	up
CNFN	2.06598928	5.92E-06	1.07E-05	up
CRLF1	2.06598928	2.03E-06	3.76E-06	up
HIST1H2AL	2.06598928	0.00417741	0.00626858	up
IQCJ-SCHIP1	2.06598928	2.03E-06	3.76E-06	up
LRRC66	2.06598928	0.04281934	0.05758315	up
NPIPB2	2.06598928	5.10E-05	8.76E-05	up
PWP2	2.06598928	0.00015107	0.00025212	up
SLC17A3	2.06598928	0.04281934	0.05758315	up
SRRM5	2.06598928	0.04281934	0.05758315	up
TNFRSF17	2.06598928	0.01311314	0.01877703	up
VPS28	2.06598928	0.04281934	0.05758315	up
IGFBP4	2.06789282	0	0	up
TMSB10	2.07564226	0	0	up
FOSB	2.07796192	2.65E-15	6.82E-15	up
COX8A	2.08093962	1.55E-56	9.43E-56	up
MIB2	2.08279757	4.92E-31	1.85E-30	up
NMNAT2	2.08421304	8.64E-29	3.09E-28	up

EXOSC4	2.09116784	3.28E-100	3.73E-99	up
NLRP1	2.09152437	1.30E-14	3.27E-14	up
RAB20	2.09347002	1.46E-07	2.86E-07	up
SLC37A2	2.10161319	3.56E-06	6.51E-06	up
EDF1	2.10249894	3.88E-262	1.82E-260	up
AC068631.2	2.10551764	1.04E-05	1.86E-05	up
CD68	2.10551764	6.28E-23	1.96E-22	up
SAMD11	2.11661535	8.99E-05	0.00015217	up
CEBPA	2.12488297	2.53E-07	4.92E-07	up
FZD8	2.12488297	2.53E-07	4.92E-07	up
KRT16	2.12488297	2.53E-07	4.92E-07	up
GLIS2	2.13011962	7.33E-07	1.39E-06	up
UNC5A	2.13207847	2.23E-09	4.72E-09	up
SF3A2	2.13930596	1.56E-105	1.88E-104	up
LSM7	2.14257748	1.25E-94	1.32E-93	up
HIST1H2BF	2.14399179	1.64E-10	3.61E-10	up
CYBA	2.14452642	0	0	up
GPR55	2.15345212	0.00243498	0.00373297	up
SERF2	2.1621417	2.46E-282	1.28E-280	up
PCDH1	2.16552495	5.32E-05	9.13E-05	up
HIST1H2BC	2.17583646	1.66E-42	7.89E-42	up
C11orf94	2.1814665	0.00753376	0.01102865	up
ETV2	2.1814665	0.00753376	0.01102865	up
KLHL30	2.1814665	0.00753376	0.01102865	up
THAP8	2.1814665	2.30E-09	4.86E-09	up
ANKRD1	2.18275635	1.65E-46	8.53E-46	up
C19orf24	2.18723498	3.34E-152	6.74E-151	up
GTF2IRD2	2.2034928	0.00046866	0.00075669	up
CCDC85B	2.20509136	1.17E-220	4.16E-219	up
VGF	2.20894723	5.40E-08	1.08E-07	up
UBALD2	2.23306592	2.48E-95	2.64E-94	up
BATF2	2.23591428	0.02397269	0.03326801	up
C19orf73	2.23591428	0.02397269	0.03326801	up
C1QTNF12	2.23591428	0.02397269	0.03326801	up
C3orf80	2.23591428	0.00140958	0.00220214	up
CLDN14	2.23591428	0.02397269	0.03326801	up
ECM1	2.23591428	0.02397269	0.03326801	up
FAM229A	2.23591428	0.02397269	0.03326801	up
GP1BB	2.23591428	0.02397269	0.03326801	up
KRT81	2.23591428	0.02397269	0.03326801	up
MSRA	2.23591428	0.02397269	0.03326801	up
SBK3	2.23591428	0.00140958	0.00220214	up
SLC5A2	2.23591428	0.02397269	0.03326801	up
ST6GALNAC6	2.23591428	0.00140958	0.00220214	up
TNFRSF19	2.23591428	0.00140958	0.00220214	up
WNT4	2.23591428	0.02397269	0.03326801	up
HIST1H3G	2.2399498	3.00E-68	2.21E-67	up
S100A2	2.24740992	3.04E-72	2.39E-71	up
ZNF296	2.25693206	5.34E-32	2.05E-31	up
CCDC124	2.25893093	1.15E-88	1.12E-87	up
CPLX1	2.26762314	2.63E-07	5.11E-07	up
CTU1	2.26973595	2.31E-53	1.33E-52	up
MMP1	2.27244016	3.94E-09	8.26E-09	up
BTBD19	2.2883817	0.0042946	0.00643534	up

CEND1	2.2883817	5.38E-05	9.22E-05	up
SNAI3	2.2883817	0.0042946	0.00643534	up
TNFAIP8L3	2.2883817	0.0042946	0.00643534	up
C4orf48	2.29764327	6.40E-75	5.24E-74	up
POLR2L	2.30242948	0	0	up
RASL11A	2.30302848	1.08E-05	1.92E-05	up
LPCAT1	2.30926043	1.46E-31	5.55E-31	up
C19orf57	2.31391679	0.00081096	0.00128849	up
PSMB10	2.31391679	2.11E-11	4.81E-11	up
ADAT3	2.32182819	3.74E-43	1.81E-42	up
RPL8	2.3260717	0	0	up
NANOS1	2.32902369	0.00015768	0.00026284	up
ANGPTL4	2.33502243	1.63E-64	1.13E-63	up
HIST1H4H	2.35412906	2.86E-140	5.06E-139	up
SH2B2	2.35877103	5.33E-08	1.06E-07	up
TPGS1	2.36416877	1.33E-33	5.30E-33	up
KLF2	2.36764498	8.26E-30	3.03E-29	up
MRPL12	2.37084386	9.05E-13	2.16E-12	up
TUBB2A	2.37098698	2.37E-80	2.06E-79	up
ACTL10	2.38791738	0.01330897	0.019019	up
CASKIN1	2.38791738	0.00243161	0.00372825	up
GNA15	2.38791738	0.01330897	0.019019	up
IGFBP1	2.38791738	2.10E-18	5.86E-18	up
MILR1	2.38791738	0.00046398	0.00074923	up
RCSD1	2.38791738	0.01330897	0.019019	up
SEMA3G	2.38791738	0.01330897	0.019019	up
SPINT1	2.38791738	0.01330897	0.019019	up
TMEM269	2.38791738	0.01330897	0.019019	up
SPTB	2.41932608	1.57E-146	3.00E-145	up
ENDOG	2.43082597	4.97E-39	2.22E-38	up
AOC3	2.43522309	1.04E-05	1.86E-05	up
RAB43	2.43522309	1.04E-05	1.86E-05	up
SBNO2	2.4445009	2.41E-12	5.67E-12	up
EVA1B	2.48102678	5.86E-51	3.27E-50	up
FAM83A	2.48920071	7.33E-10	1.58E-09	up
SCAND1	2.51501735	9.00E-227	3.38E-225	up
FAM222A	2.5254209	1.72E-64	1.20E-63	up
PSG7	2.5254209	0.0073389	0.01075636	up
WFIKKN1	2.5254209	0.0073389	0.01075636	up
OSGIN1	2.54242732	6.28E-57	3.84E-56	up
PPDPF	2.54762856	0	0	up
HIST1H3B	2.56075397	1.41E-10	3.11E-10	up
DLGAP3	2.56848962	5.33E-14	1.32E-13	up
HIST1H1B	2.56848962	1.95E-06	3.62E-06	up
RAB3IL1	2.58955124	8.45E-05	0.00014314	up
SOX15	2.60204218	9.59E-06	1.72E-05	up
PSCA	2.6258608	3.38E-27	1.16E-26	up
C15orf62	2.64547665	3.08E-41	1.43E-40	up
AC008687.4	2.65095178	0.04197239	0.05654853	up
AC116366.3	2.65095178	0.04197239	0.05654853	up
ARIH2OS	2.65095178	0.0040247	0.00605188	up
ARX	2.65095178	0.04197239	0.05654853	up
CSF3	2.65095178	0.04197239	0.05654853	up
IGFBP2	2.65095178	0.04197239	0.05654853	up

LHB	2.65095178	0.04197239	0.05654853	up
NLRP3	2.65095178	0.04197239	0.05654853	up
NXNL2	2.65095178	0.04197239	0.05654853	up
RSPH9	2.65095178	0.0040247	0.00605188	up
SGIP1	2.65095178	0.04197239	0.05654853	up
SPEF1	2.65095178	0.04197239	0.05654853	up
TTC36	2.65095178	0.04197239	0.05654853	up
TTLL9	2.65095178	0.04197239	0.05654853	up
TVP23A	2.65095178	0.04197239	0.05654853	up
RPL36	2.70984547	0	0	up
ARC	2.71806598	2.38E-08	4.83E-08	up
KLF14	2.72895429	0.00023726	0.00039059	up
IL10RB	2.766429	0.00219734	0.00338473	up
NGF	2.766429	0.00219734	0.00338473	up
H2AFJ	2.77482344	3.36E-151	6.70E-150	up
HIST1H2BG	2.7853292	8.31E-75	6.78E-74	up
IL1R2	2.83152403	3.93E-18	1.09E-17	up
FAM207A	2.87051816	4.48E-85	4.14E-84	up
CFAP61	2.8733442	0.02196208	0.03065796	up
DUSP9	2.8733442	0.02196208	0.03065796	up
GATA3	2.8733442	0.02196208	0.03065796	up
GOLGA7B	2.8733442	0.00119531	0.00187787	up
MSLN	2.8733442	0.00119531	0.00187787	up
NUTM2A	2.8733442	0.02196208	0.03065796	up
PDXP	2.8733442	5.00E-20	1.45E-19	up
SCARF2	2.8733442	0.00119531	0.00187787	up
TUBB8P12	2.8733442	0.02196208	0.03065796	up
AL031777.3	2.8733442	3.52E-21	1.05E-20	up
PUF60	2.91101462	6.51E-34	2.61E-33	up
CLEC11A	2.91398619	1.68E-07	3.28E-07	up
HKDC1	2.92397028	2.55E-06	4.70E-06	up
B3GALT5	2.9404584	3.98E-05	6.88E-05	up
GABARAP	2.97287988	0.00064831	0.00103821	up
H2BFS	2.97287988	0.00064831	0.00103821	up
IGF2	2.97287988	0.00064831	0.00103821	up
KIAA1755	2.97287988	0.00064831	0.00103821	up
RNF103-CHM	2.97287988	0.00064831	0.00103821	up
ARL14EPL	3.06598928	0.01148412	0.01654938	up
BNIPL	3.06598928	0.01148412	0.01654938	up
CDH5	3.06598928	1.20E-05	2.13E-05	up
CHRM4	3.06598928	1.59E-08	3.24E-08	up
FOXD4L1	3.06598928	0.01148412	0.01654938	up
LRIG1	3.06598928	0.01148412	0.01654938	up
SDCBP2	3.06598928	4.30E-07	8.24E-07	up
TCTEX1D4	3.06598928	0.01148412	0.01654938	up
COL17A1	3.15345212	1.30E-07	2.56E-07	up
GUCA1B	3.15345212	0.00018944	0.00031416	up
NKD1	3.15345212	0.00018944	0.00031416	up
ABCA9	3.23591428	0.00600663	0.0088921	up
CYP1A1	3.23591428	0.00600663	0.0088921	up
HIST1H4K	3.23591428	8.08E-10	1.74E-09	up
KRT17	3.23591428	3.91E-08	7.85E-08	up
SSC4D	3.23591428	0.00600663	0.0088921	up
HIST1H2BJ	3.23789193	2.03E-268	9.89E-267	up

ABCA6	3.38791738	0.00314448	0.00477135	up
ACOD1	3.38791738	0.03677416	0.04990095	up
ACY1	3.38791738	0.03677416	0.04990095	up
AL355987.3	3.38791738	0.03677416	0.04990095	up
BSCL2	3.38791738	0.03677416	0.04990095	up
C11orf86	3.38791738	0.03677416	0.04990095	up
C20orf204	3.38791738	0.03677416	0.04990095	up
CCR4	3.38791738	0.00314448	0.00477135	up
CELF5	3.38791738	0.03677416	0.04990095	up
CPNE5	3.38791738	0.03677416	0.04990095	up
CRISPLD2	3.38791738	3.49E-09	7.35E-09	up
EBF4	3.38791738	0.03677416	0.04990095	up
EEF1D	3.38791738	0.03677416	0.04990095	up
ESPN	3.38791738	0.03677416	0.04990095	up
GJB4	3.38791738	0.03677416	0.04990095	up
HIST2H4B	3.38791738	0.03677416	0.04990095	up
LGI4	3.38791738	0.03677416	0.04990095	up
PDE1A	3.38791738	0.03677416	0.04990095	up
PIWIL4	3.38791738	0.03677416	0.04990095	up
PODNL1	3.38791738	2.96E-05	5.15E-05	up
PPP1R32	3.38791738	0.03677416	0.04990095	up
RPRML	3.38791738	0.03677416	0.04990095	up
SNCG	3.38791738	0.00314448	0.00477135	up
SOX30	3.38791738	0.00314448	0.00477135	up
TENT5C	3.38791738	0.00314448	0.00477135	up
TEX19	3.38791738	0.03677416	0.04990095	up
TMEM240	3.38791738	0.03677416	0.04990095	up
VWCE	3.38791738	0.03677416	0.04990095	up
VWF	3.38791738	0.03677416	0.04990095	up
HIST1H2AE	3.4445009	6.42E-52	3.62E-51	up
HIST1H2BK	3.45603385	0	0	up
MMP9	3.4583067	1.59E-05	2.81E-05	up
PHOSPHO1	3.4583067	1.59E-05	2.81E-05	up
ITGB2	3.48665133	7.99E-52	4.50E-51	up
DHRS2	3.51344826	3.02E-25	9.90E-25	up
HEY2	3.5254209	0.00164834	0.00256336	up
HIST1H4D	3.56848962	2.71E-08	5.49E-08	up
HIST1H2AM	3.6103098	1.13E-15	2.94E-15	up
HIST1H1C	3.64379364	0	0	up
BEX2	3.65095178	0.01850574	0.02606996	up
C16orf95	3.65095178	0.01850574	0.02606996	up
CAVIN2	3.65095178	0.01850574	0.02606996	up
CCDC153	3.65095178	0.00086549	0.00137204	up
CERS1	3.65095178	0.01850574	0.02606996	up
FA2H	3.65095178	0.01850574	0.02606996	up
HES2	3.65095178	0.01850574	0.02606996	up
IL4I1	3.65095178	0.00086549	0.00137204	up
MATN4	3.65095178	0.01850574	0.02606996	up
RPL7A	3.65095178	0.01850574	0.02606996	up
SESN3	3.65095178	0.00086549	0.00137204	up
SH2D2A	3.65095178	0.01850574	0.02606996	up
SULT2B1	3.65095178	0.01850574	0.02606996	up
ZCCHC12	3.65095178	0.01850574	0.02606996	up
ADAM8	3.766429	7.13E-07	1.35E-06	up

ANGPTL6	3.766429	0.0004553	0.00073577	up
ASB18	3.8733442	0.00940445	0.01367686	up
COL5A3	3.8733442	0.00940445	0.01367686	up
HIST2H2AA4	3.8733442	0.00940445	0.01367686	up
MRM1	3.8733442	0.00940445	0.01367686	up
SHANK3	3.8733442	0.00940445	0.01367686	up
TXNDC5	3.8733442	0.00940445	0.01367686	up
NGFR	3.97287988	3.17E-11	7.17E-11	up
ATP6V0C	4.06598928	6.71E-05	0.00011445	up
FGF22	4.06598928	0.00482189	0.00719293	up
FRMPD1	4.06598928	0.00482189	0.00719293	up
GJA9	4.06598928	0.00482189	0.00719293	up
HIST1H1E	4.06598928	0.00482189	0.00719293	up
HIST2H2AA3	4.06598928	0.00482189	0.00719293	up
HSPB9	4.06598928	0.00482189	0.00719293	up
PRDM12	4.06598928	6.71E-05	0.00011445	up
RRAD	4.06598928	5.04E-12	1.17E-11	up
SPDYE1	4.06598928	0.00482189	0.00719293	up
TMEM151B	4.06598928	0.00482189	0.00719293	up
ROBO4	4.09573662	9.52E-34	3.80E-33	up
SUSD2	4.13637861	4.35E-20	1.27E-19	up
ARSG	4.15345212	3.56E-05	6.16E-05	up
RGMA	4.15345212	5.02E-09	1.05E-08	up
ARID3C	4.23591428	0.00249251	0.00381635	up
DNAH3	4.23591428	1.89E-05	3.34E-05	up
GLIS1	4.23591428	0.00249251	0.00381635	up
HIST1H3J	4.23591428	0.00249251	0.00381635	up
SYT17	4.23591428	0.00249251	0.00381635	up
KIF21B	4.38791738	0.00129816	0.00203393	up
RHBDL1	4.38791738	5.40E-06	9.79E-06	up
SLC28A3	4.38791738	0.00129816	0.00203393	up
SLPI	4.38791738	0.00129816	0.00203393	up
HIST1H4J	4.4583067	2.89E-06	5.31E-06	up
ADRA2C	4.5254209	1.55E-06	2.89E-06	up
BMPER	4.5254209	0.00068087	0.00108851	up
HIST1H2BO	4.5254209	5.13E-37	2.19E-36	up
MC5R	4.5254209	0.00068087	0.00108851	up
SAP25	4.5254209	0.00068087	0.00108851	up
TMEM229B	4.5254209	0.00068087	0.00108851	up
MAP6D1	4.65095178	0.00035947	0.00058476	up
SIGLEC15	4.67085134	1.27E-18	3.57E-18	up
MFNG	4.8733442	0.00010204	0.00017216	up
ADCY10	4.97287988	5.48E-05	9.39E-05	up
KISS1	5.06598928	2.96E-05	5.15E-05	up
DLL4	5.12127172	1.79E-33	7.11E-33	up
HIST2H3D	5.15345212	1.61E-05	2.84E-05	up
SAA1	5.2157364	1.09E-18	3.06E-18	up
CSF2	5.38791738	2.64E-06	4.87E-06	up
RAC2	5.4583067	1.46E-06	2.73E-06	up
