# Supplementary material for: Targeting the KAT8/YEATS4 Axis Represses Tumor Growth and Increases Cisplatin Sensitivity in Bladder Cancer
Source: Adv Sci (Weinh). 2024 Mar 25;11(22):2310146. doi: 10.1002/advs.202310146 (PMC11165526; doi:10.1002/advs.202310146)
Supplement: Supplementary file 3 — Supporting Information [file ADVS-11-2310146-s001.pdf]

ADVANCED SCIENCE
Open Access

Supporting Information

for *Adv. Sci.*, DOI 10.1002/advs.202310146

Targeting the KAT8/YEATS4 Axis Represses Tumor Growth and Increases Cisplatin Sensitivity in Bladder Cancer

Miner Xie, Liwen Zhou, Ting Li, Yujie Lin, Ruhua Zhang, Xianchong Zheng, Cuiling Zeng, Lisi Zheng, Li Zhong, Xiaodan Huang, Yezi Zou, Tiebang Kang and Yuanzhong Wu**

ProteinID	GeneName
O95619	YEATS4
A0A1W2PPV8	KANSL1
P08670	VIM
P25705	ATP5F1A
Q53GZ6	
P52272	HNRNPM
Q8N5Z7	RPL6
P36578	RPL4
P05787	KRT8
Q96DV6	RPS6
E7EQV3	PABPC1
Q53HU0	
A0A024RAY2	KRT18
H6VRF8	KRT1
P35527	KRT9
Q53YD7	EEF1G
A0A1U9X7W4	
B2R6K0	
B7Z4V2	
P13645	KRT10
P15880	RPS2
B4E3A4	
Q9UG63	ABCF2
P46779	RPL28
B7WNR0	ALB
P35908	KRT2
O94776	MTA2
B7Z475	
Q6NZ55	RPL13
P62917	RPL8
P13639	EEF2
Q8IZ29	TUBB2C
Q5SU16	TUBB
A8K2I0	
B4E1T1	
Q02543	RPL18A
Q09028	RBBP4
Q14247	CTTN
Q16891	IMMT
A0A0S2Z4Z9	NONO
Q9HCS7	XAB2
A0A024RD80	HSP90AB1
A0A024QZD1	RPL18
B4DP20	
Q9NVI7	ATAD3A
B4DXZ6	FXR1
J3KTE4	RPL19
Q53HV1	
Q5QTS3	
Q6IQ30	PABPC4

B4DE77	
A0A024R814	RPL7
P17987	TCP1
P04843	RPN1
P02533	KRT14
Q04695	KRT17
P18077	RPL35A
P46781	RPS9
P11021	HSPA5
P67809	YBX1
Q8TCG1	CIP2A
Q8N1G2	CMTR1
A0A0S2Z4Z0	RBM14
P52948	NUP98
A1LUY1	RPL34
A8K0T9	
B3KX11	
Q9UHX1	PUF60
B7Z2E2	
F8W696	APOA1
F6S8Q4	DDX3X
Q6IPT9	EEF1A1
Q8TBW1	
V9HW37	HEL-S-69
Q6IT96	HDAC1
X5D2T3	RPL10
F5H5D3	TUBA1C
P49411	TUFM
O95218	ZRANB2
O94906	PRPF6
Q92769	HDAC2
P05141	SLC25A5
P22695	UQCRC2
P08621	SNRNP70
P27348	YWHAQ
P62910	RPL32
A0A590UK01	SRSF6
A0A024R3D4	DKFZp547C195
Q9BTC8	MTA3
Q5QNW6	H2BC18
Q9UMS4	PRPF19
A8K517	RPS23
P62280	RPS11
B3KTP9	
P21333	FLNA
B2R6J2	
B4DE78	
B4DLQ0	
B4DN41	
B4DNA0	
E9PL09	RPS3

O60251	
Q2TSD0	
E9PLT0	CSDE1
Q53HV2	
J3KQN4	RPL36A
Q59EG8	
B7Z6S8	
B3KRJ9	
Q5JR95	RPS8
R4GMT0	ACTR1A
Q05519	SRSF11
Q96T58	SPEN
P36542	ATP5F1C
O95831	AIFM1
Q86VG2	SFPQ
P31943	HNRNPH1
P42766	RPL35
P62424	RPL7A
Q13347	EIF3I
Q14571	ITPR2
P02545	LMNA
Q15276	RABEP1
P52597	HNRNPF
Q7Z401	DENND4A
Q16666	IFI16
Q86YP4	GATAD2A
Q92499	DDX1
Q9NVA2	SEPTIN11
Q96II8	LRCH3
Q06787	FMR1
Q9Y312	AAR2
Q9Y266	NUDC
A0A024R333	TMEM113
A0A024R326	RPL29
Q9Y383	LUC7L2
A0A087WUW5	PTBP1
A0A087WZT9	PSKH1
A0A087WZK6	DIPK1A
A0A024RBI5	CORO1C
A0A140VJF3	
Q9Y3F4	STRAP
A0A0J9YX62	DNAJB6
A0A2R8YG28	OTOA
A0A140VK07	
A0A1D8GZ55	NUP98
A0A494C039	HYOU1
A0A384MR50	
A0A384N5Y3	MTHFD1
A8K3C3	
A0A2U3TZL8	KIF23
A8K3D0	

A8K9G9	
B3KM36	
B4DGF8	
B4DLP4	
B4DNY3	
B4E014	
B4DRN9	
C6EMX8	MCM7
B7Z4B8	HNRNPUL1
E9PLX7	RPL27A
C9JXB8	RPL24
H0YAS6	PABPC1
H0Y8W2	RACK1
H0Y882	FOXP1
Q2VIM1	
Q15182	SNRPB
H3BPG6	ZNF598
J3QL43	EIF4A1
Q5VU21	
Q59GB4	
M0R210	RPS16
Q8WVA8	
U3KQU7	COG5
Q8IY51	TIGD4
A0N4V7	Tcr-alpha
B3KTW3	
Q5SYB0	FRMPD1
Q9NYP9	MIS18A
Q6UB99	ANKRD11
L0R5A1	CSF2RB
E5RJH3	RPL30
Q96IE3	
B2R4R0	HIST1H4J
E7ETK0	RPS24
A8MY62	LACTBL1
A6NN14	ZNF729
A4D126	CRPPA
O94927	HAUS5
O75628	REM1
O75094	SLIT3
O75952	CABYR
O60242	ADGRB3
O60573	EIF4E2
O94973	AP2A2
O94993	SOX30
O95232	LUC7L3
O95801	TTC4
P10109	FDX1
P17858	PFKL
P22680	CYP7A1
P22234	PAICS

P03951	F11
P01106	MYC
P27816	MAP4
P28289	TMOD1
P04792	HSPB1
P32969	RPL9RPL9P7RPL9P8RPL9P9
P42701	IL12RB1
P11717	IGF2R
P49915	GMPS
P52434	POLR2H
P29475	NOS1
P50552	VASP
P47897	QARS1
P50747	HLCS
P57678	GEMIN4
P54136	RARS1
P61927	RPL37
P62851	RPS25
P78413	IRX4
P62906	RPL10A
P81605	DCD
P61964	WDR5
Q13901	C1D
P78344	EIF4G2
P62195	PSMC5
P62258	YWHAE
Q14155	ARHGEF7
Q01780	EXOSC10
Q13283	G3BP1
Q2TAC6	KIF19
Q15024	EXOSC7
Q16795	NDUFA9
Q5HYK9	ZNF667
Q15370	ELOB
Q16695	H3-4
Q14498	RBM39
Q5T3I0	GPATCH4
Q53EZ4	CEP55
Q5T0F9	CC2D1B
Q5T6S3	PHF19
Q6AW86	ZNF324B
Q5VV41	ARHGEF16
Q5XXA6	ANO1
Q6ZP65	BICDL1
Q71UM5	RPS27L
Q6ZVL6	KIAA1549L
Q7Z5Q1	CPEB2
Q6STE5	SMARCD3
Q86TZ1	TTC6
Q7Z7L1	SLFN11
Q8IXQ6	PARP9

Q8IYW5	RNF168
Q8NDM7	CFAP43
Q8NEE8	TTC16
Q8NFH8	REPS2
Q8NB90	SPATA5
Q8TDI7	TMC2
Q8TEQ0	SNX29
Q8WXD9	CASKIN1
Q8WUJ3	CEMIP
Q96LI9	CXorf58
Q96AG4	LRRC59
Q96LX8	ZNF597
Q96EY7	PTCD3
Q9H2S9	IKZF4
Q96FX7	TRMT61A
Q92831	KAT2B
Q9H4H8	FAM83D
Q9H6L4	ARMC7
Q9HCN4	GPN1
Q9NP60	IL1RAPL2
Q9NTZ6	RBM12
Q9P127	LUZP4
Q9NV70	EXOC1
Q9NWX6	THG1L
Q9NYJ8	TAB2
Q9UHI6	DDX20
Q9ULG1	INO80
Q9Y2V7	COG6
A0A024R3V9	
Q9Y5J1	UTP18
Q9Y597	KCTD3
A0A024R0H6	PAF1
Q9Y388	RBMX2
Q9Y3B4	SF3B6
A0A024R6U8	MMP15
A0A024R7X1	TERF1
A0A024R845	RAB14
A0A024R8H9	
A0A024R861	PPP6C
A0A024RCW7	DOM3Z
A0A024RBX2	FAM51A1
A0A024RAD5	DDOST
A0A024RB35	C1QDC1
A0A087WWI6	DCAF7
A0A087WSW9	TXNRD1
A0A087WZK8	SHISA5
A0A087X0A2	ZNF254
A0A024R4V5	BRD1
A0A087WWP4	RBM15
A0A096LNQ7	ERCC6
A0A0A0MRA9	RGS6

A0A0D9SEJ5	FAM120B
A0A0D9SFI4	R3HCC1
A0A0G2JM88	MCCC2
A0A0D9SFE5	LMNB1
A0A0S2Z5A5	SEPT9
A0A0S2Z500	OPTN
A0A0S2Z5K5	ZCWPW1
A0A0U1RQT6	DOCK7
A0A0A0MRA3	TTN
A0A140VJH7	
A0A0U1RQU9	IL20RA
A0A140VJS9	
A0A140VJT0	
A0A192GP51	MYD88
A0A1L2BU42	
A0A0K0K1K8	HEL-S-17
A0A1B0GV63	ARID1B
A0A1B0GUL6	BTBD8
A0A2R8Y7N6	TRANK1
A0A1W2PS00	SYN1
A0A2R8Y623	RPS7
A0A384NYG5	MED17
A0A384NPQ2	
A0A3B3ITM2	COL17A1
A0A2R8Y7U4	SMARCE1
A0A3B3ITR5	SLC35D2
A0A3G6V326	SMN1
A0A2R8YDI5	ATP10B
A0A3B3IU69	EML1
A0A286YEX5	CHCHD3
A0A5C2GNQ1	
A0A5F9ZI70	C3orf49
A0A494C1A5	SNRNP200
A0A499FIZ0	WDR26
A0N7E2	V-alpha-13
A2A299	TGM2
A1DRY3	
A5JUM9	APC
A2A341	SYCP2
A8K564	
A8K0Y0	OPCML
A8K5M4	
A8K5Q1	
A8K9U6	
A8K9V3	
A8KAN9	
B2R8P6	
B2RBV7	
B3KMJ1	
B3KN45	
B0YIW6	ARCN1

B3KU03	
B3KT25	
B3KXS7	
B4DEZ5	
B4DHC5	
B4DF22	
B4DHW5	
B4DJW8	
B4DLR3	
B4DMB7	
B4DMU7	
B4DMI9	
B4DQ43	
B4DTH8	
B4DX64	
B4DXQ5	
B4DYP7	
B4DWY7	ADAM2
B4DZC3	
B4E3R4	
B5BU53	CDK9
B7Z761	
B7Z2V6	
B7ZA33	
B7ZB55	
C9IZY8	FMNL2
C9J1Z8	ARF5
D6R9I9	ABCE1
D6RBZ3	TECRL
D6RFJ3	EIF4E
C9J2Q4	SEPTIN2
C9J7T9	TNNC2
C9JA93	TBC1D15
E5RG59	ZNF395
E5RGJ0	GABRG2
E5RGU4	EIF3H
E5RHL3	FADS2
D6W540	GPR113
E5RJD8	TBCA
E5RHK8	DNM3
E7ESL9	AGAP3
E7ETR9	-
E7EUW0	CCSER1
E7ESW6	WDR87
E7ER40	PHF3
E9PAU2	RAVER1
E9PFD2	UMPS
E9PIW9	SAMD1
E9PI41	EXOSC4
E9PRJ8	CD81
F4MHG5	UTY

F5GWH2	ACSL3
E9PQ49	EEF1D
F5H026	ZNF880
E9PHM6	DST
G3XFS1	
G3V2M5	FCF1
H0Y5D2	GTF3C5
F5GYN0	FAM186A
H0Y8X1	SDHA
H0YCZ7	COL24A1
H0Y6P9	PDE11A
H0YDE6	DENND5A
H0YG19	ADD1
H0YF29	C8orf82
H0YKU6	SLTM
H3BS59	TUBGCP4
H3BNH8	
H0YKV0	IVD
H3BND0	TEDC2
H3BVC7	RPS15A
H7BZA8	RALGAPA2
H3BLS7	VPS13D
H7C371	MLPH
H3BV11	NUP93
H7C276	PLCL2
H7C573	ACOX2
H7C3D7	AEBP1
H7C3J9	GAB3
I3L1Q5	TSR1
H7C5F2	MRPS22
I3L495	PAFAH1B1
I3L3I7	PLD2
I3L3P7	RPS15A
I3NI24	GLOD4
K7ELG9	LSM12
K7EMM5	AFMID
K7ENL6	FBF1
J3QLF0	GSDMB
J7HWJ8	CYTB
K7EQ61	HAUS5
J3QLC8	RPL17
L0B3M6	REST
J3KN66	TOR1AIP1
K7ER00	FARSA
P78517	
Q05D84	HMMR
M0QYN0	MYDGF
M0R0J4	DHPS
M0QYL6	ZNF814
Q14918	
Q2TAM6	RUNX1

Q05BG8	CCDC24
Q3KNT6	FLJ11235
Q4G104	FBXO21
Q59F66	
Q59FA6	
Q59ET3	
Q504U3	PKM2
Q59FI9	
Q53T09	XRCC5
Q59HB3	
Q59GK9	
Q5J8M4	
Q658M9	DKFZp666K145
Q68DC6	CBFA2T2
Q6NXR8	RPS3A
Q5JSD2	VDAC2
Q5T6L4	ASS
Q6AZ94	LTBP2
Q6PIW8	COG4
Q6ZNS1	FLJ27255
Q7Z3B7	DKFZp451N061
Q6ZNU1	
Q86YQ0	HZGJ
Q8IUN0	SMPD1
Q8WYE4	DMD
Q9BSM5	
Q8N4A2	CYorf15B
Q8IWI5	
Q9H4E3	E4-DBP
Q9H7J2	FLJ00087
Q8N5A0	EIF5B
Q9P0C7	
V9GYC1	APOA2
V9GZZ7	
R4GNB3	ANKRD18B
Q9UFW7	DKFZp566O183
V9PAJ5	NBS1
V9HWC1	HEL71
V9HWJ0	HEL-S-164nA
X6RAJ1	AKAP17A
